# Correction: Do religious beliefs influence concerns for animal welfare? the role of religious orientation and ethical ideologies in attitudes toward animal protection amongst Muslim teachers and school staff in East Java, Indonesia

**DOI:** 10.1371/journal.pone.0301792

**Published:** 2024-04-02

**Authors:** Dexon Pasaribu, Pim Martens, Bagus Takwin

There is an error in the article title. The correct title is: Do religious beliefs relate to concerns for animal welfare? Religious orientation and ethical ideologies correlation to attitudes toward animal protection amongst Muslim teachers and school staff in East Java, Indonesia. The correct citation is: Pasaribu D, Martens P, Takwin B (2021) Do religious beliefs relate to concerns for animal welfare? Religious orientation and ethical ideologies correlation to attitudes toward animal protection amongst Muslim teachers and school staff in East Java, Indonesia. PLoS ONE 16(7): e0254880. https://doi.org/10.1371/journal.pone.0254880.
