# Supplementary material for: Do religious beliefs influence concerns for animal welfare? the role of religious orientation and ethical ideologies in attitudes toward animal protection amongst Muslim teachers and school staff in East Java, Indonesia
Source: PLoS One. 2021 Jul 16;16(7):e0254880. doi: 10.1371/journal.pone.0254880 (PMC8284611; doi:10.1371/journal.pone.0254880)
Supplement: S1 Appendix — (DOCX) [file pone.0254880.s001.docx]

# S1 Appendix: Questionnaires in their original language

## **Data control**

| 1. What is your birth-year? ____________ | |
| --- | --- |
| 1. What is your sex? Female/Male | |
| 1. In what country do you live? ___________________________________ | |
| 1. What is the highest level of schooling you have completed? | |
| - 1. No education   2. Less than grade 12   3. High school   4. College or technical school | - 1. University   2. No answer   3. Other |
| 1. Do you belong or donate to an organization or charity involved in or concerned with: | |
| - 1. Improving the welfare of animals Yes/No   2. Conservation of the natural environment Yes/No | - 1. Improving human rights or health Yes/No |
| 1. How does your household look like? | |
| - 1. Single without children   2. Single with children   3. Married/living with partner without children | - 1. Married/living with partner with children   2. Other |
| 1. Where is your current residence place? | |
| - 1. Urban areas (a geographical area constituting a city or town)   2. Rural areas (an area outside of cities and towns) | |
| 1. In what sort of house do you live? | |
| - 1. Apartment/flat   2. Semi-detached house   3. Detached house | |
| 1. Do you have a garden? Yes/No | |
| 1. What is your occupation? | |
| - 1. Liberal profession   2. Employed   3. Retired   4. Student-scholarship | - 1. Social welfare   2. No answer   3. Other |
| 1. Is religion/spirituality important in your life? Yes/No | |
| ***If your answer is yes***, then what is your main source of inspiration (Multiple answers possible)? | |
| - 1. Buddhism   2. Judaism   3. Islam | - 1. Christianity   2. Taoism   3. Other |
| 1. What is your gross household income per month? | |
| - 1. Below the minimum wage in your country   2. The minimum wage in your country   3. The average income in your country | - 1. About twice the average income in your country   2. More than twice the average income in your country   3. No answer |
| 1. How often do you eat meat (including fish) every week? | |
| - 1. I do not eat meat   2. Once a week   3. 2-3 days a week | - 1. 4-6 days a week   2. Every day |
| 1. Do you own a pet? Yes/No | |
| ***If your answer is yes***, what pet do you have (Multiple answers possible)? | |
| - 1. Cat(s)   2. Dog(s)   3. Fish   4. Birds   5. Reptiles | - 1. Rodents   2. Chickens, pigeon, geese (or other poultry)   3. Ponies, horses   4. Other: ... |
| 1. How often do you visit a zoo or aquarium? | |
| - 1. Once a month   2. Once every six month   3. Once every year | - 1. Once two years or more than two years   2. Never |

## **The Animal Attitude Scale (AAS)**

On a scale from 1 (strongly disagree) to 5 (strongly agree), please indicate how much you agree or disagree to a statement according below degree:

| 1 | 2 | 3 | 4 | 5 |
| --- | --- | --- | --- | --- |
| Strongly Disagree | Disagree | Undecided | Agree | Strongly Agree |

Example:

Buying clothes made from animal fur __________

*If you ‘disagree’ with above statement, you write ‘2’*

Buying clothes made from animal fur _____2____

**Note:** The higher the number means the more you agree with the statement.

| 1. It is morally wrong to hunt wild animals just for sport. | ____ |
| --- | --- |
| 1. I do not think that there is anything wrong with using animal in medical research. | ____ |
| 1. There should be extremely stiff penalties including jail sentences for people who participate in cock-fighting. | ____ |
| 1. Wild animals, such as mink and raccoons, should not be trapped and their skins made into fur coats. | ____ |
| 1. There is nothing morally wrong with hunting wild animals for food or a better living for poor people. | ____ |
| 1. I think people who object to raising animals for meat are too sentimental. | ____ |
| 1. Much of the scientific research done with animals is unnecessary and cruel. | ____ |
| 1. I think it is perfectly acceptable for cattle and dogs to be raised for human consumption. | ____ |
| 1. Basically, humans have the right to use animals as we see fit. | ____ |
| 1. The slaughter of whales and dolphins should be immediately stopped even if it means some people will be put out of work. | ____ |
| 1. I sometimes get upset when I see wild animals in cages at zoos. | ____ |
| 1. In general, I think that human economic gain is more important than setting aside more land for wildlife. | ____ |
| 1. Too much fuss is made over the welfare of animals these days when there are many human problems that need to be solved. | ____ |
| 1. Breeding animals for their skins is a legitimate use of animals. | ____ |
| 1. Some aspects of biology can only be learned through dissecting preserved animals, such as cats. | ____ |
| 1. Continued research with animals will be necessary if we are to ever conquer diseases such as cancer, heart disease and AIDS. | ____ |
| 1. It is unethical to breed purebred dogs for pets when millions of dogs are killed in animal shelters each year. | ____ |
| 1. The production of inexpensive meat, eggs, and dairy products justifies maintaining animals under crowded conditions. | ____ |
| 1. The use of animals, such as rabbits, for testing the safety of cosmetics and household products is unnecessary and should be stopped. | ____ |
| 1. The use of animals in rodeos and circuses is cruel. | ____ |

## **The Animal Issue Scale (AIS)**

On a scale from 1 (extremely unacceptable) to 5 (extremely acceptable), please indicate how much you can accept or reject a statement according below degree:

| 1 | 2 | 3 | 4 | 5 |
| --- | --- | --- | --- | --- |
| Extremely unacceptable | Unacceptable | Normal | Acceptable | Extremely acceptable |

Example:

I let people mock me sometimes __________

*If you feel that this statement is ‘unacceptable’, you write ‘2’*

I let people mock me sometimes _____2____

**Note:** The higher the number means the more you accept the statement.

| **1.1: Use of animals** |  |
| --- | --- |
| 1 Keeping animals for the production of food or clothing | ____ |
| 2 Keeping animals as pets | ____ |
| 3 Keeping animals for the education of the public in zoos, wildlife parks, etc | ____ |
| 4 Using animals for work | ____ |
| 5 Using animals for entertainment or sports | ____ |
|  |  |
| **1.2: Animal integrity** |  |
| 6 Operations on animals to improve their health | ____ |
| 7 Decoration of animals, such as dyeing or cutting their hair for aesthetic reasons | ____ |
| 8 De-sexing by hormone implants | ____ |
| 9 Removal of a body part, such as tail docking or de-clawing | ____ |
| 10 Marking animals by branding or ear notching | ____ |
| 11 Removal of dead tissue, such as hair/wool removal or foot trimming | ____ |
|  |  |
| **1.3: Killing animals** |  |
| 12 Killing young animals that are dependent on their parents | ____ |
| 13 Allowing animals to experience pain during slaughter | ____ |
| 14 Using animals for products after their natural death | ____ |
| 15 Killing animals when they are seriously injured or ill | ____ |
| 16 Euthanising healthy and unwanted pets because of overpopulation | ____ |
|  |  |
| **1.4: Animal welfare** |  |
| 17 Depriving animals of their needs for food and water | ____ |
| 18 Depriving animals of an appropriate environment to rest, including shelter | ____ |
| 19 Inflicting pain, injury or disease on animals | ____ |
| 20 Not providing sufficient space, proper facilities and company needed for animals | ____ |
| 21 Subjecting animals to conditions and treatment which cause mental suffering | ____ |
|  |  |
| **1.5: Experimentation on animals** |  |
| 22 Observing animal behaviour in an experiment | ____ |
| 23 Experiments to improve animal welfare or health | ____ |
| 24 Medical experiments using animals to improve human health | ____ |
| 25 Testing cosmetics or household products on animals | ____ |
| 26 Operating on living animals for the benefits of human medicine research | ____ |
|  |  |
| **1.6: Changes in animals’ genotypes** |  |
| 27 Increasing animals’ reproductive or productive capabilities by genetic changes, eg cows producing more milk | ____ |
| 28 Increasing animals’ health or disease resistance by genetic changes | ____ |
| 29 Creating farm animals that are more profitable because they feel happy with little stimulation and have little desire to be active | ____ |
| 30 Genetic selection of pet animals, such as dogs and cats, to increase their rarity, potential for showing or pedigree value | ____ |
| 31 Genetic modification of crops grown for animal foods | ____ |
|  |  |
| **1.7: Animals and the environment** |  |
| 32 Killing animals because they are not native to the area where they live | ____ |
| 33 Killing wild animals to stop the spread of diseases that could affect humans | ____ |
| 34 Controlling wildlife populations by killing | ____ |
| 35 Controlling animal populations by sterilisation | ____ |
| 36 Destroying the habitat of endangered animal species | ____ |
| 37 Destroying the habitat of non-endangered animal species to develop and promote urbanisation or crops to feed humans | ____ |
|  |  |
| **1.8: Societal attitudes towards animals** |  |
| 38 Sacrifice of animals in religious rites | ____ |
| 39 Considering some animal species as sacred or good luck symbols or totems | ____ |
| 40 Considering some animal species as evil or bad luck | ____ |
| 41 Parents displaying cruel treatment of animals in front of their children | ____ |
| 42 Inflicting pain or injury on animals as part of cultural traditions | ____ |
| 43 Cloning animals for human benefit | ____ |

## **The Religious Orientation Scale (ROS)**

On a scale from 1 (strongly disagree) to 5 (strongly agree), please indicate how much you agree or disagree to a statement according below degree:

| 1 | 2 | 3 | 4 | 5 |
| --- | --- | --- | --- | --- |
| Strongly Disagree | Disagree | Undecided | Agree | Strongly Agree |

Example:

I never forget to lock the door __________

*If you ‘agree’ with above statement, you write ‘4’*

I never forget to lock the door _____4____

**Note**: The higher the number means the more likely you agree with the statement.

| 1. I try hard to live all my life according to my religious beliefs | ____ |
| --- | --- |
| 1. It doesn’t much matter what I believe so long as I am good | ____ |
| 1. I have often had a strong sense of God’s presence | ____ |
| 1. My whole approach to life is based on my religion | ____ |
| 1. Prayers I say when I’m alone are as important as those I say in church | ____ |
| 1. I attend church once a week or more | ____ |
| 1. My religion is important because it answers many questions about the meaning of life | ____ |
| 1. I enjoy reading about my religion | ____ |
| 1. It is important to me to spend time in private thought and prayer | ____ |
| 1. What religion offers me most is comfort in times of trouble and sorrow | ____ |
| 1. Prayer is for peace and happiness | ____ |
| 1. I pray mainly to gain relief and protection | ____ |
| 1. I go to church because it helps me make friends | ____ |
| 1. I go to church mainly because I enjoy seeing people I know there | ____ |
| 1. I go to church mostly to spend time with my friends | ____ |

## **The Ethical Position Questionnaire (EPQ)**

On a scale from 1 (Completely disagree) to 9 (Completely agree), please indicate how much you agree or disagree to a statement according below degree:

| 1 | 2 | 3 | 4 | 5 | 6 | 7 | 8 | 9 |
| --- | --- | --- | --- | --- | --- | --- | --- | --- |
| Completely Disagree | Largely Disagree | Moderately Disagree | Slightly Disagree | Neither agree not disagree | Slightly Agree | Moderately Agree | Largely Agree | Completely Agree |

Example:

I never forget to lock the door __________

*If you ‘largely agree’ with above statement, you write ‘8’*

I never forget to lock the door _____8____

**Note**: The higher the number means the more you agree with the statement.

| 1. People should make certain that their actions never intentionally harm another even to a small degree. | ____ |
| --- | --- |
| 1. Risks to another should never be tolerated, irrespective of how small the risks might be. | ____ |
| 1. The existence of potential harm to others is always wrong, irrespective of the benefits to be gained. | ____ |
| 1. One should never psychologically or physically harm another person. | ____ |
| 1. One should not perform an action which might in any way threaten the dignity and welfare of another individual. | ____ |
| 1. If an action could harm an innocent other, then it should not be done. | ____ |
| 1. Deciding whether or not to perform an act by balancing the positive consequences of the act against the negative consequences of the act is immoral. | ____ |
| 1. The dignity and welfare of the people should be the most important concern in any society. | ____ |
| 1. It is never necessary to sacrifice the welfare of others. | ____ |
| 1. Moral behaviors are actions that closely match ideals of the most “perfect” action. | ____ |
| 1. There are no ethical principles that are so important that they should be a part of any code of ethics. | ____ |
| 1. What is ethical varies from one situation and society to another. | ____ |
| 1. Moral standards should be seen as being individualistic; what one person considers to be moral may be judged to be immoral by another person. | ____ |
| 1. Different types of morality cannot be compared as to “rightness.” | ____ |
| 1. Questions of what is ethical for everyone can never be resolved since what is moral or immoral is up to the individual. | ____ |
| 1. Moral standards are simply personal rules that indicate how a person should behave, and are not be be applied in making judgments of others. | ____ |
| 1. Ethical considerations in interpersonal relations are so complex that individuals should be allowed to formulate their own individual codes. | ____ |
| 1. Rigidly codifying an ethical position that prevents certain types of actions could stand in the way of better human relations and adjustment. | ____ |
| 1. No rule concerning lying can be formulated; whether a lie is permissible or not permissible totally depends upon the situation. | ____ |
| 1. Whether a lie is judged to be moral or immoral depends upon the circumstances surrounding the action. | ____ |
