# Supplementary material for: Do religious beliefs influence concerns for animal welfare? the role of religious orientation and ethical ideologies in attitudes toward animal protection amongst Muslim teachers and school staff in East Java, Indonesia
Source: PLoS One. 2021 Jul 16;16(7):e0254880. doi: 10.1371/journal.pone.0254880 (PMC8284611; doi:10.1371/journal.pone.0254880)
Supplement: S2 Appendix — (DOCX) [file pone.0254880.s002.docx]

# S2 Appendix: Questionnaires translated to Bahasa Indonesia

## **Kontrol Data**

Isilah pertanyaan-pertanyaan di bawah ini.

Jika pertanyaan berbentuk pilihan, lingkari jawaban yang sesuai dengan anda.

Jika pertanyaan berbentuk isian maka isilah dengan singkat

| 1. Jenis kelamin: **Perempuan/Laki-laki** (lingkari salah satu) | |
| --- | --- |
| 1. Apakah anda anggota ATAU penyumbang organisasi/ormas keagamaan? **Ya/Tidak** (lingkari salah satu) | |
| ***Jika anda menjawab “ya”***, Bergerak di bidang apa organisasi anda? (bisa melingkari lebih dari satu) | |
| - 1. Dakwah   2. Sumbangan/derma   3. Politik   4. Jurnalistik Berita/Penggiat Media Sosial | - 1. Yayasan/Lembaga Sosial Masyarakat (LSM) kemanusiaan   2. Peningkatan ekonomi (contoh: arisan, koperasi, dll)   3. Lain-lain: __________________________ (tuliskan) |
| 1. Di sekolah mana anda bekerja: (isi dengan singkat nama sekolah dan domisili sekolah)   _____________________________________________________________________________________ | |
| 1. Tahun berapa anda lahir? ____________ (tulis keseluruhan 4 angka, contoh: 1989) | |
| 1. Dimana anda tinggal?   Propinsi: ___________________________________, Kabupaten: _______________________________ | |
| 1. Jenjang pendidikan yang telah ditamatkan? (lingkari salah satu di bawah ini) | |
| - 1. Tidak sekolah   2. SD/Madrasah Ibtidayah   3. SMP/Madrasah Tsanawiyah   4. SMU/Madrasah Aliyah | - 1. Diploma D1/D3   2. Strata Pertama (S1)   3. S2/S3   4. Lain-lain: ________________ (tuliskan) |
| 1. Apakah anda anggota atau penyumbang organisasi sosial yang bergerak di bidang di bawah ini: | |
| - 1. Peningkatan kesejahteraan binatang **Ya/Tidak**   2. Pelestarian lingkungan hidup/alam **Ya/Tidak** | - 1. Peningkatan kesehatan/hak asasi manusia **Ya/Tidak** |
| 1. A. Bagaimana status pernikahan anda? (lingkari salah satu di bawah ini) | |
| - 1. Lajang/belum menikah   2. Menikah | - 1. Janda/Duda |
| B. Apakah anda mempunyai anak? **Ya/Tidak** (lingkari salah satu) | |
| 1. Didaerah apa anda bermukim? | |
| - 1. Daerah Perkotaan (termasuk kota kecil)   2. Di luar kawasan kota seperti Desa/Dusun | |
| 1. Jenis tempat tinggal anda? (lingkari salah satu di bawah ini) | |
| - 1. Apartment   2. Kost-kostan/Kontrakan | - 1. Rumah sendiri   2. Rumah bersama orang tua |
| 1. Apakah anda mempunyai kebun/taman? **Ya/Tidak** (lingkari salah satu) | |
| 1. Apa pekerjaan utama anda? | |
| - 1. Wiraswasta   2. Pegawai Swasta   3. Pegawai Negeri Sipil (PNS)   4. Pensiun   5. Pelajar/Beasiswa | - 1. Pekerja sosial   2. Peneliti/dosen   3. Bertani/beternak   4. Tidak ingin menjawab   5. Lain-lain: ________________ (tuliskan) |
| 1. A. Apakah Agama anda? __________________________ (boleh tidak menjawab) | |
| B. Suku anda: __________________________ (boleh tidak menjawab) | |
| C. Apakah Agama/Spiritualitas penting dalam hidup anda? Ya/Tidak (lingkari salah satu) | |
| ***Jika anda menjawab “ya”***, Agama apakah yang menginspirasi anda? (bisa menjawab lebih dari satu) | |
| - 1. Budha   2. Hindu   3. Islam | - 1. Kristen   2. Aliran kepercayaan lain: ______________ (tuliskan) |
| 1. A. Seberapa besar pemasukan per bulan anda? (lingkari salah satu di bawah ini) | |
| - 1. Dibawah standar gaji minimum di negara saya   2. Sebesar standar gaji minimum di negara saya   3. Sebesar standar gaji rata-rata di negara saya | - 1. Dua kali lipat standar gaji rata-rata di negara saya   2. Lebih dari dua kali lipat standar gaji rata-rata di negara saya   3. Tidak menjawab |
| B. Seberapa besar pengeluaran per bulan anda? | |
| - 1. Kurang dari Rp. 5.000.000,-   2. Antara Rp. 5.000.001,- sampai Rp. 10.000.000,-   3. Antara Rp.10.000.001,- sampai Rp. 15.000.000,- | - 1. Antara Rp. 15.000.001,- sampai Rp. 25.000.000,-   2. Diatas Rp. 25.000.001,-   3. Tidak ingin menjawab |
| 1. Seberapa sering anda memakan daging (termasuk ikan) dalam seminggu? (lingkari salah satu di bawah ini) | |
| - 1. Saya tidak makan daging   2. Sekali seminggu   3. 2-3 hari dalam seminggu | - 1. 4-6 hari dalam seminggu   2. Setiap hari |
| 1. Apakah anda mempunyai hewan peliharaan? **Ya/Tidak** (lingkari salah satu) | |
| ***Jika anda menjawab “ya”***, hewan peliharaan apa yang anda punya? (bisa menjawab lebih dari satu) | |
| - 1. Kucing   2. Anjing   3. Ikan   4. Burung   5. Reptil | - 1. Sejenis tikus (contoh: tikus putih, marmut, hamster, dll)   2. Ayam, Bebek/Angsa, dll   3. Kuda   4. Lain-lain: ________________ (tuliskan) |
| 1. Seberapa sering anda mengunjungi kebun binatang atau akuarium umum? (lingkari salah satu di bawah ini) | |
| - 1. Sekali sebulan   2. Sekali dalam enam bulan   3. Sekali setahun | - 1. Sekali dalam dua tahun atau lebih dari dua tahun   2. Tidak pernah |

## **Animal Attitude Scale (AAS)**

Berikan angka 1 (satu) sampai dengan 5 (lima) untuk menunjukkan setuju atau tidaknya anda terhadap suatu pernyataan menurut jenjang di bawah ini:

| 1 | 2 | 3 | 4 | 5 |
| --- | --- | --- | --- | --- |
| Sangat tidak setuju | Tidak setuju | Ragu-ragu | Setuju | Sangat setuju |

**Catatan:** Semakin tinggi angka yang anda bubuhkan berarti semakin setuju anda dengan pernyataan itu.

| 1. Secara moral, berburu binatang liar hanya untuk olahraga itu salah. | ____ |
| --- | --- |
| 1. Saya tidak berpikir tidak ada yang salah dengan menggunakan hewan dalam penelitian medis. | ____ |
| 1. Harus ada hukuman yang sangat ketat dan hukuman penjara bagi orang-orang yang berpartisipasi dalam laga ayam. | ____ |
| 1. Hewan liar, seperti cerpelai dan musang sebaiknya tidak boleh diperangkap dan dibuat kulitnya menjadi mantel bulu. | ____ |
| 1. Secara moral tiada yang salah dengan berburu hewan liar untuk makanan atau kehidupan yang lebih baik untuk orang miskin. | ____ |
| 1. Saya pikir orang yang keberatan memelihara hewan untuk daging pangan itu terlalu sentimental. | ____ |
| 1. Sebagian besar penelitian ilmiah menggunakan hewan itu kejam dan tidak perlu. | ____ |
| 1. Saya pikir sangat dapat diterima ternak dikembang-biakan untuk konsumsi manusia. | ____ |
| 1. Pada dasarnya, manusia berhak untuk menggunakan hewan sesuai kebutuhannya. | ____ |
| 1. Meskipun bisa membuat penduduk kehilangan mata pencaharian, pembantaian paus dan lumba-lumba harus segera dihentikan. | ____ |
| 1. Kadang saya kesal saat melihat binatang liar di kandang kebun binatang. | ____ |
| 1. Secara umum, saya berpikir bahwa keuntungan ekonomi manusia lebih penting daripada menyisihkan lebih banyak lahan untuk satwa liar. | ____ |
| 1. Akhir-akhir ini, terlalu banyak kerepotan ditujukan untuk kesejahteraan hewan dibandingkan banyaknya masalah manusia yang perlu dipecahkan, | ____ |
| 1. Pembiakan hewan untuk kulit mereka adalah penggunaan hewan yang sah. | ____ |
| 1. Beberapa aspek biologi hanya bisa dipelajari melalui pembedahan hewan yang diawetkan, contohnya seperti kucing. | ____ |
| 1. Kelanjutan penelitian dengan hewan akan diperlukan jika kita ingin menaklukkan penyakit seperti kanker, penyakit jantung dan AIDS. | ____ |
| 1. Tidak etis membiakkan anjing ras untuk hewan peliharaan dikala jutaan anjing terbunuh di tempat penampungan hewan setiap tahunnya. | ____ |
| 1. Produksi daging, telur, dan produk susu yang murah membenarkan pemeliharaan hewan dalam kondisi yang terlalu sesak dan padat. | ____ |
| 1. Penggunaan hewan, seperti kelinci, untuk pengujian keamanan kosmetik dan produk rumah tangga itu tidaklah perlu dan harus dihentikan. | ____ |
| 1. Penggunaan hewan untuk atraksi dan sirkus itu kejam. | ____ |

## **The Animal Issue Scale (AIS)**

Berikan angka 1 (satu) sampai dengan 5 (lima) untuk menunjukkan dapat atau tidaknya anda menerima suatu pernyataan menurut jenjang dibawah ini:

| 1 | 2 | 3 | 4 | 5 |
| --- | --- | --- | --- | --- |
| Sangat tidak bisa diterima | Tidak bisa diterima | Ragu-ragu | Bisa diterima | Sangat bisa diterima |

**Catatan**: Semakin tinggi angka yang anda bubuhkan berarti semakin anda dapat menerima pernyataan itu.

| 1 Memelihara hewan untuk produksi makanan atau pakaian | ____ |
| --- | --- |
| 2 Hewan sebagai hewan peliharaan | ____ |
| 3 Memelihara hewan untuk pendidikan masyarakat di kebun binatang, taman margasatwa, dll. | ____ |
| 4 Menggunakan hewan untuk bekerja | ____ |
| 5 Menggunakan hewan untuk hiburan atau olahraga | ____ |
| 6 Melakukan operasi pada hewan untuk memperbaiki kesehatan hewan itu. | ____ |
| 7 Dekorasi hewan, seperti mewarnai atau memotong rambut mereka untuk alasan estetika | ____ |
| 8 Mengurangi seksualitas hewan dengan implan hormon | ____ |
| 9 Menghilangkan bagian tubuh, seperti pemotongan ekor atau kuku | ____ |
| 10 Menandai hewan dengan merk atau tindik kuping | ____ |
| 11 Menghilangkan jaringan mati, seperti menghilangkan rambut/wol atau pemangkasan kaki | ____ |
| 12 Membunuh hewan muda yang bergantung pada orang tua mereka | ____ |
| 13 Memungkinkan hewan mengalami rasa sakit saat disembelih | ____ |
| 14 Menggunakan mayat hewan sebagai produk setelah kematian alami mereka | ____ |
| 15 Membunuh hewan saat mereka terluka parah atau sakit | ____ |
| 16 Membunuh hewan peliharaan yang sehat dan tidak diinginkan karena kelebihan populasi | ____ |
| 17 Merampas hewan dari kebutuhan mereka akan makanan dan air | ____ |
| 18 Merampas hewan dari lingkungan yang layak untuk bernaung, termasuk tempat berlindung | ____ |
| 19 Menimbulkan rasa sakit, luka atau penyakit pada hewan | ____ |
| 20 Tidak menyediakan ruang yang cukup, fasilitas yang memadai dan kebersamaan dengan hewan lain yang dibutuhkan hewan | ____ |
| 21 Mengijinkan pada kondisi dan perawatan yang menyebabkan penderitaan mental | ____ |
| 22 Mengamati perilaku hewan dalam percobaan | ____ |
| 23 Percobaan untuk meningkatkan kesejahteraan atau kesehatan hewan | ____ |
| 24 Percobaan medis menggunakan hewan untuk memperbaiki kesehatan manusia | ____ |
| 25 Menguji kosmetik atau produk rumah tangga pada binatang | ____ |
| 26 Mengoperasikan hewan hidup untuk manfaat penelitian obat manusia | ____ |
| 27 Meningkatkan kemampuan produktif dan reproduksi hewan melalui perubahan genetik, misalnya sapi yang menghasilkan lebih banyak susu | ____ |
| 28 Meningkatkan ketahanan penyakit atau kesehatan hewan melalui pengubahan genetiknya | ____ |
| 29 Menciptakan hewan ternak yang lebih menguntungkan karena menganggap mereka (hewan) merasa senang dengan sedikitnya kegiatan dan keinginan untuk aktif | ____ |
| 30 Pemilihan genetik hewan peliharaan, seperti anjing dan kucing, untuk meningkatkan kelangkaannya atau meningkatkan nilai silsilah asal-usulnya. | ____ |
| 31 Modifikasi genetik tanaman untuk makanan hewani | ____ |
| 32 Membunuh hewan karena mereka bukan hewan asli di linkungan tinggal mereka | ____ |
| 33 Membunuh hewan liar untuk menghentikan penyebaran penyakit yang bisa menyerang manusia | ____ |
| 34 Mengendalikan populasi satwa liar dengan membunuh | ____ |
| 35 Mengontrol populasi hewan melalui sterilisasi | ____ |
| 36 Menghancurkan linkungan habitat spesies hewan yang terancam punah | ____ |
| 37 Menghancurkan lingkungan habitat spesies hewan yang tidak terancam punah guna mengembangkan dan mempromosikan urbanisasi atau tanaman pangan untuk memberi makan manusia | ____ |
| 38 Pengorbanan binatang dalam upacara keagamaan | ____ |
| 39 Mempertimbangkan beberapa spesies hewan sebagai simbol kesucian atau keberuntungan. | ____ |
| 40 Mempertimbangkan beberapa spesies hewan sebagai jahat atau nasib buruk | ____ |
| 41 Orangtua menampilkan perlakuan kejam terhadap hewan di depan anak-anak mereka | ____ |
| 42 Menimbulkan rasa sakit atau luka pada hewan sebagai bagian dari tradisi budaya | ____ |
| 43 Menduplikasi hewan untuk keuntungan manusia | ____ |

## **The Religious Orientation Scale (ROS)**

Berikan angka 1 (satu) sampai dengan 5 (lima) untuk menunjukkan setuju atau tidaknya anda terhadap suatu pernyataan menurut jenjang di bawah ini:

| 1 | 2 | 3 | 4 | 5 |
| --- | --- | --- | --- | --- |
| Sangat tidak setuju | Tidak setuju | Ragu-ragu | Setuju | Sangat setuju |

**Catatan:** Semakin tinggi angka yang anda bubuhkan berarti semakin setuju anda dengan pernyataan itu.

| 1. Saya berusaha keras untuk menjalani hidup saya sesuai dengan keyakinan agamawi saya | ____ |
| --- | --- |
| 1. Tidak masalah apa yang saya percayai selama saya baik | ____ |
| 1. Saya sering mengalami perasaan kuat adanya kehadiran ilahi | ____ |
| 1. Seluruh pendekatan saya pada hidup didasarkan pada agama saya | ____ |
| 1. Do’a yang saya panjatkan ketika sendirian sama pentingnya dengan yang saya panjatkan ketika beribadah berjamaah di rumah ibadah (contoh: masjid, gereja, pura, wihara, dll). | ____ |
| 1. Dalam seminggu, saya datang ke rumah ibadah sekali atau bisa lebih | ____ |
| 1. Agama saya penting karena menjawab banyak pertanyaan mengenai makna kehidupan | ____ |
| 1. Saya senang membaca bacaan mengenai agama saya. | ____ |
| 1. Sangat penting bagi saya untuk menghabiskan waktu dalam pemikiran dan Do’a pribadi. | ____ |
| 1. Bagi saya, agama terutama memberikan kenyamanan ketika ada masalah dan ketika mengalami kesedihan. | ____ |
| 1. Doa adalah untuk kedamaian dan kebahagiaan. | ____ |
| 1. Saya berdoa terutama untuk mendapatkan kelegaan dan perlindungan | ____ |
| 1. Saya datang ke rumah ibadah karena membantu saya mendapat teman. | ____ |
| 1. Saya datang ke rumah ibadah terutama karena saya senang menemui orang-orang yang saya kenal disana | ____ |
| 1. Saya datang ke rumah ibadah seringkali untuk menghabiskan waktu bersama teman-teman saya. | ____ |

## **The Ethical Position Questionnaire (EPQ)**

Berikan angka 1 (satu) sampai dengan 9 (sembilan) untuk menunjukkan setuju atau tidaknya anda terhadap suatu pernyataan menurut jenjang di bawah ini:

| 1 | 2 | 3 | 4 | 5 | 6 | 7 | 8 | 9 |
| --- | --- | --- | --- | --- | --- | --- | --- | --- |
| Sepenuhnya tidak setuju | Tidak setuju | Agak tidak setuju | Sedikit tidak setuju | Ragu-ragu | Sedikit setuju | Agak setuju | Setuju | Sepenuhnya setuju |

**Catatan**: Semakin tinggi angka yang anda bubuhkan berarti semakin setuju anda dengan pernyataan itu.

| 1. Orang-orang baiknya dapat memastikan bahwa tindakan mereka tidak pernah ditujukan untuk menyakiti orang lain, sekecil apapun itu. | ____ |
| --- | --- |
| 1. Sekecil apapun, membawa resiko kepada orang lain sebaiknya tidak boleh ditolerir. | ____ |
| 1. Apapun manfaat yang bisa didapat, adanya kemungkinan menyakiti orang lain itu selalu salah. | ____ |
| 1. Baik secara psikologis maupun fisik, seseorang seharusnya tidak menyakiti orang lain. | ____ |
| 1. Seseorang seharusnya tidak melakukan tindakan apapun yang berkemungkinan mengancam martabat dan kesejahteraan orang lain. | ____ |
| 1. Jika bisa merugikan orang yang tidak bersalah, maka sebaiknya tindakan itu tidak dilakukan. | ____ |
| 1. Memutuskan melakukan tindakan dengan pertimbangan menyeimbangkan akibat positif dan negatif dari tindakan adalah tidak bermoral. | ____ |
| 1. Martabat dan kesejahteraan orang banyak harus menjadi perhatian yang paling penting di masyarakat manapun. | ____ |
| 1. Mengorbankan kesejahteraan orang lain tidak pernah diperlukan | ____ |
| 1. Perilaku moral adalah berbagai tindakan yang sangat sesuai dengan cita-cita dari hampir semua tindakan "sempurna". | ____ |
| 1. Tiada prinsip etika sebegitu pentingnya sehingga harus dijadikan komponen kode etik apapun. | ____ |
| 1. Apa yang etis dan tidak di suatu situasi dan masyarakat bisa berbeda di situasi dan masyarakat lain. | ____ |
| 1. Standar moral harus dilihat sebagai sesuatu yang khas; Apa yang seseorang anggap sebagai bermoral boleh jadi dianggap tidak bermoral oleh orang lain. | ____ |
| 1. Jenis moralitas yang berbeda tidak dapat diperbandingkan kebenarannya. | ____ |
| 1. Apa yang etis untuk semua orang tidak akan pernah bisa terjawab karena pertimbangan apa yang bermoral dari tidak bermoral itu terserah kepada perseorangan. | ____ |
| 1. Standar moral hanyalah peraturan pribadi yang menunjukkan bagaimana seseorang harus bersikap, dan tidak dapat diterapkan dalam membuat penilaian terhadap orang lain. | ____ |
| 1. Pertimbangan etis dalam hubungan antar pribadi sangat rumit sehingga individu harus diijinkan untuk merumuskan kode mereka sendiri. | ____ |
| 1. Menerjemahkan posisi etis secara kaku sehingga mencegah beberapa jenis tindakan dapat menghalangi hubungan dan penyesuaian antar sesama yang lebih baik. | ____ |
| 1. Tidak ada aturan tentang kebohongan yang bisa dirumuskan; apakah kebohongan diperbolehkan atau tidak itu benar-benar tergantung pada situasi. | ____ |
| 1. Apakah kebohongan dinilai sebagai moral atau tidak bermoral tergantung pada situasi dan kondisi yang mendasari tindakan tersebut. | ____ |
