# Supplementary material for: Do religious beliefs influence concerns for animal welfare? the role of religious orientation and ethical ideologies in attitudes toward animal protection amongst Muslim teachers and school staff in East Java, Indonesia
Source: PLoS One. 2021 Jul 16;16(7):e0254880. doi: 10.1371/journal.pone.0254880 (PMC8284611; doi:10.1371/journal.pone.0254880)
Supplement: S1 Data — (DOCX) [file pone.0254880.s003.docx]

Contents

[Data screening 3](#_Toc76689382)

[1. Dropped and missing 3](#_Toc76689383)

[2. Data Reduction 5](#_Toc76689384)

[A. Normal distribution check 5](#_Toc76689385)

[B. Factor Analysis 9](#_Toc76689386)

[AIS (Animal Issue Scale) 9](#_Toc76689387)

[ROS (Religious Orientation Scale) 15](#_Toc76689388)

[EPQ (Ethical position questionairres) 19](#_Toc76689389)

[AAS (Animal attitude scale) 23](#_Toc76689390)

[3. Normal distribution transformation analysis 34](#_Toc76689391)

[References 35](#_Toc76689392)

List of Tables

[Table 1 Missing case analysis 3](#_Toc76689222)

[Table 2 Normal distribution check 5](#_Toc76689223)

[Table 3 AIS total variance explained 9](#_Toc76689224)

[Table 4 AIS Rotated factor Matrix 10](#_Toc76689225)

[Table 5 All AIS’s Alpha 11](#_Toc76689226)

[Table 6 All AIS item-total statistics 11](#_Toc76689227)

[Table 7 AIS-Animuse subscale’s alpha 12](#_Toc76689228)

[Table 8 AIS-Animuse subscale item-total statistics 12](#_Toc76689229)

[Table 9 AIS-Intgity subscale’s alpha 12](#_Toc76689230)

[Table 10 AIS-intgity subscale item-total statistics 13](#_Toc76689231)

[Table 11 AIS-KillWelfare subscale’s alpha 13](#_Toc76689232)

[Table 12 AIS-KillWelfare subscale item-total statistics 13](#_Toc76689233)

[Table 13 AIS-Xprmnt subscale’s alpha 13](#_Toc76689234)

[Table 14 AIS-Xprmnt subscale item-total statistics 14](#_Toc76689235)

[Table 15 AIS-Genchng subscale’s alpha 14](#_Toc76689236)

[Table 16 AIS-Genchng subscale item-total statistics 14](#_Toc76689237)

[Table 17 AIS-EnvIss subscale’s alpha 14](#_Toc76689238)

[Table 18 AIS-EnvIss subscale’s item-total statistics 14](#_Toc76689239)

[Table 19 AIS-SocAtt subscale’s alpha 15](#_Toc76689240)

[Table 20 AIS-SocAtt subscale’s item-total statistics 15](#_Toc76689241)

[Table 21 ROS total variance explained 16](#_Toc76689242)

[Table 22 ROS Rotated factor Matrix 17](#_Toc76689243)

[Table 23 ROS IP subscale’s alpha 17](#_Toc76689244)

[Table 24 ROS IP subscale’s item-total statistics 17](#_Toc76689245)

[Table 25 ROS-ES subscale’s alpha 18](#_Toc76689246)

[Table 26 ROS-ES subscale’s item-total statistics 18](#_Toc76689247)

[Table 27 EPQ total variance explained 19](#_Toc76689248)

[Table 28 EPQ Pattern Matrix 20](#_Toc76689249)

[Table 29 EPQ total variance explained 21](#_Toc76689250)

[Table 30 EPQ pattern matrix 22](#_Toc76689251)

[Table 31 EPQ-Idealism subscale’s alpha 22](#_Toc76689252)

[Table 32 EPQ-Idealims subscale item-total statistics 22](#_Toc76689253)

[Table 33 EPQ-Relativism subscale’s alpha 23](#_Toc76689254)

[Table 34 EPQ-Relativism subscale item-total statistics 23](#_Toc76689255)

[Table 35 1st Run-AAS total variance explained 24](#_Toc76689256)

[Table 36 1st Run-AAS Pattern Matrix 26](#_Toc76689257)

[Table 37 1st Run-AAS factor correlation matrix 26](#_Toc76689258)

[Table 38 2nd Run-AAS total variance explained 28](#_Toc76689259)

[Table 39 2nd Run-AAS Pattern Matrix 29](#_Toc76689260)

[Table 40 2nd Run-AAS factor correlation matrix 29](#_Toc76689261)

[Table 41 3rd Run-AAS total variance explained 30](#_Toc76689262)

[Table 42 3rd Run-AAS Pattern Matrix 31](#_Toc76689263)

[Table 43 3rd Run-AAS factor correlation matrix 31](#_Toc76689264)

[Table 44 4th (Final) Run-AAS total variance explained 32](#_Toc76689265)

[Table 45 4th (Final) Run-AAS pattern matrix 33](#_Toc76689266)

[Table 46 4th (Final) Run-AAS factor correaltion matrix 33](#_Toc76689267)

[Table 47 AAS factor 1 subscale’s correlation 33](#_Toc76689268)

[Table 48 AAS factor 2 subscale’s alpha 34](#_Toc76689269)

[Table 49 AAS factor 3 subscale’s alpha 34](#_Toc76689270)

[Table 50 All scales distribution descriptive statistics 34](#_Toc76689271)

# Data screening

## Dropped and missing

- Total N = 1007,
- Dropped = 78 participants (subjects removed because they didn’t answers more than two questionnaire scales (approximately 35-40 items are blanks), or when they fill one same answer in most of the questions)
- Remaining 929 participants

Table 1 Missing case analysis

| **Univariate Statistics** | | | | | | | |
| --- | --- | --- | --- | --- | --- | --- | --- |
|  | N | Mean | Std. Deviation | Missing | | No. of Extremes^a^ | |
|  |  |  |  | Count | Percent | Low | High |
| AAS01 | 926 | 3.5551 | 1.23055 | 3 | .3 | 73 | 0 |
| AAS02 | 923 | 2.7833 | .90471 | 6 | .6 | 0 | 31 |
| AAS03 | 927 | 3.9202 | .85321 | 2 | .2 | 52 | 0 |
| AAS04 | 927 | 3.8716 | .95680 | 2 | .2 | 23 | 0 |
| AAS05 | 926 | 3.1058 | 1.04784 | 3 | .3 | 47 | 0 |
| AAS06 | 922 | 2.8449 | .88957 | 7 | .8 | 45 | 35 |
| AAS07 | 923 | 2.9079 | .93101 | 6 | .6 | 52 | 43 |
| AAS08 | 924 | 2.1407 | .79660 | 5 | .5 | 0 | 59 |
| AAS09 | 926 | 2.3272 | .89434 | 3 | .3 | 0 | 14 |
| AAS10 | 926 | 4.1080 | .85816 | 3 | .3 | 52 | 0 |
| AAS11 | 927 | 3.3236 | .94841 | 2 | .2 | 28 | 0 |
| AAS12 | 925 | 3.1211 | 1.01527 | 4 | .4 | 44 | 0 |
| AAS13 | 926 | 2.8974 | .88913 | 3 | .3 | 42 | 24 |
| AAS14 | 925 | 3.0195 | .99981 | 4 | .4 | 40 | 0 |
| AAS15 | 926 | 3.0140 | .96692 | 3 | .3 | 38 | 57 |
| AAS16 | 927 | 2.4746 | .89222 | 2 | .2 | 0 | 21 |
| AAS17 | 926 | 3.3391 | .89896 | 3 | .3 | 31 | 0 |
| AAS18 | 926 | 2.9633 | .97911 | 3 | .3 | 42 | 41 |
| AAS19 | 926 | 3.3099 | .96537 | 3 | .3 | 33 | 0 |
| AAS20 | 924 | 3.7695 | 1.04082 | 5 | .5 | 43 | 0 |
| ROS01 | 922 | 4.3026 | .72085 | 7 | .8 | 20 | 0 |
| ROS02 | 924 | 3.6688 | .98783 | 5 | .5 | 31 | 0 |
| ROS03 | 925 | 4.1708 | .81849 | 4 | .4 | 28 | 0 |
| ROS04 | 928 | 4.2360 | .74269 | 1 | .1 | 19 | 0 |
| ROS05 | 926 | 4.1803 | .82454 | 3 | .3 | 43 | 0 |
| ROS06 | 924 | 3.8074 | .96735 | 5 | .5 | 25 | 0 |
| ROS07 | 928 | 4.3858 | .70893 | 1 | .1 | 16 | 0 |
| ROS08 | 928 | 4.2813 | .70565 | 1 | .1 | 18 | 0 |

Table 1 Missing case analysis (continued)

|  | N | Mean | Std. Deviation | Missing | | No. of Extremes^a^ | |
| --- | --- | --- | --- | --- | --- | --- | --- |
|  |  |  |  | Count | Percent | Low | High |
| ROS09 | 928 | 4.0162 | .88177 | 1 | .1 | 59 | 0 |
| ROS10 | 926 | 4.2970 | .83913 | 3 | .3 | 42 | 0 |
| ROS11 | 928 | 4.4537 | .66505 | 1 | .1 | 61 | 0 |
| ROS12 | 923 | 4.2741 | .78741 | 6 | .6 | 27 | 0 |
| ROS13 | 926 | 2.8942 | 1.12640 | 3 | .3 | 0 | 0 |
| ROS14 | 926 | 2.8780 | 1.11171 | 3 | .3 | 0 | 0 |
| ROS15 | 923 | 2.5915 | 1.10167 | 6 | .6 | 0 | 54 |
| AI01 | 929 | 3.0463 | 1.03755 | 0 | .0 | 0 | 0 |
| AI02 | 928 | 3.5075 | .78273 | 1 | .1 | 14 | 0 |
| AI03 | 924 | 3.7121 | .77153 | 5 | .5 | 55 | 0 |
| AI04 | 929 | 2.9193 | .94512 | 0 | .0 | 68 | 18 |
| AI05 | 929 | 2.9505 | .92948 | 0 | .0 | 60 | 22 |
| AI06 | 929 | 3.7524 | .91859 | 0 | .0 | 25 | 0 |
| AI07 | 928 | 2.7845 | 1.01494 | 1 | .1 | 0 | 26 |
| AI08 | 923 | 2.4020 | .99340 | 6 | .6 | 0 | 28 |
| AI09 | 928 | 2.3179 | 1.05267 | 1 | .1 | 0 | 25 |
| AI10 | 928 | 2.3631 | .97577 | 1 | .1 | 0 | 18 |
| AI11 | 928 | 2.4192 | 1.05508 | 1 | .1 | 0 | 23 |
| AI12 | 929 | 1.9160 | .95109 | 0 | .0 | 0 | 76 |
| AI13 | 928 | 2.6875 | 1.13841 | 1 | .1 | 0 | 47 |
| AI14 | 928 | 2.3244 | 1.11544 | 1 | .1 | 0 | 31 |
| AI15 | 929 | 2.5113 | 1.15040 | 0 | .0 | 0 | 30 |
| AI16 | 928 | 2.0991 | 1.04116 | 1 | .1 | 0 | 21 |
| AI17 | 929 | 1.9634 | .95070 | 0 | .0 | 0 | 83 |
| AI18 | 928 | 1.9892 | .97426 | 1 | .1 | 0 | 95 |
| AI19 | 928 | 1.9106 | .97409 | 1 | .1 | 0 | 87 |
| AI20 | 929 | 2.1076 | .98219 | 0 | .0 | 0 | 20 |
| AI21 | 925 | 2.2616 | 1.03755 | 4 | .4 | 0 | 21 |
| AI22 | 928 | 3.0550 | .98105 | 1 | .1 | 76 | 0 |
| AI23 | 929 | 3.4909 | .92042 | 0 | .0 | 36 | 0 |
| AI24 | 928 | 3.2209 | .93575 | 1 | .1 | 55 | 0 |
| AI25 | 927 | 2.7487 | 1.00991 | 2 | .2 | 0 | 31 |
| AI26 | 929 | 3.0388 | .97413 | 0 | .0 | 73 | 35 |
| AI27 | 929 | 3.5145 | .95933 | 0 | .0 | 41 | 0 |
| AI28 | 927 | 3.3107 | .97624 | 2 | .2 | 43 | 0 |

Table 1 Missing case analysis (continued)

|  | N | Mean | Std. Deviation | Missing | | No. of Extremes^a^ | |
| --- | --- | --- | --- | --- | --- | --- | --- |
|  |  |  |  | Count | Percent | Low | High |
| AI29 | 929 | 3.2605 | .92438 | 0 | .0 | 36 | 0 |
| AI30 | 929 | 3.1346 | .93321 | 0 | .0 | 45 | 0 |
| AI31 | 929 | 3.2540 | .93659 | 0 | .0 | 43 | 0 |
| AI32 | 928 | 2.1455 | 1.01412 | 1 | .1 | 0 | 22 |
| AI33 | 928 | 3.1153 | 1.15127 | 1 | .1 | 0 | 0 |
| AI34 | 928 | 2.2802 | 1.05161 | 1 | .1 | 0 | 28 |
| AI35 | 928 | 2.8847 | 1.02222 | 1 | .1 | 0 | 31 |
| AI36 | 929 | 2.0205 | 1.05077 | 0 | .0 | 0 | 21 |
| AI37 | 927 | 2.2902 | 1.06735 | 2 | .2 | 0 | 19 |
| AI38 | 927 | 2.6170 | 1.11975 | 2 | .2 | 0 | 36 |
| AI39 | 901 | 2.4018 | 1.00695 | 28 | 3.0 | 0 | 11 |
| AI40 | 899 | 2.2225 | .94592 | 30 | 3.2 | 0 | 11 |
| AI41 | 898 | 1.8385 | .96516 | 31 | 3.3 | 0 | 79 |
| AI42 | 899 | 1.9155 | .98518 | 30 | 3.2 | 0 | 78 |
| AI43 | 893 | 2.1624 | 1.04895 | 36 | 3.9 | 0 | 22 |
| EPQ01 | 920 | 7.6935 | 1.52654 | 9 | 1.0 | 51 | 0 |
| EPQ02 | 918 | 6.9935 | 1.89356 | 11 | 1.2 | 78 | 0 |
| EPQ03 | 919 | 7.2840 | 1.78253 | 10 | 1.1 | 58 | 0 |
| EPQ04 | 917 | 7.9160 | 1.42288 | 12 | 1.3 | 82 | 0 |
| EPQ05 | 920 | 7.8565 | 1.50337 | 9 | 1.0 | 52 | 0 |
| EPQ06 | 920 | 7.8326 | 1.46568 | 9 | 1.0 | 48 | 0 |
| EPQ07 | 919 | 5.8629 | 2.58509 | 10 | 1.1 | 0 | 0 |
| EPQ08 | 920 | 7.5663 | 1.55633 | 9 | 1.0 | 58 | 0 |
| EPQ09 | 917 | 5.9106 | 2.67543 | 12 | 1.3 | 0 | 0 |
| EPQ10 | 919 | 6.9750 | 1.75840 | 10 | 1.1 | 53 | 0 |
| EPQ11 | 918 | 5.9804 | 2.36930 | 11 | 1.2 | 31 | 0 |
| EPQ12 | 920 | 6.9478 | 1.76519 | 9 | 1.0 | 52 | 0 |
| EPQ13 | 920 | 6.6946 | 1.95130 | 9 | 1.0 | 63 | 0 |
| EPQ14 | 917 | 6.6150 | 1.91897 | 12 | 1.3 | 53 | 0 |
| EPQ15 | 917 | 6.1439 | 2.25188 | 12 | 1.3 | 17 | 0 |
| EPQ16 | 895 | 6.3106 | 2.23298 | 34 | 3.7 | 19 | 0 |
| EPQ17 | 911 | 6.1273 | 2.18266 | 18 | 1.9 | 11 | 0 |
| EPQ18 | 910 | 6.3308 | 2.08020 | 19 | 2.0 | 83 | 0 |
| EPQ19 | 912 | 5.8662 | 2.40327 | 17 | 1.8 | 36 | 0 |
| EPQ20 | 900 | 6.0433 | 2.37181 | 29 | 3.1 | 40 | 0 |
| a. Number of cases outside the range (Mean - 2*SD, Mean + 2*SD). | | | | | | | |

## Data Reduction

### Normal distribution check

Using skewness and kurtosis value for sample more than 300, the present study remove items with kurtosis value outside the range between -7 to 7, or skew value outside the range between -2 to 2 [1]. Thus, EPQ item number 1, 4, 5 and 6 are removed.

Table 2 Normal distribution check

| **Descriptive Statistics** | | | | | | | | | |
| --- | --- | --- | --- | --- | --- | --- | --- | --- | --- |
|  | N | Minimum | Maximum | Mean | SD | Skewness | | Kurtosis | |
|  | Statistic | Statistic | Statistic | Statistic | Statistic | Statistic | Std. Error | Statistic | Std. Error |
| AAS01 | 929 | 1 | 5 | 3.55 | 1.229 | -.627 | .080 | -.653 | .160 |
| AAS02 | 929 | 1 | 5 | 2.78 | .902 | .246 | .080 | -.162 | .160 |
| AAS03 | 929 | 1 | 5 | 3.92 | .852 | -.956 | .080 | 1.477 | .160 |
| AAS04 | 929 | 1 | 5 | 3.87 | .956 | -.943 | .080 | .738 | .160 |
| AAS05 | 929 | 1 | 5 | 3.11 | 1.046 | .002 | .080 | -.709 | .160 |
| AAS06 | 929 | 1 | 5 | 2.85 | .886 | .216 | .080 | -.035 | .160 |
| AAS07 | 929 | 1 | 5 | 2.91 | .928 | .111 | .080 | -.178 | .160 |
| AAS08 | 929 | 1 | 5 | 2.14 | .794 | .969 | .080 | 1.642 | .160 |
| AAS09 | 929 | 1 | 5 | 2.33 | .893 | .692 | .080 | .259 | .160 |
| AAS10 | 929 | 1 | 5 | 4.11 | .857 | -.992 | .080 | 1.011 | .160 |
| AAS11 | 929 | 1 | 5 | 3.32 | .947 | -.213 | .080 | -.277 | .160 |
| AAS12 | 929 | 1 | 5 | 3.12 | 1.013 | -.095 | .080 | -.672 | .160 |
| AAS13 | 929 | 1 | 5 | 2.90 | .888 | .036 | .080 | -.396 | .160 |
| AAS14 | 929 | 1 | 5 | 3.02 | .998 | .130 | .080 | -.618 | .160 |
| AAS15 | 929 | 1 | 5 | 3.01 | .965 | .109 | .080 | -.513 | .160 |
| AAS16 | 929 | 1 | 5 | 2.47 | .891 | .493 | .080 | .182 | .160 |
| AAS17 | 929 | 1 | 5 | 3.34 | .898 | -.407 | .080 | .099 | .160 |
| AAS18 | 929 | 1 | 5 | 2.96 | .978 | .067 | .080 | -.770 | .160 |
| AAS19 | 929 | 1 | 5 | 3.31 | .964 | -.336 | .080 | -.357 | .160 |
| AAS20 | 929 | 1 | 5 | 3.77 | 1.038 | -.934 | .080 | .523 | .160 |
| ROS01 | 929 | 1 | 5 | 4.30 | .718 | -1.033 | .080 | 1.645 | .160 |
| ROS02 | 929 | 1 | 5 | 3.67 | .985 | -.788 | .080 | .281 | .160 |
| ROS03 | 929 | 1 | 5 | 4.17 | .817 | -1.050 | .080 | 1.608 | .160 |
| ROS04 | 929 | 1 | 5 | 4.24 | .742 | -.901 | .080 | 1.203 | .160 |
| ROS05 | 929 | 1 | 5 | 4.18 | .823 | -1.159 | .080 | 1.768 | .160 |
| ROS06 | 929 | 1 | 5 | 3.81 | .965 | -.840 | .080 | .519 | .160 |
| ROS07 | 929 | 1 | 5 | 4.39 | .709 | -1.331 | .080 | 2.997 | .160 |
| ROS08 | 929 | 1 | 5 | 4.28 | .705 | -.957 | .080 | 1.620 | .160 |

Table 2 Normal distribution check (continued)

|  | N | Minimum | Maximum | Mean | SD | Skewness | | Kurtosis | |
| --- | --- | --- | --- | --- | --- | --- | --- | --- | --- |
|  | Statistic | Statistic | Statistic | Statistic | Statistic | Statistic | Std. Error | Statistic | Std. Error |
| ROS09 | 929 | 1 | 5 | 4.02 | .881 | -.931 | .080 | .920 | .160 |
| ROS10 | 929 | 1 | 5 | 4.30 | .838 | -1.365 | .080 | 2.072 | .160 |
| ROS11 | 929 | 1 | 5 | 4.45 | .665 | -1.241 | .080 | 2.369 | .160 |
| ROS12 | 929 | 1 | 5 | 4.27 | .785 | -1.250 | .080 | 2.294 | .160 |
| ROS13 | 929 | 1 | 5 | 2.89 | 1.125 | .136 | .080 | -.777 | .160 |
| ROS14 | 929 | 1 | 5 | 2.88 | 1.110 | .129 | .080 | -.775 | .160 |
| ROS15 | 929 | 1 | 5 | 2.59 | 1.098 | .466 | .080 | -.527 | .160 |
| AI01_AnimUse | 929 | 1 | 5 | 3.05 | 1.038 | -.209 | .080 | -.633 | .160 |
| AI02_AnimUse | 929 | 1 | 5 | 3.51 | .782 | -.471 | .080 | .580 | .160 |
| AI03_AnimUse | 929 | 1 | 5 | 3.71 | .769 | -.534 | .080 | .601 | .160 |
| AI04_AnimUse | 929 | 1 | 5 | 2.92 | .945 | -.222 | .080 | -.592 | .160 |
| AI05_AnimUse | 929 | 1 | 5 | 2.95 | .929 | -.208 | .080 | -.473 | .160 |
| AI06_Intgrty | 929 | 1 | 5 | 3.75 | .919 | -.903 | .080 | .851 | .160 |
| AI07_Intgrty | 929 | 1 | 5 | 2.78 | 1.014 | .050 | .080 | -.806 | .160 |
| AI08_Intgrty | 929 | 1 | 5 | 2.40 | .990 | .574 | .080 | -.077 | .160 |
| AI09_Intgrty | 929 | 1 | 5 | 2.32 | 1.052 | .567 | .080 | -.387 | .160 |
| AI10_Intgrty | 929 | 1 | 5 | 2.36 | .975 | .478 | .080 | -.270 | .160 |
| AI11_Intgrty | 929 | 1 | 5 | 2.42 | 1.055 | .437 | .080 | -.608 | .160 |
| AI12_Kill | 929 | 1 | 5 | 1.92 | .951 | 1.283 | .080 | 1.637 | .160 |
| AI13_Kill | 929 | 1 | 5 | 2.69 | 1.138 | .110 | .080 | -.859 | .160 |
| AI14_Kill | 929 | 1 | 5 | 2.32 | 1.115 | .509 | .080 | -.608 | .160 |
| AI15_Kill | 929 | 1 | 5 | 2.51 | 1.150 | .232 | .080 | -.996 | .160 |
| AI16_Kill | 929 | 1 | 5 | 2.10 | 1.041 | .836 | .080 | .017 | .160 |
| AI17_Welfare | 929 | 1 | 5 | 1.96 | .951 | 1.174 | .080 | 1.273 | .160 |
| AI18_Welfare | 929 | 1 | 5 | 1.99 | .974 | 1.109 | .080 | .923 | .160 |
| AI19_Welfare | 929 | 1 | 5 | 1.91 | .974 | 1.170 | .080 | 1.006 | .160 |
| AI20_Welfare | 929 | 1 | 5 | 2.11 | .982 | .891 | .080 | .406 | .160 |
| AI21_Welfare | 929 | 1 | 5 | 2.26 | 1.035 | .644 | .080 | -.280 | .160 |
| AI22_Xprmnt | 929 | 1 | 5 | 3.05 | .981 | -.392 | .080 | -.327 | .160 |
| AI23_Xprmnt | 929 | 1 | 5 | 3.49 | .920 | -.800 | .080 | .444 | .160 |
| AI24_Xprmnt | 929 | 1 | 5 | 3.22 | .935 | -.540 | .080 | .061 | .160 |
| AI25_Xprmnt | 929 | 1 | 5 | 2.75 | 1.009 | .065 | .080 | -.589 | .160 |
| AI26_Xprmnt | 929 | 1 | 5 | 3.04 | .974 | -.344 | .080 | -.341 | .160 |
| AI27_Genchng | 929 | 1 | 5 | 3.51 | .959 | -.698 | .080 | .357 | .160 |
| AI28_Genchng | 929 | 1 | 5 | 3.31 | .975 | -.389 | .080 | -.185 | .160 |
| AI29_Genchng | 929 | 1 | 5 | 3.26 | .924 | -.326 | .080 | -.109 | .160 |

Table 2 Normal distribution check (continued)

|  | N | Minimum | Maximum | Mean | SD | Skewness | | Kurtosis | |
| --- | --- | --- | --- | --- | --- | --- | --- | --- | --- |
|  | Statistic | Statistic | Statistic | Statistic | Statistic | Statistic | Std. Error | Statistic | Std. Error |
| AI30_Genchng | 929 | 1 | 5 | 3.13 | .933 | -.239 | .080 | -.205 | .160 |
| AI31_Genchng | 929 | 1 | 5 | 3.25 | .937 | -.446 | .080 | -.117 | .160 |
| AI32_EnvIss | 929 | 1 | 5 | 2.15 | 1.014 | .844 | .080 | .178 | .160 |
| AI33_EnvIss | 929 | 1 | 5 | 3.12 | 1.151 | -.209 | .080 | -.852 | .160 |
| AI34_EnvIss | 929 | 1 | 5 | 2.28 | 1.051 | .683 | .080 | -.192 | .160 |
| AI35_EnvIss | 929 | 1 | 5 | 2.88 | 1.022 | -.126 | .080 | -.722 | .160 |
| AI36_EnvIss | 929 | 1 | 5 | 2.02 | 1.051 | .964 | .080 | .195 | .160 |
| AI37_EnvIss | 929 | 1 | 5 | 2.29 | 1.066 | .486 | .080 | -.616 | .160 |
| AI38_SocAtt | 929 | 1 | 5 | 2.62 | 1.119 | .143 | .080 | -.865 | .160 |
| AI39_SocAtt | 929 | 1 | 5 | 2.40 | .992 | .196 | .080 | -.681 | .160 |
| AI40_SocAtt | 929 | 1 | 5 | 2.22 | .931 | .497 | .080 | -.151 | .160 |
| AI41_SocAtt | 929 | 1 | 5 | 1.84 | .949 | 1.178 | .080 | .915 | .160 |
| AI42_SocAtt | 929 | 1 | 5 | 1.92 | .970 | 1.189 | .080 | 1.199 | .160 |
| AI43_SocAtt | 929 | 1 | 5 | 2.16 | 1.028 | .718 | .080 | -.074 | .160 |
| EPQ01_Ideal_removed_skewkurtos | 929 | 1 | 9 | 7.69 | 1.519 | -2.102 | .080 | 4.542 | .160 |
| EPQ02_Ideal | 929 | 1 | 9 | 6.99 | 1.882 | -1.445 | .080 | 1.279 | .160 |
| EPQ03_Ideal | 929 | 1 | 9 | 7.28 | 1.773 | -1.702 | .080 | 2.362 | .160 |
| EPQ04_Ideal_removed_skewkurtos | 929 | 1 | 9 | 7.92 | 1.414 | -2.286 | .080 | 5.767 | .160 |
| EPQ05_Ideal_removed_skewkurtos | 929 | 1 | 9 | 7.86 | 1.496 | -2.288 | .080 | 5.516 | .160 |
| EPQ06_Ideal_removed_skewkurtos | 929 | 1 | 9 | 7.83 | 1.459 | -2.343 | .080 | 6.097 | .160 |
| EPQ07_Ideal | 929 | 1 | 9 | 5.86 | 2.571 | -.575 | .080 | -1.113 | .160 |
| EPQ08_Ideal | 929 | 1 | 9 | 7.57 | 1.549 | -1.901 | .080 | 3.582 | .160 |
| EPQ09_Ideal | 929 | 1 | 9 | 5.91 | 2.658 | -.584 | .080 | -1.194 | .160 |
| EPQ10_Ideal | 929 | 1 | 9 | 6.97 | 1.749 | -1.269 | .080 | 1.038 | .160 |

Table 2 Normal distribution check (continued)

|  | N | Minimum | Maximum | Mean | SD | Skewness | | Kurtosis | |
| --- | --- | --- | --- | --- | --- | --- | --- | --- | --- |
|  | Statistic | Statistic | Statistic | Statistic | Statistic | Statistic | Std. Error | Statistic | Std. Error |
| EPQ11_Relative | 929 | 1 | 9 | 5.98 | 2.355 | -.656 | .080 | -.823 | .160 |
| EPQ12_Relative | 929 | 1 | 9 | 6.95 | 1.757 | -1.304 | .080 | 1.098 | .160 |
| EPQ13_Relative | 929 | 1 | 9 | 6.70 | 1.942 | -1.220 | .080 | .629 | .160 |
| EPQ14_Relative | 929 | 1 | 9 | 6.62 | 1.907 | -.993 | .080 | .129 | .160 |
| EPQ15_Relative | 929 | 1 | 9 | 6.14 | 2.237 | -.780 | .080 | -.606 | .160 |
| EPQ16_Relative | 929 | 1 | 9 | 6.32 | 2.192 | -.947 | .080 | -.296 | .160 |
| EPQ17_Relative | 929 | 1 | 9 | 6.13 | 2.162 | -.742 | .080 | -.550 | .160 |
| EPQ18_Relative | 929 | 1 | 9 | 6.33 | 2.059 | -.935 | .080 | -.057 | .160 |
| EPQ19_Relative | 929 | 1 | 9 | 5.87 | 2.381 | -.638 | .080 | -.901 | .160 |
| EPQ20_Relative | 929 | 1 | 9 | 6.05 | 2.335 | -.779 | .080 | -.617 | .160 |
| Valid N (listwise) | 929 |  |  |  |  |  |  |  |  |

### Factor Analysis

#### AIS (Animal Issue Scale)

Table 3 AIS total variance explained

| **Total Variance Explained** | | | | | | | | | |
| --- | --- | --- | --- | --- | --- | --- | --- | --- | --- |
| Factor | Initial Eigenvalues | | | Extraction Sums of Squared Loadings | | | Rotation Sums of Squared Loadings | | |
|  | Total | % of Variance | Cumulative % | Total | % of Variance | Cumulative % | Total | % of Variance | Cumulative % |
| 1 | 9.131 | 29.454 | 29.454 | 8.684 | 28.012 | 28.012 | 4.295 | 13.854 | 13.854 |
| 2 | 3.472 | 11.202 | 40.656 | 3.019 | 9.738 | 37.750 | 2.355 | 7.596 | 21.450 |
| 3 | 1.647 | 5.313 | 45.968 | 1.109 | 3.578 | 41.328 | 2.293 | 7.398 | 28.848 |
| 4 | 1.397 | 4.508 | 50.476 | .965 | 3.114 | 44.442 | 1.967 | 6.344 | 35.192 |
| 5 | 1.267 | 4.088 | 54.564 | .820 | 2.646 | 47.088 | 1.792 | 5.779 | 40.971 |
| 6 | 1.203 | 3.881 | 58.445 | .712 | 2.296 | 49.384 | 1.639 | 5.287 | 46.258 |
| 7 | 1.135 | 3.661 | 62.106 | .622 | 2.007 | 51.392 | 1.591 | 5.134 | 51.392 |
| 8 | .895 | 2.886 | 64.992 |  |  |  |  |  |  |
| 9 | .828 | 2.672 | 67.664 |  |  |  |  |  |  |
| 10 | .779 | 2.513 | 70.176 |  |  |  |  |  |  |
| 11 | .718 | 2.316 | 72.492 |  |  |  |  |  |  |
| 12 | .682 | 2.199 | 74.691 |  |  |  |  |  |  |
| 13 | .667 | 2.152 | 76.844 |  |  |  |  |  |  |
| 14 | .630 | 2.034 | 78.877 |  |  |  |  |  |  |
| 15 | .594 | 1.916 | 80.794 |  |  |  |  |  |  |
| 16 | .543 | 1.753 | 82.547 |  |  |  |  |  |  |

Table 3 AIS total variance explained (continued)

| **Total Variance Explained** | | | | | | | | | |
| --- | --- | --- | --- | --- | --- | --- | --- | --- | --- |
| Factor | Initial Eigenvalues | | | Extraction Sums of Squared Loadings | | | Rotation Sums of Squared Loadings | | |
|  | Total | % of Variance | Cumulative % | Total | % of Variance | Cumulative % | Total | % of Variance | Cumulative % |
| 17 | .520 | 1.678 | 84.225 |  |  |  |  |  |  |
| 18 | .493 | 1.592 | 85.817 |  |  |  |  |  |  |
| 19 | .471 | 1.520 | 87.337 |  |  |  |  |  |  |
| 20 | .444 | 1.432 | 88.769 |  |  |  |  |  |  |
| 21 | .440 | 1.419 | 90.187 |  |  |  |  |  |  |
| 22 | .403 | 1.300 | 91.487 |  |  |  |  |  |  |
| 23 | .376 | 1.214 | 92.701 |  |  |  |  |  |  |
| 24 | .366 | 1.179 | 93.881 |  |  |  |  |  |  |
| 25 | .340 | 1.096 | 94.976 |  |  |  |  |  |  |
| 26 | .308 | .994 | 95.971 |  |  |  |  |  |  |
| 27 | .299 | .964 | 96.935 |  |  |  |  |  |  |
| 28 | .281 | .907 | 97.842 |  |  |  |  |  |  |
|  |  |  |  |  |  |  |  |  |  |
| 29 | .260 | .840 | 98.682 |  |  |  |  |  |  |
| 30 | .222 | .715 | 99.397 |  |  |  |  |  |  |
| 31 | .187 | .603 | 100.000 |  |  |  |  |  |  |
| Extraction Method: Principal Axis Factoring. | | | | | | | | | |

Table 4 AIS Rotated factor Matrix

| **Rotated Factor Matrix^a^** | | | | | | | |
| --- | --- | --- | --- | --- | --- | --- | --- |
|  | Factor | | | | | | |
|  | 1 | 2 | 3 | 4 | 5 | 6 | 7 |
| AI01_AnimUse |  |  |  |  |  |  | .490 |
| AI02_AnimUse |  |  |  |  |  |  | .447 |
| AI04_AnimUse |  |  |  |  |  |  | .624 |
| AI05_AnimUse |  |  |  |  |  |  | .654 |
| AI08_Intgrty |  |  |  | .542 |  |  |  |
| AI09_Intgrty |  |  |  | .662 |  |  |  |
| AI10_Intgrty |  |  |  | .589 |  |  |  |
| AI11_Intgrty |  |  |  | .557 |  |  |  |
| AI14_Kill | .439 |  |  |  |  |  |  |
| AI16_Kill | .556 |  |  |  |  |  |  |
| AI17_Welfare | .768 |  |  |  |  |  |  |
| AI18_Welfare | .765 |  |  |  |  |  |  |

Table 4 AIS Rotated factor Matrix

| **Rotated Factor Matrix^a^** | | | | | | | |
| --- | --- | --- | --- | --- | --- | --- | --- |
|  | Factor | | | | | | |
|  | 1 | 2 | 3 | 4 | 5 | 6 | 7 |
| AI19_Welfare | .798 |  |  |  |  |  |  |
| AI20_Welfare | .701 |  |  |  |  |  |  |
| AI21_Welfare | .501 |  |  |  |  |  |  |
| AI24_Xprmnt |  |  |  |  | .553 |  |  |
| AI25_Xprmnt |  |  |  |  | .636 |  |  |
| AI26_Xprmnt |  |  |  |  | .755 |  |  |
| AI27_Genchng |  |  | .633 |  |  |  |  |
| AI28_Genchng |  |  | .693 |  |  |  |  |
| AI29_Genchng |  |  | .749 |  |  |  |  |
| AI30_Genchng |  |  | .600 |  |  |  |  |
| AI34_EnvIss |  |  |  |  |  | .542 |  |
| AI35_EnvIss |  |  |  |  |  | .439 |  |
| AI36_EnvIss |  |  |  |  |  | .596 |  |
| AI37_EnvIss |  |  |  |  |  | .465 |  |
| AI39_SocAtt |  | .606 |  |  |  |  |  |
| AI40_SocAtt |  | .765 |  |  |  |  |  |
| AI41_SocAtt |  | .591 |  |  |  |  |  |
| AI42_SocAtt |  | .570 |  |  |  |  |  |
| AI43_SocAtt |  | .435 |  |  |  |  |  |
| Extraction Method: Principal Axis Factoring.  Rotation Method: Varimax with Kaiser Normalization. | | | | | | | |
| a. Rotation converged in 7 iterations. | | | | | | | |

AI01_AnimUse,AI02_AnimUse,AI04_AnimUse,AI05_AnimUse,AI08_Intgrty,AI09_Intgrty,AI10_Intgrty,AI11_Intgrty,AI14_Kill,AI16_Kill,AI17_Welfare,AI18_Welfare,AI19_Welfare,AI20_Welfare,AI21_Welfare,AI24_Xprmnt,AI25_Xprmnt,AI26_Xprmnt,AI27_Genchng,AI28_Genchng,AI29_Genchng,AI30_Genchng,AI34_EnvIss,AI35_EnvIss,AI36_EnvIss,AI37_EnvIss,AI39_SocAtt,AI40_SocAtt,AI41_SocAtt,AI42_SocAtt,AI43_SocAtt

##### AIS all items Cronbach’s alpha reliability

Reliability

Table 5 All AIS’s Alpha

| **Reliability Statistics** | |
| --- | --- |
| Cronbach's Alpha | N of Items |
| .914 | 31 |

Table 6 All AIS item-total statistics

| **Item-Total Statistics** | | | | |
| --- | --- | --- | --- | --- |
|  | Scale Mean if Item Deleted | Scale Variance if Item Deleted | Corrected Item-Total Correlation | Cronbach's Alpha if Item Deleted |
| AI01_AnimUse | 75.79 | 252.283 | .269 | .915 |
| AI02_AnimUse | 75.33 | 257.046 | .183 | .915 |
| AI04_AnimUse | 75.91 | 252.278 | .302 | .914 |
| AI05_AnimUse | 75.88 | 252.320 | .307 | .914 |
| AI08_Intgrty | 76.43 | 246.951 | .460 | .912 |
| AI09_Intgrty | 76.52 | 243.662 | .532 | .910 |
| AI10_Intgrty | 76.47 | 243.378 | .589 | .910 |
| AI11_Intgrty | 76.41 | 243.979 | .520 | .911 |
| AI14_Kill | 76.51 | 246.030 | .428 | .912 |
| AI16_Kill | 76.73 | 241.155 | .619 | .909 |
| AI17_Welfare | 76.87 | 242.251 | .645 | .909 |
| AI18_Welfare | 76.84 | 241.954 | .638 | .909 |
| AI19_Welfare | 76.92 | 243.231 | .595 | .910 |
| AI20_Welfare | 76.73 | 242.417 | .616 | .909 |
| AI21_Welfare | 76.57 | 243.931 | .533 | .910 |
| AI24_Xprmnt | 75.61 | 248.558 | .434 | .912 |
| AI25_Xprmnt | 76.08 | 243.652 | .558 | .910 |
| AI26_Xprmnt | 75.79 | 246.656 | .478 | .911 |
| AI27_Genchng | 75.32 | 251.937 | .308 | .914 |
| AI28_Genchng | 75.52 | 249.944 | .368 | .913 |
| AI29_Genchng | 75.57 | 251.312 | .344 | .913 |
| AI30_Genchng | 75.70 | 250.208 | .378 | .913 |
| AI34_EnvIss | 76.55 | 243.937 | .524 | .911 |
| AI35_EnvIss | 75.95 | 247.616 | .422 | .912 |
| AI36_EnvIss | 76.81 | 243.146 | .549 | .910 |
| AI37_EnvIss | 76.54 | 242.671 | .555 | .910 |
| AI39_SocAtt | 76.43 | 246.664 | .468 | .911 |
| AI40_SocAtt | 76.61 | 245.025 | .561 | .910 |
| AI41_SocAtt | 76.99 | 244.344 | .573 | .910 |
| AI42_SocAtt | 76.92 | 244.273 | .562 | .910 |
| AI43_SocAtt | 76.67 | 242.387 | .587 | .910 |

##### AIS-Use of Animal subscale (Animuse) Cronbach’s alpha reliability

Table 7 AIS-Animuse subscale’s alpha

| **Reliability Statistics** | |
| --- | --- |
| Cronbach's Alpha | N of Items |
| .664 | 4 |

Table 8 AIS-Animuse subscale item-total statistics

| **Item-Total Statistics** | | | | |
| --- | --- | --- | --- | --- |
|  | Scale Mean if Item Deleted | Scale Variance if Item Deleted | Corrected Item-Total Correlation | Cronbach's Alpha if Item Deleted |
| AI01_AnimUse | 9.38 | 4.030 | .421 | .618 |
| AI02_AnimUse | 8.92 | 4.920 | .382 | .636 |
| AI04_AnimUse | 9.50 | 4.043 | .506 | .553 |
| AI05_AnimUse | 9.47 | 4.172 | .480 | .572 |

##### AIS Animal integrity destruction (Intgrty)

Table 9 AIS-Intgity subscale’s alpha

| **Reliability Statistics** | |
| --- | --- |
| Cronbach's Alpha | N of Items |
| .782 | 4 |

Table 10 AIS-intgity subscale item-total statistics

| **Item-Total Statistics** | | | | |
| --- | --- | --- | --- | --- |
|  | Scale Mean if Item Deleted | Scale Variance if Item Deleted | Corrected Item-Total Correlation | Cronbach's Alpha if Item Deleted |
| AI08_Intgrty | 7.10 | 6.367 | .540 | .753 |
| AI09_Intgrty | 7.18 | 5.764 | .628 | .708 |
| AI10_Intgrty | 7.14 | 6.100 | .621 | .713 |
| AI11_Intgrty | 7.08 | 6.008 | .566 | .741 |

##### AIS-Animal killing and welfare destruction (KillWelfare) Cronbach’s alpha reliability

Table 11 AIS-KillWelfare subscale’s alpha

| **Reliability Statistics** | |
| --- | --- |
| Cronbach's Alpha | N of Items |
| .875 | 7 |

Table 12 AIS-KillWelfare subscale item-total statistics

| **Item-Total Statistics** | | | | |
| --- | --- | --- | --- | --- |
|  | Scale Mean if Item Deleted | Scale Variance if Item Deleted | Corrected Item-Total Correlation | Cronbach's Alpha if Item Deleted |
| AI14_Kill | 12.33 | 22.449 | .473 | .884 |
| AI16_Kill | 12.56 | 21.584 | .623 | .862 |
| AI17_Welfare | 12.69 | 20.995 | .779 | .842 |
| AI18_Welfare | 12.67 | 21.036 | .750 | .846 |
| AI19_Welfare | 12.75 | 21.029 | .751 | .845 |
| AI20_Welfare | 12.55 | 21.237 | .716 | .850 |
| AI21_Welfare | 12.39 | 22.269 | .547 | .872 |

##### AIS-Animal experimentation (Xprmnt) Cronbach’s alpha reliability

Table 13 AIS-Xprmnt subscale’s alpha

| **Reliability Statistics** | |
| --- | --- |
| Cronbach's Alpha | N of Items |
| .821 | 3 |

Table 14 AIS-Xprmnt subscale item-total statistics

| **Item-Total Statistics** | | | | |
| --- | --- | --- | --- | --- |
|  | Scale Mean if Item Deleted | Scale Variance if Item Deleted | Corrected Item-Total Correlation | Cronbach's Alpha if Item Deleted |
| AI24_Xprmnt | 5.79 | 3.253 | .638 | .791 |
| AI25_Xprmnt | 6.26 | 2.899 | .688 | .742 |
| AI26_Xprmnt | 5.97 | 2.970 | .703 | .725 |

##### AIS-Animal genetic change (Genchng) Cronbach’s alpha reliability

Table 15 AIS-Genchng subscale’s alpha

| **Reliability Statistics** | |
| --- | --- |
| Cronbach's Alpha | N of Items |
| .798 | 4 |

Table 16 AIS-Genchng subscale item-total statistics

| **Item-Total Statistics** | | | | |
| --- | --- | --- | --- | --- |
|  | Scale Mean if Item Deleted | Scale Variance if Item Deleted | Corrected Item-Total Correlation | Cronbach's Alpha if Item Deleted |
| AI27_Genchng | 9.71 | 5.348 | .605 | .749 |
| AI28_Genchng | 9.91 | 5.089 | .662 | .720 |
| AI29_Genchng | 9.96 | 5.357 | .641 | .732 |
| AI30_Genchng | 10.09 | 5.708 | .532 | .784 |

##### AIS-Harming animals for environmental issue (Enviss) Cronbach’s alpha reliability

Table 17 AIS-EnvIss subscale’s alpha

| **Reliability Statistics** | |
| --- | --- |
| Cronbach's Alpha | N of Items |
| .747 | 4 |

Table 18 AIS-EnvIss subscale’s item-total statistics

| **Item-Total Statistics** | | | | |
| --- | --- | --- | --- | --- |
|  | Scale Mean if Item Deleted | Scale Variance if Item Deleted | Corrected Item-Total Correlation | Cronbach's Alpha if Item Deleted |
| AI34_EnvIss | 7.20 | 5.947 | .573 | .672 |
| AI35_EnvIss | 6.59 | 6.743 | .415 | .756 |
| AI36_EnvIss | 7.46 | 5.819 | .605 | .653 |
| AI37_EnvIss | 7.19 | 5.859 | .580 | .667 |

##### AIS-Harm animals for social issue (SocAtt) Cronbach’s alpha reliability

Table 19 AIS-SocAtt subscale’s alpha

| **Reliability Statistics** | |
| --- | --- |
| Cronbach's Alpha | N of Items |
| .840 | 5 |

Table 20 AIS-SocAtt subscale’s item-total statistics

| **Item-Total Statistics** | | | | |
| --- | --- | --- | --- | --- |
|  | Scale Mean if Item Deleted | Scale Variance if Item Deleted | Corrected Item-Total Correlation | Cronbach's Alpha if Item Deleted |
| AI39_SocAtt | 8.14 | 10.037 | .549 | .833 |
| AI40_SocAtt | 8.32 | 9.515 | .712 | .789 |
| AI41_SocAtt | 8.71 | 9.427 | .711 | .789 |
| AI42_SocAtt | 8.63 | 9.434 | .688 | .795 |
| AI43_SocAtt | 8.38 | 9.747 | .571 | .828 |

#### ROS (Religious Orientation Scale)

Table 21 ROS total variance explained

| **Total Variance Explained** | | | | | | | | | |
| --- | --- | --- | --- | --- | --- | --- | --- | --- | --- |
| Factor | Initial Eigenvalues | | | Extraction Sums of Squared Loadings | | | Rotation Sums of Squared Loadings | | |
|  | Total | % of Variance | Cumulative % | Total | % of Variance | Cumulative % | Total | % of Variance | Cumulative % |
| 1 | 5.251 | 37.504 | 37.504 | 4.716 | 33.687 | 33.687 | 4.713 | 33.666 | 33.666 |
| 2 | 2.471 | 17.652 | 55.156 | 2.172 | 15.513 | 49.200 | 2.175 | 15.534 | 49.200 |
| 3 | .999 | 7.133 | 62.289 |  |  |  |  |  |  |
| 4 | .843 | 6.023 | 68.312 |  |  |  |  |  |  |
| 5 | .777 | 5.548 | 73.860 |  |  |  |  |  |  |
| 6 | .625 | 4.463 | 78.323 |  |  |  |  |  |  |
| 7 | .563 | 4.019 | 82.342 |  |  |  |  |  |  |
| 8 | .463 | 3.309 | 85.651 |  |  |  |  |  |  |
| 9 | .426 | 3.042 | 88.693 |  |  |  |  |  |  |
| 10 | .416 | 2.971 | 91.664 |  |  |  |  |  |  |
| 11 | .360 | 2.574 | 94.239 |  |  |  |  |  |  |
| 12 | .305 | 2.177 | 96.416 |  |  |  |  |  |  |
| 13 | .271 | 1.939 | 98.354 |  |  |  |  |  |  |
| 14 | .230 | 1.646 | 100.000 |  |  |  |  |  |  |
| Extraction Method: Principal Axis Factoring. | | | | | | | | | |

Table 22 ROS Rotated factor Matrix

| **Rotated Factor Matrix^a^** | | |
| --- | --- | --- |
|  | Factor | |
|  | 1 | 2 |
| ROS01 | .673 |  |
| ROS03 | .608 |  |
| ROS04 | .705 |  |
| ROS05 | .577 |  |
| ROS06 | .358 |  |
| ROS07 | .741 |  |
| ROS08 | .750 |  |
| ROS09 | .630 |  |
| ROS10 | .665 |  |
| ROS11 | .764 |  |
| ROS12 | .622 |  |
| ROS13 |  | .833 |
| ROS14 |  | .894 |
| ROS15 |  | .787 |
| Extraction Method: Principal Axis Factoring.  Rotation Method: Varimax with Kaiser Normalization. | | |
| a. Rotation converged in 3 iterations. | | |

##### ROS Intrinsic Personal (IP) Cronbach’s alpha reliability

Table 23 ROS IP subscale’s alpha

| **Reliability Statistics** | |
| --- | --- |
| Cronbach's Alpha | N of Items |
| .879 | 11 |

Table 24 ROS IP subscale’s item-total statistics

| **Item-Total Statistics** | | | | |
| --- | --- | --- | --- | --- |
|  | Scale Mean if Item Deleted | Scale Variance if Item Deleted | Corrected Item-Total Correlation | Cronbach's Alpha if Item Deleted |
| ROS01 | 42.10 | 28.860 | .622 | .866 |
| ROS03 | 42.23 | 28.628 | .558 | .870 |

Table 24 ROS IP subscale’s item-total statistics

| **Item-Total Statistics** | | | | |
| --- | --- | --- | --- | --- |
|  | Scale Mean if Item Deleted | Scale Variance if Item Deleted | Corrected Item-Total Correlation | Cronbach's Alpha if Item Deleted |
| ROS04 | 42.17 | 28.378 | .663 | .863 |
| ROS05 | 42.22 | 28.686 | .546 | .871 |
| ROS06 | 42.60 | 29.699 | .337 | .889 |
| ROS07 | 42.02 | 28.475 | .687 | .862 |
| ROS08 | 42.12 | 28.392 | .703 | .861 |
| ROS09 | 42.39 | 27.877 | .593 | .868 |
| ROS10 | 42.11 | 28.057 | .610 | .867 |
| ROS11 | 41.95 | 28.690 | .708 | .862 |
| ROS12 | 42.13 | 28.712 | .576 | .869 |

##### ROS Extrinsic Personal (ES) Cronbach’s alpha reliability

Table 25 ROS-ES subscale’s alpha

| **Reliability Statistics** | |
| --- | --- |
| Cronbach's Alpha | N of Items |
| .875 | 3 |

Table 26 ROS-ES subscale’s item-total statistics

| **Item-Total Statistics** | | | | |
| --- | --- | --- | --- | --- |
|  | Scale Mean if Item Deleted | Scale Variance if Item Deleted | Corrected Item-Total Correlation | Cronbach's Alpha if Item Deleted |
| ROS13 | 5.47 | 4.174 | .751 | .832 |
| ROS14 | 5.49 | 4.054 | .807 | .781 |
| ROS15 | 5.77 | 4.365 | .724 | .856 |

#### EPQ (Ethical position questionairres)

##### Factor analysis: Eigenvalue >= 1

Table 27 EPQ total variance explained

| **Total Variance Explained** | | | | | | | |
| --- | --- | --- | --- | --- | --- | --- | --- |
| Factor | Initial Eigenvalues | | | Extraction Sums of Squared Loadings | | | Rotation Sums of Squared Loadings^a^ |
|  | Total | % of Variance | Cumulative % | Total | % of Variance | Cumulative % | Total |
| 1 | 3.712 | 33.748 | 33.748 | 3.193 | 29.031 | 29.031 | 2.703 |
| 2 | 1.580 | 14.365 | 48.113 | 1.031 | 9.375 | 38.407 | 1.951 |
| 3 | 1.172 | 10.657 | 58.769 | .708 | 6.434 | 44.841 | 2.184 |
| 4 | .891 | 8.103 | 66.872 |  |  |  |  |
| 5 | .714 | 6.491 | 73.363 |  |  |  |  |
| 6 | .634 | 5.760 | 79.123 |  |  |  |  |
| 7 | .609 | 5.538 | 84.661 |  |  |  |  |
| 8 | .528 | 4.804 | 89.466 |  |  |  |  |
| 9 | .444 | 4.036 | 93.501 |  |  |  |  |
| 10 | .374 | 3.401 | 96.902 |  |  |  |  |
| 11 | .341 | 3.098 | 100.000 |  |  |  |  |
| Extraction Method: Principal Axis Factoring. | | | | | | | |
| a. When factors are correlated, sums of squared loadings cannot be added to obtain a total variance. | | | | | | | |

Table 28 EPQ Pattern Matrix

| **Pattern Matrix^a^** | | | |
| --- | --- | --- | --- |
|  | Factor | | |
|  | 1 | 2 | 3 |
| EPQ02_Ideal |  | .570 |  |
| EPQ03_Ideal |  | .680 |  |
| EPQ08_Ideal |  | .563 |  |
| EPQ10_Ideal |  | .453 |  |
| EPQ13_Relative | .742 |  |  |
| EPQ14_Relative | .679 |  |  |
| EPQ15_Relative | .757 |  |  |
| EPQ16_Relative | .508 |  |  |
| EPQ18_Relative |  |  | .396 |
| EPQ19_Relative |  |  | .882 |
| EPQ20_Relative |  |  | .727 |
| Extraction Method: Principal Axis Factoring.  Rotation Method: Oblimin with Kaiser Normalization. | | | |
| a. Rotation converged in 7 iterations. | | | |

##### Factor analysis: Forced to two-factor

Table 29 EPQ total variance explained

| **Total Variance Explained** | | | | | | | |
| --- | --- | --- | --- | --- | --- | --- | --- |
| Factor | Initial Eigenvalues | | | Extraction Sums of Squared Loadings | | | Rotation Sums of Squared Loadings^a^ |
|  | Total | % of Variance | Cumulative % | Total | % of Variance | Cumulative % | Total |
| 1 | 3.712 | 33.748 | 33.748 | 3.094 | 28.129 | 28.129 | 2.805 |
| 2 | 1.580 | 14.365 | 48.113 | .935 | 8.500 | 36.629 | 2.025 |
| 3 | 1.172 | 10.657 | 58.769 |  |  |  |  |
| 4 | .891 | 8.103 | 66.872 |  |  |  |  |
| 5 | .714 | 6.491 | 73.363 |  |  |  |  |
| 6 | .634 | 5.760 | 79.123 |  |  |  |  |
| 7 | .609 | 5.538 | 84.661 |  |  |  |  |
| 8 | .528 | 4.804 | 89.466 |  |  |  |  |
| 9 | .444 | 4.036 | 93.501 |  |  |  |  |
| 10 | .374 | 3.401 | 96.902 |  |  |  |  |
| 11 | .341 | 3.098 | 100.000 |  |  |  |  |
| Extraction Method: Principal Axis Factoring. | | | | | | | |
| a. When factors are correlated, sums of squared loadings cannot be added to obtain a total variance. | | | | | | | |

Table 30 EPQ pattern matrix

| **Pattern Matrix^a^** | | |
| --- | --- | --- |
|  | Factor | |
|  | 1 | 2 |
| EPQ02_Ideal |  | .520 |
| EPQ03_Ideal |  | .627 |
| EPQ08_Ideal |  | .584 |
| EPQ10_Ideal |  | .480 |
| EPQ13_Relative | .459 |  |
| EPQ14_Relative | .491 |  |
| EPQ15_Relative | .624 |  |
| EPQ16_Relative | .534 |  |
| EPQ18_Relative | .528 |  |
| EPQ19_Relative | .729 |  |
| EPQ20_Relative | .673 |  |
| Extraction Method: Principal Axis Factoring.  Rotation Method: Oblimin with Kaiser Normalization. | | |
| a. Rotation converged in 5 iterations. | | |

###### EPQ Idealism subscale Cronbach’s alpha reliability

Table 31 EPQ-Idealism subscale’s alpha

| **Reliability Statistics** | |
| --- | --- |
| Cronbach's Alpha | N of Items |
| .656 | 4 |

Table 32 EPQ-Idealims subscale item-total statistics

| **Item-Total Statistics** | | | | |
| --- | --- | --- | --- | --- |
|  | Scale Mean if Item Deleted | Scale Variance if Item Deleted | Corrected Item-Total Correlation | Cronbach's Alpha if Item Deleted |
| EPQ02_Ideal | 21.83 | 14.389 | .419 | .603 |
| EPQ03_Ideal | 21.53 | 14.090 | .502 | .542 |
| EPQ08_Ideal | 21.25 | 15.833 | .461 | .577 |
| EPQ10_Ideal | 21.84 | 15.654 | .376 | .629 |

###### EPQ Relativism subscale Cronbach’s alpha reliability

Table 33 EPQ-Relativism subscale’s alpha

| **Reliability Statistics** | |
| --- | --- |
| Cronbach's Alpha | N of Items |
| .803 | 7 |

Table 34 EPQ-Relativism subscale item-total statistics

| **Item-Total Statistics** | | | | |
| --- | --- | --- | --- | --- |
|  | Scale Mean if Item Deleted | Scale Variance if Item Deleted | Corrected Item-Total Correlation | Cronbach's Alpha if Item Deleted |
| EPQ13_Relative | 37.32 | 82.090 | .528 | .779 |
| EPQ14_Relative | 37.40 | 81.733 | .553 | .775 |
| EPQ15_Relative | 37.87 | 76.119 | .597 | .765 |
| EPQ16_Relative | 37.70 | 79.851 | .505 | .783 |
| EPQ18_Relative | 37.68 | 81.396 | .506 | .782 |
| EPQ19_Relative | 38.15 | 76.198 | .543 | .776 |
| EPQ20_Relative | 37.97 | 77.393 | .526 | .779 |

#### AAS (Animal attitude scale)

##### Factor Analysis

###### 1^st^ Run

Table 35 1st Run-AAS total variance explained

| Factor | Initial Eigenvalues | | | Extraction Sums of Squared Loadings | | | Rotation Sums of Squared Loadings^a^ |
| --- | --- | --- | --- | --- | --- | --- | --- |
|  | Total | % of Variance | Cumulative % | Total | % of Variance | Cumulative % | Total |
| 1 | 3.557 | 17.787 | 17.787 | 2.877 | 14.383 | 14.383 | 1.507 |
| 2 | 2.193 | 10.964 | 28.751 | 1.538 | 7.691 | 22.074 | 1.739 |
| 3 | 1.515 | 7.574 | 36.326 | .858 | 4.289 | 26.363 | 1.641 |
| 4 | 1.118 | 5.589 | 41.915 | .488 | 2.439 | 28.802 | 1.651 |
| 5 | 1.057 | 5.286 | 47.201 | .390 | 1.949 | 30.751 | 1.835 |
| 6 | .988 | 4.939 | 52.140 |  |  |  |  |
| 7 | .935 | 4.673 | 56.813 |  |  |  |  |
| 8 | .837 | 4.186 | 60.998 |  |  |  |  |
| 9 | .819 | 4.096 | 65.094 |  |  |  |  |
| 10 | .778 | 3.889 | 68.983 |  |  |  |  |
| 11 | .763 | 3.813 | 72.796 |  |  |  |  |
| 12 | .732 | 3.659 | 76.456 |  |  |  |  |
| 13 | .714 | 3.570 | 80.025 |  |  |  |  |
| 14 | .677 | 3.386 | 83.412 |  |  |  |  |
| 15 | .629 | 3.147 | 86.559 |  |  |  |  |
| 16 | .610 | 3.048 | 89.607 |  |  |  |  |
| 17 | .576 | 2.878 | 92.485 |  |  |  |  |
| 18 | .543 | 2.717 | 95.202 |  |  |  |  |
| 19 | .492 | 2.462 | 97.664 |  |  |  |  |
| 20 | .467 | 2.336 | 100.000 |  |  |  |  |
| Extraction Method: Principal Axis Factoring. | | | | | | | |
| a. When factors are correlated, sums of squared loadings cannot be added to obtain a total variance. | | | | | | | |

Table 36 1st Run-AAS Pattern Matrix

| **Pattern Matrix^a^** | | | | | |
| --- | --- | --- | --- | --- | --- |
|  | Factor | | | | |
|  | 1 | 2 | 3 | 4 | 5 |
| AAS02 | .537 |  |  |  |  |
| AAS05 |  |  |  |  |  |
| AAS14 |  |  |  |  |  |
| AAS06 |  |  |  |  |  |
| AAS10 |  | .542 |  |  |  |
| AAS04 |  | .530 |  |  |  |
| AAS03 |  | .470 |  |  |  |
| AAS20 |  |  |  |  |  |
| AAS01 |  |  |  |  |  |
| AAS11 |  |  |  |  |  |
| AAS12 |  |  | -.491 |  |  |
| AAS07 |  |  | .480 |  |  |
| AAS19 |  |  | .443 |  |  |
| AAS13 |  |  | -.419 |  |  |
| AAS18 |  |  |  |  |  |
| AAS08 |  |  |  | -.821 |  |
| AAS09 |  |  |  | -.518 |  |
| AAS15 |  |  |  |  | .694 |
| AAS16 |  |  |  |  |  |
| AAS17 |  |  |  |  |  |
| Extraction Method: Principal Axis Factoring.  Rotation Method: Oblimin with Kaiser Normalization.^a^ | | | | | |
| a. Rotation converged in 19 iterations. | | | | | |

Table 37 1st Run-AAS factor correlation matrix

| **Factor Correlation Matrix** | | | | | |
| --- | --- | --- | --- | --- | --- |
| Factor | 1 | 2 | 3 | 4 | 5 |
| 1 | 1.000 | .035 | -.221 | -.288 | .351 |
| 2 | .035 | 1.000 | .111 | .296 | -.209 |
| 3 | -.221 | .111 | 1.000 | .074 | -.364 |
| 4 | -.288 | .296 | .074 | 1.000 | -.283 |
| 5 | .351 | -.209 | -.364 | -.283 | 1.000 |
| Extraction Method: Principal Axis Factoring.  Rotation Method: Oblimin with Kaiser Normalization. | | | | | |

###### 2^nd^ Run

Table 38 2nd Run-AAS total variance explained

| **Total Variance Explained** | | | | | | | |
| --- | --- | --- | --- | --- | --- | --- | --- |
| Factor | Initial Eigenvalues | | | Extraction Sums of Squared Loadings | | | Rotation Sums of Squared Loadings^a^ |
|  | Total | % of Variance | Cumulative % | Total | % of Variance | Cumulative % | Total |
| 1 | 2.398 | 19.987 | 19.987 | 1.748 | 14.570 | 14.570 | 1.401 |
| 2 | 1.859 | 15.492 | 35.479 | 1.234 | 10.283 | 24.853 | 1.107 |
| 3 | 1.282 | 10.681 | 46.160 | .609 | 5.074 | 29.927 | 1.234 |
| 4 | 1.010 | 8.421 | 54.580 | .318 | 2.646 | 32.573 | 1.064 |
| 5 | .893 | 7.443 | 62.023 |  |  |  |  |
| 6 | .816 | 6.798 | 68.822 |  |  |  |  |
| 7 | .766 | 6.386 | 75.208 |  |  |  |  |
| 8 | .703 | 5.855 | 81.063 |  |  |  |  |
| 9 | .652 | 5.435 | 86.499 |  |  |  |  |
| 10 | .603 | 5.022 | 91.521 |  |  |  |  |
| 11 | .530 | 4.416 | 95.936 |  |  |  |  |
| 12 | .488 | 4.064 | 100.000 |  |  |  |  |
| Extraction Method: Principal Axis Factoring. | | | | | | | |
| a. When factors are correlated, sums of squared loadings cannot be added to obtain a total variance. | | | | | | | |

Table 39 2nd Run-AAS Pattern Matrix

| **Pattern Matrix^a^** | | | | |
| --- | --- | --- | --- | --- |
|  | Factor | | | |
|  | 1 | 2 | 3 | 4 |
| AAS14 | .669 |  |  |  |
| AAS02 | .432 |  |  |  |
| AAS15 | .400 |  |  |  |
| AAS04 |  | .584 |  |  |
| AAS03 |  | .504 |  |  |
| AAS10 |  | .471 |  |  |
| AAS08 |  |  | .735 |  |
| AAS09 |  |  | .579 |  |
| AAS07 |  |  |  | .469 |
| AAS12 |  |  |  | -.437 |
| AAS19 |  |  |  |  |
| AAS13 |  |  |  |  |
| Extraction Method: Principal Axis Factoring.  Rotation Method: Oblimin with Kaiser Normalization.^a^ | | | | |
| a. Rotation converged in 10 iterations. | | | | |

Table 40 2nd Run-AAS factor correlation matrix

| **Factor Correlation Matrix** | | | | |
| --- | --- | --- | --- | --- |
| Factor | 1 | 2 | 3 | 4 |
| 1 | 1.000 | .018 | .316 | -.386 |
| 2 | .018 | 1.000 | -.293 | .083 |
| 3 | .316 | -.293 | 1.000 | -.057 |
| 4 | -.386 | .083 | -.057 | 1.000 |
| Extraction Method: Principal Axis Factoring.  Rotation Method: Oblimin with Kaiser Normalization. | | | | |

###### 3^rd^ Run

Table 41 3rd Run-AAS total variance explained

| **Total Variance Explained** | | | | | | | |
| --- | --- | --- | --- | --- | --- | --- | --- |
| Factor | Initial Eigenvalues | | | Extraction Sums of Squared Loadings | | | Rotation Sums of Squared Loadings^a^ |
|  | Total | % of Variance | Cumulative % | Total | % of Variance | Cumulative % | Total |
| 1 | 2.160 | 21.602 | 21.602 | 1.508 | 15.082 | 15.082 | 1.179 |
| 2 | 1.711 | 17.113 | 38.714 | 1.032 | 10.323 | 25.405 | 1.145 |
| 3 | 1.133 | 11.329 | 50.043 | .487 | 4.866 | 30.270 | 1.095 |
| 4 | .990 | 9.903 | 59.947 |  |  |  |  |
| 5 | .840 | 8.396 | 68.343 |  |  |  |  |
| 6 | .744 | 7.440 | 75.783 |  |  |  |  |
| 7 | .684 | 6.842 | 82.624 |  |  |  |  |
| 8 | .607 | 6.073 | 88.697 |  |  |  |  |
| 9 | .603 | 6.028 | 94.725 |  |  |  |  |
| 10 | .528 | 5.275 | 100.000 |  |  |  |  |
| Extraction Method: Principal Axis Factoring. | | | | | | | |
| a. When factors are correlated, sums of squared loadings cannot be added to obtain a total variance. | | | | | | | |

Table 42 3rd Run-AAS Pattern Matrix

| **Pattern Matrix^a^** | | | |
| --- | --- | --- | --- |
|  | Factor | | |
|  | 1 | 2 | 3 |
| AAS09 | .692 |  |  |
| AAS08 | .600 |  |  |
| AAS14 |  | .639 |  |
| AAS12 |  | .492 |  |
| AAS15 |  | .455 |  |
| AAS02 |  |  |  |
| AAS07 |  |  |  |
| AAS04 |  |  | .597 |
| AAS03 |  |  | .515 |
| AAS10 |  |  | .495 |
| Extraction Method: Principal Axis Factoring.  Rotation Method: Oblimin with Kaiser Normalization.^a^ | | | |
| a. Rotation converged in 8 iterations. | | | |

Table 43 3rd Run-AAS factor correlation matrix

| **Factor Correlation Matrix** | | | |
| --- | --- | --- | --- |
| Factor | 1 | 2 | 3 |
| 1 | 1.000 | .227 | -.312 |
| 2 | .227 | 1.000 | -.068 |
| 3 | -.312 | -.068 | 1.000 |
| Extraction Method: Principal Axis Factoring.  Rotation Method: Oblimin with Kaiser Normalization. | | | |

###### 4^th^ (Final) Run

Table 44 4th (Final) Run-AAS total variance explained

| **Total Variance Explained** | | | | | | | |
| --- | --- | --- | --- | --- | --- | --- | --- |
| Factor | Initial Eigenvalues | | | Extraction Sums of Squared Loadings | | | Rotation Sums of Squared Loadings^a^ |
|  | Total | % of Variance | Cumulative % | Total | % of Variance | Cumulative % | Total |
| 1 | 1.969 | 24.617 | 24.617 | 1.355 | 16.932 | 16.932 | 1.132 |
| 2 | 1.633 | 20.412 | 45.030 | .960 | 11.998 | 28.930 | .949 |
| 3 | 1.077 | 13.468 | 58.498 | .442 | 5.523 | 34.453 | 1.046 |
| 4 | .814 | 10.176 | 68.674 |  |  |  |  |
| 5 | .695 | 8.688 | 77.362 |  |  |  |  |
| 6 | .646 | 8.071 | 85.433 |  |  |  |  |
| 7 | .619 | 7.741 | 93.173 |  |  |  |  |
| 8 | .546 | 6.827 | 100.000 |  |  |  |  |
| Extraction Method: Principal Axis Factoring. | | | | | | | |
| a. When factors are correlated, sums of squared loadings cannot be added to obtain a total variance. | | | | | | | |

Table 45 4th (Final) Run-AAS pattern matrix

| **Pattern Matrix^a^** | | | |
| --- | --- | --- | --- |
|  | Factor | | |
|  | 1 | 2 | 3 |
| AAS08 | .655 |  |  |
| AAS09 | .648 |  |  |
| AAS14 |  | .651 |  |
| AAS15 |  | .501 |  |
| AAS12 |  | .427 |  |
| AAS04 |  |  | .587 |
| AAS03 |  |  | .526 |
| AAS10 |  |  | .463 |
| Extraction Method: Principal Axis Factoring.  Rotation Method: Oblimin with Kaiser Normalization.^a^ | | | |
| a. Rotation converged in 9 iterations. | | | |

Table 46 4th (Final) Run-AAS factor correaltion matrix

| **Factor Correlation Matrix** | | | |
| --- | --- | --- | --- |
| Factor | 1 | 2 | 3 |
| 1 | 1.000 | .215 | -.338 |
| 2 | .215 | 1.000 | .071 |
| 3 | -.338 | .071 | 1.000 |
| Extraction Method: Principal Axis Factoring.  Rotation Method: Oblimin with Kaiser Normalization. | | | |

##### Reliability Analysis Model 4 (4^th^ run)

###### AAS Factor 1 (AAS08 & 09)

Table 47 AAS factor 1 subscale’s correlation

| **Correlations** | | | |
| --- | --- | --- | --- |
|  | | AAS08 | AAS09 |
| AAS08 | Pearson Correlation | 1 | .432^**^ |
|  | Sig. (2-tailed) |  | .000 |
|  | N | 929 | 929 |
| AAS09 | Pearson Correlation | .432^**^ | 1 |
|  | Sig. (2-tailed) | .000 |  |
|  | N | 929 | 929 |
| **. Correlation is significant at the 0.01 level (2-tailed). | | | |

###### AAS F2 (AAS14, 15, 12)

Table 48 AAS factor 2 subscale’s alpha

| **Reliability Statistics** | |
| --- | --- |
| Cronbach's Alpha | N of Items |
| .528 | 3 |

###### AAS F3 (AAS04, 03, 10)

Table 49 AAS factor 3 subscale’s alpha

| **Reliability Statistics** | |
| --- | --- |
| Cronbach's Alpha | N of Items |
| .559 | 3 |

Decision: Delete AAS from analysis.

## Normal distribution transformation analysis

Previous analysis used the formula kurtosis value / kurtosis std. error and skewness value /skewness std. error, resulting the log-10 or square root of several scales that are found outside the boundary of -2.58 to +2.58 (LOS 0.01).

However, Kim [1] stressed out the tendency of large samples producing inflated z considering large samples will usually produce a very small standard error for both skewness and kurtosis. Therefore, using skewness and kurtosis references value for sample more than 300, the present study remove items with kurtosis value outside the range between -7 to 7, or skew value outside the range between -2 to 2 [1]. Below table showed that all scales are safely within the normal distribution bound, and therefore, the present study doesn’t apply any transformation.

Table 50 All scales distribution descriptive statistics

| **Descriptive Statistics** | | | | | |
| --- | --- | --- | --- | --- | --- |
|  | N | Skewness | | Kurtosis | |
|  | Statistic | Statistic | Std. Error | Statistic | Std. Error |
| AAS_F1 | 929 | .617 | .080 | .788 | .160 |
| AAS_F2 | 929 | -.128 | .080 | .060 | .160 |
| AAS_F3 | 929 | -.634 | .080 | .886 | .160 |
| AIS_ALL | 929 | .389 | .080 | .939 | .160 |
| AIS_AnimUse | 929 | -.132 | .080 | .191 | .160 |
| AIS_Intgrty | 929 | .446 | .080 | .239 | .160 |
| AIS_KillWelfare | 929 | .789 | .080 | .689 | .160 |

Table 50 All scales distribution descriptive statistics

| **Descriptive Statistics** | | | | | |
| --- | --- | --- | --- | --- | --- |
|  | N | Skewness | | Kurtosis | |
|  | Statistic | Statistic | Std. Error | Statistic | Std. Error |
| AIS_Xprmnt | 929 | -.250 | .080 | -.008 | .160 |
| AIS_Genchng | 929 | -.426 | .080 | .463 | .160 |
| AIS_EnvIss | 929 | .418 | .080 | .022 | .160 |
| AIS_SocAtt | 929 | .565 | .080 | .289 | .160 |
| EPQ_Idealism | 929 | -1.196 | .080 | 1.162 | .160 |
| EPQ_Relativism | 929 | -.568 | .080 | -.017 | .160 |
| ROS_PersIntExt | 929 | -.751 | .080 | 1.430 | .160 |
| ROS_Soc | 929 | .195 | .080 | -.495 | .160 |
| Valid N (listwise) | 929 |  |  |  |  |

# References

[1] Kim H-Y. Statistical notes for clinical researchers: assessing normal distribution (2) using skewness and kurtosis. *Restor Dent Endod* 2013; 38: 52.
