# Supplementary material for: Do religious beliefs influence concerns for animal welfare? the role of religious orientation and ethical ideologies in attitudes toward animal protection amongst Muslim teachers and school staff in East Java, Indonesia
Source: PLoS One. 2021 Jul 16;16(7):e0254880. doi: 10.1371/journal.pone.0254880 (PMC8284611; doi:10.1371/journal.pone.0254880)
Supplement: S1 File — (DOCX) [file pone.0254880.s004.docx]

Table 1 Main variables and demographical and other determinants to AIS Animal use subscale

| **Model** | **AIS-Animal use issue** | | | | | CI (95%) | | | |  |
| --- | --- | --- | --- | --- | --- | --- | --- | --- | --- | --- |
|  | b | Std. b | | Effect Size | | | Lower | | Upper | |
| **Model 1^A^ - R = 0.11, R^2^ = 0.01, df = 4, 924** |  |  |  |  |  | | |  |  | |
| (Constant) | 2.48 |  | ** |  |  | | | 2.067 | 2.889 | |
| EPQ Ideal | 0.04 | 0.07 | * | 0.00^C^ |  | | | 0.002 | 0.078 | |
| EPQ Relative | 0.01 | 0.02 |  | 0.00^C^ |  | | | -0.022 | 0.041 | |
| ROS Personal | 0.05 | 0.04 |  | 0.00^C^ |  | | | -0.033 | 0.130 | |
| ROS Social | 0.03 | 0.04 |  | 0.00^C^ |  | | | -0.016 | 0.070 | |
|  |  |  |  |  |  | | |  |  | |
| **Model 2^B^ - R = 0.34, R^2^ = 0.11, df = 40, 408** |  |  |  |  |  | | |  |  | |
| (Constant) | 2.27 |  | ** |  |  | | | 1.312 | 3.236 | |
| 1. What is your gender^1^? Female: Yes (1) – No (0) | -0.21 | -0.16 | ** | 0.22^D^ | + | -0.228 | | | -0.060 | |
| 50. How often do you consume meat in a week^2^? Two to three days a week: Yes (1) – No (0) | 0.20 | 0.15 | * | 0.08^D^ |  | -0.160 | | | 0.057 | |
| ROS Personal | 0.16 | 0.12 | * | 0.01^C^ |  | 0.026 | | | 0.301 | |
| 52. How often do you consume meat in a week^2^? I don't consume meat: Yes (1) – No (0) | 0.34 | 0.09 | * | 0.09^D^ |  | -0.258 | | | 0.133 | |
| 23. Where is your current residence place? Urban area: Yes (1) – No (0) | -0.14 | -0.11 |  | - |  | -0.286 | | | 0.001 | |
| 49. How often do you consume meat in a week? Everyday: Yes (1) – No (0) | 0.20 | 0.10 |  | - |  | -0.008 | | | 0.400 | |
| 51. How often do you consume meat in a week? Four to six days a week: Yes (1) – No (0) | 0.20 | 0.10 |  | - |  | -0.009 | | | 0.411 | |
| 22. Do you have children? Yes (1) – No (0) | 0.17 | 0.11 |  | - |  | -0.060 | | | 0.399 | |
| 63. How often do you visit a zoo or aquarium? Once every six month: Yes (1) – No (0) | -0.19 | -0.07 |  | - |  | -0.477 | | | 0.097 | |
| 48. What is your gross household expenses per month? Refuse to answer: Yes (1) – No (0) | -0.10 | -0.06 |  | - |  | -0.266 | | | 0.070 | |
| 41. What is your gross household income per month? About twice the average income in my country: Yes (1) – No (0) | -0.26 | -0.06 |  | - |  | -0.715 | | | 0.194 | |
| 2. What is your age? | 0.00 | 0.07 |  | - |  | -0.004 | | | 0.013 | |
| EPQ Ideal | 0.03 | 0.05 |  | - |  | -0.026 | | | 0.085 | |
| EPQ Relative | -0.02 | -0.05 |  | - |  | -0.072 | | | 0.022 | |
| 19. Do you belong or donate to an organization or charity involved in or concerned with: Improving health or human rights: Yes (1) – No (0) | -0.12 | -0.06 |  | - |  | -0.363 | | | 0.118 | |
| 40. What is your gross household income per month? About the average income in my country: Yes (1) – No (0) | -0.09 | -0.06 |  | - |  | -0.279 | | | 0.095 | |

*p<.05; **p<.01; ^A^regression using enter method in a stepwise manner; ^B^regression using enter method; ^C^effect-size calculation using eta squared (F^2^); ^D^effect-size calculation using Hedge’s g; *+*small effect size F^2^>=0.02 (or in some cases of categorical dummy variable, using Cohen’s D/Hedges’g >= 0.2); **++**medium effect size F^2^>=0.15 (or in some cases of categorical dummy variable, using cohen’s D/Hedges’g >=0.5); ^1^compared to male respondents; ^2^compared to respondents who eat meat once a week

Table 1 Main variables and demographical and other determinants to AIS Animal use subscale (continued)

| **Model** | **AIS-Animal use issue** | | | | | CI (95%) | | | |  |
| --- | --- | --- | --- | --- | --- | --- | --- | --- | --- | --- |
|  | b | Std. b | | Effect Size | | | Lower | | Upper | |
| **Model 2^B^ - R = 0.34, R^2^ = 0.11, df = 40, 408 (continued)** |  |  |  |  |  | | |  |  | |
| 17. Do you belong or donate to an organization or charity involved in or concerned with: Animal sector: Yes (1) – No (0) | 0.21 | 0.05 |  | - |  | -0.223 | | | 0.636 | |
| 27. Do you have your own backyard? Yes (1) – No (0) | 0.06 | 0.05 |  | - |  | -0.071 | | | 0.196 | |
| 42. What is your gross household income per month? More than twice the average income in my country: Yes (1) – No (0) | 0.36 | 0.05 |  | - |  | -0.433 | | | 1.160 | |
| 25. In what sort of house do you live? Own house: Yes (1) – No (0) | -0.07 | -0.05 |  | - |  | -0.219 | | | 0.085 | |
| 44. What is your gross household expenses per month? Five to 10 million: Yes (1) – No (0) | -0.09 | -0.04 |  | - |  | -0.301 | | | 0.129 | |
| 43. What is your gross household income per month? Refuse to answer: Yes (1) – No (0) | 0.07 | 0.04 |  | - |  | -0.113 | | | 0.246 | |
| 21. What is your marriage status? Widow(er): Yes (1) – No (0) | -0.20 | -0.04 |  | - |  | -0.731 | | | 0.338 | |
| 38. Is religion important for you? Yes (1) – No (0) | -0.18 | -0.03 |  | - |  | -0.745 | | | 0.380 | |
| 64. How often do you visit a zoo or aquarium? Once a year: Yes (1) – No (0) | -0.06 | -0.04 |  | - |  | -0.252 | | | 0.131 | |
| 47. What is your gross household expenses per month? Above 25 million: Yes (1) – No (0) | -0.29 | -0.03 |  | - |  | -1.226 | | | 0.640 | |
| ROS Social | 0.02 | 0.03 |  | - |  | -0.045 | | | 0.086 | |
| 39. What is your gross household income per month? About the minimum income in my country: Yes (1) – No (0) | -0.06 | -0.03 |  | - |  | -0.265 | | | 0.143 | |
| 65. How often do you visit a zoo or aquarium? Once every two or more year: Yes (1) – No (0) | 0.05 | 0.04 |  | - |  | -0.123 | | | 0.220 | |
| 20. What is your marriage status? Married: Yes (1) – No (0) | -0.07 | -0.05 |  | - |  | -0.337 | | | 0.188 | |
| 13. What is the highest level of schooling you have completed? Senior high: Yes (1) – No (0) | -0.08 | -0.04 |  | - |  | -0.375 | | | 0.213 | |
| 26. In what sort of house do you live? Room rent: Yes (1) – No (0) | 0.06 | 0.03 |  | - |  | -0.168 | | | 0.285 | |
| 3. Do you have any affiliation to religious organization? Yes (1) – No (0) | -0.03 | -0.02 |  | - |  | -0.190 | | | 0.132 | |
| 24. In what sort of house do you live? Apartment: Yes (1) – No (0) | 0.11 | 0.02 |  | - |  | -0.504 | | | 0.726 | |
| 15. What is the highest level of schooling you have completed? Bachelor: Yes (1) – No (0) | 0.03 | 0.02 |  | - |  | -0.156 | | | 0.221 | |
| 14. What is the highest level of schooling you have completed? Diploma: Yes (1) – No (0) | 0.05 | 0.01 |  | - |  | -0.437 | | | 0.533 | |

Table 1 Main variables and demographical and other determinants to AIS Animal use subscale (continued)

| **Model** | **AIS-Animal use issue** | | | | | CI (95%) | | | |  |
| --- | --- | --- | --- | --- | --- | --- | --- | --- | --- | --- |
|  | b | Std. b | | Effect Size | | | Lower | | Upper | |
| **Model 2^B^ - R = 0.34, R^2^ = 0.11, df = 40, 408 (continued)** |  |  |  |  |  | | |  |  | |
| 45. What is your gross household expenses per month? 10 to 15 million: Yes (1) – No (0) | -0.06 | -0.01 |  | - |  | -0.830 | | | 0.705 | |
| 18. Do you belong or donate to an organization or charity involved in or concerned with: Conservation of the natural environment: Yes (1) – No (0) | -0.02 | -0.01 |  | - |  | -0.299 | | | 0.265 | |
| 53. Do you have pet? Yes (1) – No (0) | -0.01 | 0.00 |  | - |  | -0.139 | | | 0.127 | |
| 62. How often do you visit a zoo or aquarium? Once a month: Yes (1) – No (0) | 0.00 | 0.00 |  | - |  | -0.373 | | | 0.365 | |

Table 2 Main variables and demographical and other determinants to AIS Integrity destruction subscale

| **Model** | **AIS-Integrity destruction issue** | | | | | | CI (95%) | | |  |  |
| --- | --- | --- | --- | --- | --- | --- | --- | --- | --- | --- | --- |
|  | b | Std. b | | | Effect Size | | Lower | | Upper | |  |
| **Model 1 - R = 0.29, R^2^ = 0.08, df = 4, 924** |  |  |  | |  |  |  | |  | |  |
| (Constant) | 2.89 |  | ** | |  |  | 2.413 | | 3.370 | |  |
| EPQ Ideal | 0.01 | 0.01 |  | | 0.00^C^ |  | -0.036 | | 0.052 | |  |
| EPQ Relative | 0.05 | 0.10 | ** | | 0.01^C^ |  | 0.015 | | 0.088 | |  |
| ROS Personal | -0.31 | -0.21 | ** | | 0.04^C^ | + | -0.402 | | -0.212 | |  |
| ROS Social | 0.14 | 0.18 | ** | | 0.03^C^ | + | 0.093 | | 0.193 | |  |
|  |  |  |  | |  |  |  | |  | |  |
| **Model 2^B^ - R = 0.30, R^2^ = 0.09, df = 40, 408** |  |  |  |  | |  | |  |  | | |
| (Constant) | 2.70 |  | ** |  | |  | | 1.652 | 3.747 | | |
| 25. In what sort of house do you live^1^? Own house: Yes (1) – No (0) | 0.22 | 0.16 | ** | | 0.14^D^ |  | -0.002 | | 0.220 | |  |
| 48. What is your gross household expenses per month^2^? Refuse to answer: Yes (1) – No (0) | -0.19 | -0.11 | * | | 0.14^D^ |  | -0.013 | | 0.225 | |  |
| 14. What is the highest level of schooling you have completed? Diploma: Yes (1) – No (0) | 0.52 | 0.10 |  | | - |  | -0.007 | | 1.049 | |  |
| 52. How often do you consume meat in a week? I don't consume meat: Yes (1) – No (0) | 0.30 | 0.10 |  | | - |  | -0.014 | | 0.620 | |  |
| 50. How often do you consume meat in a week? Two to three days a week: Yes (1) – No (0) | 0.16 | 0.11 |  | | - |  | -0.008 | | 0.322 | |  |
| 27. Do you have your own backyard? Yes (1) – No (0) | -0.14 | -0.10 |  | | - |  | -0.282 | | 0.009 | |  |
| 15. What is the highest level of schooling you have completed? Bachelor: Yes (1) – No (0) | 0.18 | 0.11 |  | | - |  | -0.023 | | 0.388 | |  |
| ROS Personal | -0.13 | -0.09 |  | | - |  | -0.279 | | 0.020 | |  |
| 17. Do you belong or donate to an organization or charity involved in or concerned with: Animal sector: Yes (1) – No (0) | -0.39 | -0.09 |  | | - |  | -0.855 | | 0.081 | |  |
| 65. How often do you visit a zoo or aquarium? Once every two or more year: Yes (1) – No (0) | -0.14 | -0.10 |  | | - |  | -0.326 | | 0.048 | |  |
| 18. Do you belong or donate to an organization or charity involved in or concerned with: Conservation of the natural environment: Yes (1) – No (0) | 0.23 | 0.09 |  | | - |  | -0.082 | | 0.533 | |  |
| 63. How often do you visit a zoo or aquarium? Once every six month: Yes (1) – No (0) | -0.21 | -0.08 |  | | - |  | -0.524 | | 0.100 | |  |
| 1. What is your gender? Female: Yes (1) – No (0) | -0.09 | -0.06 |  | | - |  | -0.228 | | 0.048 | |  |
| 47. What is your gross household expenses per month? Above 25 million: Yes (1) – No (0) | -0.59 | -0.06 |  | | - |  | -1.605 | | 0.427 | |  |
| 53. Do you have pet? Yes (1) – No (0) | 0.08 | 0.06 |  | | - |  | -0.061 | | 0.228 | |  |

*p<.05; **p<.01; ^A^regression using enter method in a stepwise manner; ^B^regression using enter method; ^C^effect-size calculation using eta squared (F^2^); ^D^effect-size calculation using Hedge’s g; *+*small effect size F^2^>=0.02 (or in some cases of categorical dummy variable, using Cohen’s D/Hedges’g >= 0.2); **++**medium effect size F^2^>=0.15 (or in some cases of categorical dummy variable, using cohen’s D/Hedges’g >=0.5); ^1^compared to respondents who still live with their parents; ^2^compared to respondents who monthly expenses below IDR 5 million

Table 2 Main variables and demographical and other determinants to AIS Integrity destruction subscale (continued)

| **Model** | **AIS-Integrity destruction issue** | | | | | | CI (95%) | | |  |  |
| --- | --- | --- | --- | --- | --- | --- | --- | --- | --- | --- | --- |
|  | b | Std. b | | | Effect Size | | Lower | | Upper | |  |
| **Model 2^B^ - R = 0.30, R^2^ = 0.09, df = 40, 408 (continued)** |  |  |  |  | |  | |  |  | | |
| 51. How often do you consume meat in a week? Four to six days a week: Yes (1) – No (0) | 0.13 | 0.06 |  | | - |  | -0.102 | | 0.355 | |  |
| 26. In what sort of house do you live? Room rent: Yes (1) – No (0) | 0.10 | 0.05 |  | | - |  | -0.142 | | 0.352 | |  |
| 49. How often do you consume meat in a week? Everyday: Yes (1) – No (0) | 0.09 | 0.04 |  | | - |  | -0.134 | | 0.311 | |  |
| 64. How often do you visit a zoo or aquarium? Once a year: Yes (1) – No (0) | -0.08 | -0.05 |  | | - |  | -0.291 | | 0.127 | |  |
| 24. In what sort of house do you live? Apartment: Yes (1) – No (0) | 0.25 | 0.04 |  | | - |  | -0.416 | | 0.924 | |  |
| 40. What is your gross household income per month? About the average income in my country: Yes (1) – No (0) | -0.07 | -0.04 |  | | - |  | -0.277 | | 0.130 | |  |
| 21. What is your marriage status? Widow(er): Yes (1) – No (0) | -0.18 | -0.04 |  | | - |  | -0.761 | | 0.404 | |  |
| 41. What is your gross household income per month? About twice the average income in my country: Yes (1) – No (0) | -0.13 | -0.03 |  | | - |  | -0.625 | | 0.365 | |  |
| 62. How often do you visit a zoo or aquarium? Once a month: Yes (1) – No (0) | -0.10 | -0.03 |  | | - |  | -0.506 | | 0.298 | |  |
| 23. Where is your current residence place? Urban area: Yes (1) – No (0) | -0.03 | -0.02 |  | | - |  | -0.191 | | 0.122 | |  |
| 3. Do you have any affiliation to religious organization? Yes (1) – No (0) | 0.03 | 0.02 |  | | - |  | -0.146 | | 0.205 | |  |
| 39. What is your gross household income per month? About the minimum income in my country: Yes (1) – No (0) | -0.03 | -0.02 |  | | - |  | -0.254 | | 0.190 | |  |
| 19. Do you belong or donate to an organization or charity involved in or concerned with: Improving health or human rights: Yes (1) – No (0) | 0.03 | 0.01 |  | | - |  | -0.230 | | 0.294 | |  |
| ROS Social | 0.01 | 0.01 |  | | - |  | -0.063 | | 0.080 | |  |
| 2. What is your age? | 0.00 | -0.01 |  | | - |  | -0.010 | | 0.008 | |  |
| 45. What is your gross household expenses per month? 10 to 15 million: Yes (1) – No (0) | -0.09 | -0.01 |  | | - |  | -0.928 | | 0.744 | |  |
| 43. What is your gross household income per month? Refuse to answer: Yes (1) – No (0) | 0.02 | 0.01 |  | | - |  | -0.178 | | 0.214 | |  |
| 42. What is your gross household income per month? More than twice the average income in my country: Yes (1) – No (0) | 0.08 | 0.01 |  | | - |  | -0.789 | | 0.946 | |  |
| 22. Do you have children? Yes (1) – No (0) | -0.02 | -0.01 |  | | - |  | -0.270 | | 0.231 | |  |
| 44. What is your gross household expenses per month? Five to 10 million: Yes (1) – No (0) | -0.02 | -0.01 |  | | - |  | -0.249 | | 0.219 | |  |

Table 2 Main variables and demographical and other determinants to AIS Integrity destruction subscale (continued)

| **Model** | **AIS-Integrity destruction issue** | | | | | | CI (95%) | | |  |  |
| --- | --- | --- | --- | --- | --- | --- | --- | --- | --- | --- | --- |
|  | b | Std. b | | | Effect Size | | Lower | | Upper | |  |
| **Model 2^B^ - R = 0.30, R^2^ = 0.09, df = 40, 408 (continued)** |  |  |  |  | |  | |  |  | | |
| EPQ Relative | 0.00 | 0.01 |  | | - |  | -0.049 | | 0.054 | |  |
| 13. What is the highest level of schooling you have completed? Senior high: Yes (1) – No (0) | 0.01 | 0.01 |  | | - |  | -0.306 | | 0.334 | |  |
| 38. Is religion important for you? Yes (1) – No (0) | 0.03 | 0.00 |  | | - |  | -0.586 | | 0.639 | |  |
| 20. What is your marriage status? Married: Yes (1) – No (0) | -0.01 | 0.00 |  | | - |  | -0.291 | | 0.280 | |  |
| EPQ Ideal | 0.00 | 0.00 |  | | - |  | -0.060 | | 0.062 | |  |

Table 3 Main variables and demographical and other determinants to AIS animal killing and welfare deprivation subscale

| **Model** | **AIS-Animal killing and welfare deprivation issue** | | | | | CI (95%) | | |
| --- | --- | --- | --- | --- | --- | --- | --- | --- |
|  | b | Std. b | | Effect Size | | Lower | | Upper |
| **Model 1 - R = 0.41, R^2^= 0.17, df = 4, 924** |  |  |  |  |  |  | |  |
| (Constant) | 3.40 |  | ** |  |  | 2.961 | | 3.842 |
| EPQ Ideal | -0.08 | -0.13 | ** | 0.01^C^ |  | -0.122 | | -0.041 |
| EPQ Relative | 0.08 | 0.15 | ** | 0.02^C^ | + | 0.047 | | 0.114 |
| ROS Personal | -0.40 | -0.28 | ** | 0.08^C^ | + | -0.485 | | -0.311 |
| ROS Social | 0.16 | 0.21 | ** | 0.05^C^ | + | 0.117 | | 0.209 |
|  |  |  |  |  |  |  | |  |
| **Model 2^B^ - R = 0.47, R^2^ = 0.22, df = 40, 408** |  |  |  |  |  | |  |  |
| (Constant) | 3.18 |  | ** |  |  | | 2.241 | 4.114 |
| ROS Personal | -0.38 | -0.27 | ** | 0.06^C^ | + | -0.513 | | -0.245 |
| 52. How often do you consume meat in a week^1^? I don't consume meat: Yes (1) – No (0) | 0.39 | 0.13 | ** | 0.04^D^ |  | -0.268 | | 0.197 |
| ROS Social | 0.08 | 0.12 | * | 0.01^C^ |  | 0.018 | | 0.146 |
| 1. What is your gender^2^? Female: Yes (1) – No (0) | -0.15 | -0.11 | * | 0.19^D^ |  | -0.241 | | -0.045 |
| EPQ Ideal | -0.06 | -0.11 | * | 0.01^C^ |  | -0.117 | | -0.009 |
| 14. What is the highest level of schooling you have completed^3^? Diploma: Yes (1) – No (0) | 0.54 | 0.11 | * | 0.73^D^ | ++ | 0.256 | | 0.892 |
| EPQ Relative | 0.05 | 0.11 | * | 0.01^C^ |  | 0.005 | | 0.097 |
| 25. In what sort of house do you live^4^? Own house: Yes (1) – No (0) | 0.15 | 0.11 |  | - |  | -0.001 | | 0.295 |
| 53. Do you have pet? Yes (1) – No (0) | -0.11 | -0.08 |  | - |  | -0.235 | | 0.024 |
| 48. What is your gross household expenses per month? Refuse to answer: Yes (1) – No (0) | -0.13 | -0.08 |  | - |  | -0.291 | | 0.036 |
| 44. What is your gross household expenses per month? Five to 10 million: Yes (1) – No (0) | -0.14 | -0.07 |  | - |  | -0.354 | | 0.064 |
| 38. Is religion important for you? Yes (1) – No (0) | 0.37 | 0.06 |  | - |  | -0.174 | | 0.922 |
| 20. What is your marriage status? Married: Yes (1) – No (0) | -0.17 | -0.10 |  | - |  | -0.427 | | 0.084 |
| 45. What is your gross household expenses per month? 10 to 15 million: Yes (1) – No (0) | -0.48 | -0.06 |  | - |  | -1.231 | | 0.263 |
| 13. What is the highest level of schooling you have completed? Senior high: Yes (1) – No (0) | -0.18 | -0.08 |  | - |  | -0.467 | | 0.105 |
| 19. Do you belong or donate to an organization or charity involved in or concerned with: Improving health or human rights: Yes (1) – No (0) | 0.14 | 0.07 |  | - |  | -0.090 | | 0.378 |
| 51. How often do you consume meat in a week? Four to six days a week: Yes (1) – No (0) | -0.12 | -0.06 |  | - |  | -0.326 | | 0.083 |

*p<.05; **p<.01; ^A^regression using enter method in a stepwise manner; ^B^regression using enter method; ^C^effect-size calculation using eta squared (F^2^); ^D^effect-size calculation using Hedge’s g; *+*small effect size F^2^>=0.02 (or in some cases of categorical dummy variable, using Cohen’s D/Hedges’g >= 0.2); **++**medium effect size F^2^>=0.15 (or in some cases of categorical dummy variable, using cohen’s D/Hedges’g >=0.5); ^1^compared to respondents who eat meat once a week; ^2^compared to male respondents; ^3^compared to those respondent with Master/PhD degree; ^4^compared to those who live with their parents

Table 3 Main variables and demographical and other determinants to AIS animal killing and welfare deprivation subscale (continued)

| **Model** | **AIS-Animal killing and welfare deprivation issue** | | | | | CI (95%) | | |
| --- | --- | --- | --- | --- | --- | --- | --- | --- |
|  | b | Std. b | | Effect Size | | Lower | | Upper |
| **Model 2^B^ - R = 0.47, R^2^ = 0.22, df = 40, 408 (continued)** |  |  |  |  |  | |  |  |
| 26. In what sort of house do you live? Room rent: Yes (1) – No (0) | 0.12 | 0.06 |  | - |  | -0.104 | | 0.337 |
| 65. How often do you visit a zoo or aquarium? Once every two or more year: Yes (1) – No (0) | -0.08 | -0.06 |  | - |  | -0.251 | | 0.083 |
| 15. What is the highest level of schooling you have completed? Bachelor: Yes (1) – No (0) | 0.09 | 0.06 |  | - |  | -0.092 | | 0.275 |
| 63. How often do you visit a zoo or aquarium? Once every six month: Yes (1) – No (0) | -0.13 | -0.05 |  | - |  | -0.413 | | 0.145 |
| 41. What is your gross household income per month? About twice the average income in my country: Yes (1) – No (0) | 0.21 | 0.04 |  | - |  | -0.237 | | 0.648 |
| 43. What is your gross household income per month? Refuse to answer: Yes (1) – No (0) | 0.08 | 0.05 |  | - |  | -0.095 | | 0.256 |
| 47. What is your gross household expenses per month? Above 25 million: Yes (1) – No (0) | -0.40 | -0.04 |  | - |  | -1.313 | | 0.505 |
| 22. Do you have children? Yes (1) – No (0) | 0.10 | 0.06 |  | - |  | -0.127 | | 0.320 |
| 3. Do you have any affiliation to religious organization? Yes (1) – No (0) | 0.05 | 0.04 |  | - |  | -0.103 | | 0.210 |
| 18. Do you belong or donate to an organization or charity involved in or concerned with: Conservation of the natural environment: Yes (1) – No (0) | 0.09 | 0.04 |  | - |  | -0.185 | | 0.365 |
| 23. Where is your current residence place? Urban area: Yes (1) – No (0) | -0.04 | -0.03 |  | - |  | -0.182 | | 0.097 |
| 50. How often do you consume meat in a week? Two to three days a week: Yes (1) – No (0) | -0.04 | -0.03 |  | - |  | -0.189 | | 0.106 |
| 62. How often do you visit a zoo or aquarium? Once a month: Yes (1) – No (0) | -0.09 | -0.02 |  | - |  | -0.451 | | 0.268 |
| 64. How often do you visit a zoo or aquarium? Once a year: Yes (1) – No (0) | 0.04 | 0.02 |  | - |  | -0.148 | | 0.225 |
| 17. Do you belong or donate to an organization or charity involved in or concerned with: Animal sector: Yes (1) – No (0) | 0.08 | 0.02 |  | - |  | -0.341 | | 0.496 |
| 2. What is your age? | 0.00 | 0.02 |  | - |  | -0.007 | | 0.010 |
| 39. What is your gross household income per month? About the minimum income in my country: Yes (1) – No (0) | 0.03 | 0.01 |  | - |  | -0.169 | | 0.227 |
| 42. What is your gross household income per month? More than twice the average income in my country: Yes (1) – No (0) | 0.10 | 0.01 |  | - |  | -0.673 | | 0.878 |
| 21. What is your marriage status? Widow(er): Yes (1) – No (0) | -0.06 | -0.01 |  | - |  | -0.579 | | 0.462 |

Table 3 Main variables and demographical and other determinants to AIS animal killing and welfare deprivation subscale (continued)

| **Model** | **AIS-Animal killing and welfare deprivation issue** | | | | | CI (95%) | | |
| --- | --- | --- | --- | --- | --- | --- | --- | --- |
|  | b | Std. b | | Effect Size | | Lower | | Upper |
| **Model 2^B^ - R = 0.47, R^2^ = 0.22, df = 40, 408 (continued)** |  |  |  |  |  | |  |  |
| 27. Do you have your own backyard? Yes (1) – No (0) | -0.01 | -0.01 |  | - |  | -0.144 | | 0.115 |
| 40. What is your gross household income per month? About the average income in my country: Yes (1) – No (0) | 0.02 | 0.01 |  | - |  | -0.164 | | 0.201 |
| 24. In what sort of house do you live? Apartment: Yes (1) – No (0) | 0.05 | 0.01 |  | - |  | -0.553 | | 0.645 |
| 49. How often do you consume meat in a week? Everyday: Yes (1) – No (0) | 0.01 | 0.00 |  | - |  | -0.191 | | 0.206 |

Table 4 Main variables and demographical and other determinants to AIS animal experimentation subscale

| **Model** | **AIS-Animal experimentation issue** | | | | | | | | | | CI (95%) | | | | | | |  |
| --- | --- | --- | --- | --- | --- | --- | --- | --- | --- | --- | --- | --- | --- | --- | --- | --- | --- | --- |
|  | b | Std. b | | | Effect Size | | | | | | | Lower | | | Upper |  |  |  |
| **Model 1 - R = 0.14, R^2^= 0.02, df = 4, 924** |  |  |  | |  | |  | | |  | | | | |  | | | |
| (Constant) | 2.14 |  | ** | |  | |  | | | 1.622 | | | | | 2.667 | | | |
| EPQ Ideal | 0.07 | 0.11 | ** | | 0.01^C^ | |  | | | 0.025 | | | | | 0.121 | | | |
| EPQ Relative | 0.00 | 0.00 |  | | 0.00^C^ | |  | | | -0.042 | | | | | 0.038 | | | |
| ROS Personal | 0.04 | 0.02 |  | | 0.00^C^ | |  | | | -0.068 | | | | | 0.139 | | | |
| ROS Social | 0.07 | 0.08 | * | | 0.01^C^ | |  | | | 0.016 | | | | | 0.125 | | | |
|  |  |  |  | |  | |  | | |  | | | | |  | | | |
| **Model 2^B^ - R = 0.35, R^2^ = 0.12, df = 40, 408** |  |  |  |  | |  | | |  | | | |  | | |  |  |  |
| (Constant) | 1.52 |  | * |  | |  | | | 0.340 | | | | 2.701 | | |  |  |  |
| 49. How often do you consume meat in a week^1^? Everyday: Yes (1) – No (0) | 0.30 | 0.13 | * | | 0.20 ^D^ | | + | 0.053 | | | | | | 0.554 | | |  |  |
| 52. How often do you consume meat in a week^1^? I don't consume meat: Yes (1) – No (0) | 0.43 | 0.12 | * | | 0.20 ^D^ | |  | -0.409 | | | | | | 0.071 | | |  |  |
| 2. What is your age? | 0.01 | 0.15 | * | | 0.01^C^ | |  | 0.002 | | | | | | 0.023 | | |  |  |
| ROS Social | 0.09 | 0.11 | * | | 0.01^C^ | |  | 0.007 | | | | | | 0.168 | | |  |  |
| 14. What is the highest level of schooling you have completed^2^? Diploma: Yes (1) – No (0) | 0.62 | 0.11 | * | | 0.44^D^ | | + | 0.024 | | | | | | 0.704 | | |  |  |
| 1. What is your gender? Female: Yes (1) – No (0) | -0.14 | -0.09 |  | | - | |  | -0.298 | | | | | | 0.013 | | |  |  |
| EPQ Ideal | 0.06 | 0.09 |  | | - | |  | -0.007 | | | | | | 0.130 | | |  |  |
| 39. What is your gross household income per month? About the minimum income in my country: Yes (1) – No (0) | 0.19 | 0.08 |  | | - | |  | -0.063 | | | | | | 0.437 | | |  |  |
| 44. What is your gross household expenses per month? Five to 10 million: Yes (1) – No (0) | -0.20 | -0.08 |  | | - | |  | -0.460 | | | | | | 0.067 | | |  |  |
| 50. How often do you consume meat in a week? Two to three days a week: Yes (1) – No (0) | 0.14 | 0.08 |  | | - | |  | -0.048 | | | | | | 0.324 | | |  |  |
| EPQ Relative | -0.04 | -0.08 |  | | - | |  | -0.100 | | | | | | 0.015 | | |  |  |
| 64. How often do you visit a zoo or aquarium? Once a year: Yes (1) – No (0) | 0.14 | 0.08 |  | | - | |  | -0.092 | | | | | | 0.379 | | |  |  |
| 47. What is your gross household expenses per month? Above 25 million: Yes (1) – No (0) | -0.68 | -0.06 |  | | - | |  | -1.828 | | | | | | 0.461 | | |  |  |
| 23. Where is your current residence place? Urban area: Yes (1) – No (0) | -0.10 | -0.06 |  | | - | |  | -0.278 | | | | | | 0.074 | | |  |  |
| 40. What is your gross household income per month? About the average income in my country: Yes (1) – No (0) | 0.13 | 0.07 |  | | - | |  | -0.100 | | | | | | 0.359 | | |  |  |
| 20. What is your marriage status? Married: Yes (1) – No (0) | -0.18 | -0.09 |  | | - | |  | -0.499 | | | | | | 0.144 | | |  |  |

*p<.05; **p<.01; ^A^regression using enter method in a stepwise manner; ^B^regression using enter method; ^C^effect-size calculation using eta squared (F^2^); ^D^effect-size calculation using Hedge’s g; *+*small effect size F^2^>=0.02 (or in some cases of categorical dummy variable, using Cohen’s D/Hedges’g >= 0.2); **++**medium effect size F^2^>=0.15 (or in some cases of categorical dummy variable, using cohen’s D/Hedges’g >=0.5); ^1^compared to respondents who eat meat once a week; ^2^compared to those respondent with Master/PhD degree

Table 4 Main variables and demographical and other determinants to AIS animal experimentation subscale (continued)

| **Model** | **AIS-Animal experimentation issue** | | | | | | | | | CI (95%) | | | | | | |
| --- | --- | --- | --- | --- | --- | --- | --- | --- | --- | --- | --- | --- | --- | --- | --- | --- |
|  | b | Std. b | | | Effect Size | | | | | | Lower | | | Upper |  |  |
| **Model 2^B^ - R = 0.35, R^2^ = 0.12, df = 40, 408 (continued)** |  |  |  |  | |  | | |  | | |  | | |  |  |
| 24. In what sort of house do you live? Apartment: Yes (1) – No (0) | -0.40 | -0.05 |  | | - | |  | -1.159 | | | | | 0.350 | | |  |
| 22. Do you have children? Yes (1) – No (0) | 0.14 | 0.08 |  | | - | |  | -0.139 | | | | | 0.424 | | |  |
| ROS Personal | 0.08 | 0.05 |  | | - | |  | -0.084 | | | | | 0.253 | | |  |
| 27. Do you have your own backyard? Yes (1) – No (0) | -0.07 | -0.05 |  | | - | |  | -0.237 | | | | | 0.090 | | |  |
| 53. Do you have pet? Yes (1) – No (0) | -0.07 | -0.04 |  | | - | |  | -0.235 | | | | | 0.091 | | |  |
| 42. What is your gross household income per month? More than twice the average income in my country: Yes (1) – No (0) | 0.41 | 0.04 |  | | - | |  | -0.562 | | | | | 1.392 | | |  |
| 21. What is your marriage status? Widow(er): Yes (1) – No (0) | -0.27 | -0.05 |  | | - | |  | -0.929 | | | | | 0.382 | | |  |
| 51. How often do you consume meat in a week? Four to six days a week: Yes (1) – No (0) | 0.11 | 0.04 |  | | - | |  | -0.151 | | | | | 0.364 | | |  |
| 65. How often do you visit a zoo or aquarium? Once every two or more year: Yes (1) – No (0) | 0.08 | 0.05 |  | | - | |  | -0.131 | | | | | 0.289 | | |  |
| 15. What is the highest level of schooling you have completed? Bachelor: Yes (1) – No (0) | 0.07 | 0.04 |  | | - | |  | -0.160 | | | | | 0.302 | | |  |
| 62. How often do you visit a zoo or aquarium? Once a month: Yes (1) – No (0) | 0.13 | 0.03 |  | | - | |  | -0.320 | | | | | 0.586 | | |  |
| 25. In what sort of house do you live? Own house: Yes (1) – No (0) | 0.04 | 0.03 |  | | - | |  | -0.146 | | | | | 0.227 | | |  |
| 38. Is religion important for you? Yes (1) – No (0) | 0.14 | 0.02 |  | | - | |  | -0.553 | | | | | 0.827 | | |  |
| 63. How often do you visit a zoo or aquarium? Once every six month: Yes (1) – No (0) | -0.07 | -0.02 |  | | - | |  | -0.422 | | | | | 0.282 | | |  |
| 48. What is your gross household expenses per month? Refuse to answer: Yes (1) – No (0) | 0.03 | 0.02 |  | | - | |  | -0.171 | | | | | 0.241 | | |  |
| 19. Do you belong or donate to an organization or charity involved in or concerned with: Improving health or human rights: Yes (1) – No (0) | 0.04 | 0.02 |  | | - | |  | -0.252 | | | | | 0.339 | | |  |
| 43. What is your gross household income per month? Refuse to answer: Yes (1) – No (0) | 0.03 | 0.02 |  | | - | |  | -0.191 | | | | | 0.250 | | |  |
| 45. What is your gross household expenses per month? 10 to 15 million: Yes (1) – No (0) | 0.13 | 0.01 |  | | - | |  | -0.815 | | | | | 1.068 | | |  |
| 18. Do you belong or donate to an organization or charity involved in or concerned with: Conservation of the natural environment: Yes (1) – No (0) | 0.04 | 0.02 |  | | - | |  | -0.303 | | | | | 0.390 | | |  |

Table 4 Main variables and demographical and other determinants to AIS animal experimentation subscale (continued)

| **Model** | **AIS-Animal experimentation issue** | | | | | | | | | CI (95%) | | | | | | |
| --- | --- | --- | --- | --- | --- | --- | --- | --- | --- | --- | --- | --- | --- | --- | --- | --- |
|  | b | Std. b | | | Effect Size | | | | | | Lower | | | Upper |  |  |
| **Model 2^B^ - R = 0.35, R^2^ = 0.12, df = 40, 408 (continued)** |  |  |  |  | |  | | |  | | |  | | |  |  |
| 41. What is your gross household income per month? About twice the average income in my country: Yes (1) – No (0) | -0.07 | -0.01 |  | | - | |  | -0.624 | | | | | 0.491 | | |  |
| 13. What is the highest level of schooling you have completed? Senior high: Yes (1) – No (0) | -0.02 | -0.01 |  | | - | |  | -0.385 | | | | | 0.336 | | |  |
| 26. In what sort of house do you live? Room rent: Yes (1) – No (0) | 0.01 | 0.01 |  | | - | |  | -0.264 | | | | | 0.292 | | |  |
| 3. Do you have any affiliation to religious organization? Yes (1) – No (0) | -0.01 | -0.01 |  | | - | |  | -0.207 | | | | | 0.189 | | |  |
| 17. Do you belong or donate to an organization or charity involved in or concerned with: Animal sector: Yes (1) – No (0) | -0.01 | 0.00 |  | | - | |  | -0.538 | | | | | 0.516 | | |  |

Table 5 Main variables and demographical and other determinants to AIS animal genetic change subscale

| **Model** | **AIS-Animal genetic change issue** | | | | | | | | | CI (95%) | | | | | |
| --- | --- | --- | --- | --- | --- | --- | --- | --- | --- | --- | --- | --- | --- | --- | --- |
|  | b | Std. b | | | Effect Size | | | | | Lower | | | Upper | | |
| **Model 1 - R = 0.26, R^2^= 0.07, df = 4, 924** |  |  |  | |  | |  | | |  | | |  | | |
| (Constant) | 1.41 |  | ** | |  | |  | | | 0.956 | | | 1.866 | | |
| EPQ Ideal | 0.09 | 0.14 | ** | | 0.02^C^ | |  | | | 0.046 | | | 0.129 | | |
| EPQ Relative | 0.02 | 0.04 |  | | 0.00^C^ | |  | | | -0.016 | | | 0.054 | | |
| ROS Personal | 0.24 | 0.17 | ** | | 0.03^C^ | | + | | | 0.152 | | | 0.332 | | |
| ROS Social | 0.04 | 0.06 |  | | 0.00^C^ | |  | | | -0.004 | | | 0.091 | | |
|  |  |  |  | |  | |  | | |  | | |  | | |
| **Model 2^B^ - R = 0.40, R^2^ = 0.16, df = 40, 408** |  |  |  |  | |  | | |  | |  | | |  |  |
| (Constant) | 0.69 |  |  |  | |  | | | -0.347 | | 1.722 | | |  |  |
| ROS Personal | 0.30 | 0.20 | ** | | 0.03^C^ | | + | 0.148 | | | | 0.443 | | |  |
| 52. How often do you consume meat in a week^1^? I don't consume meat: Yes (1) – No (0) | 0.44 | 0.14 | ** | | 0.22^D^ | | + | -.0382 | | | | 0.046 | | |  |
| EPQ Ideal | 0.08 | 0.14 | ** | | 0.01^C^ | |  | 0.021 | | | | 0.141 | | |  |
| 23. Where is your current residence place^2^? Urban area: Yes (1) – No (0) | -0.16 | -0.11 | * | | 0.05^D^ | |  | -0.136 | | | | 0.062 | | |  |
| 22. Do you have children? Yes (1) – No (0) | 0.25 | 0.15 |  | | - | |  | -0.002 | | | | 0.492 | | |  |
| 24. In what sort of house do you live? Apartment: Yes (1) – No (0) | 0.59 | 0.09 |  | | - | |  | -0.073 | | | | 1.250 | | |  |
| 50. How often do you consume meat in a week? Two to three days a week: Yes (1) – No (0) | 0.14 | 0.10 |  | | - | |  | -0.023 | | | | 0.303 | | |  |
| 20. What is your marriage status? Married: Yes (1) – No (0) | -0.23 | -0.13 |  | | - | |  | -0.513 | | | | 0.052 | | |  |
| 25. In what sort of house do you live? Own house: Yes (1) – No (0) | 0.13 | 0.09 |  | | - | |  | -0.030 | | | | 0.296 | | |  |
| 49. How often do you consume meat in a week? Everyday: Yes (1) – No (0) | 0.16 | 0.08 |  | | - | |  | -0.063 | | | | 0.376 | | |  |
| 38. Is religion important for you? Yes (1) – No (0) | 0.39 | 0.06 |  | | - | |  | -0.215 | | | | 0.995 | | |  |
| 51. How often do you consume meat in a week? Four to six days a week: Yes (1) – No (0) | 0.14 | 0.07 |  | | - | |  | -0.084 | | | | 0.367 | | |  |
| 47. What is your gross household expenses per month? Above 25 million: Yes (1) – No (0) | -0.53 | -0.05 |  | | - | |  | -1.534 | | | | 0.473 | | |  |
| ROS Social | 0.04 | 0.05 |  | | - | |  | -0.034 | | | | 0.107 | | |  |
| 14. What is the highest level of schooling you have completed? Diploma: Yes (1) – No (0) | 0.26 | 0.05 |  | | - | |  | -0.260 | | | | 0.782 | | |  |
| 2. What is your age? | 0.00 | 0.06 |  | | - | |  | -0.005 | | | | 0.014 | | |  |
| 62. How often do you visit a zoo or aquarium? Once a month: Yes (1) – No (0) | -0.18 | -0.04 |  | | - | |  | -0.574 | | | | 0.220 | | |  |
| 21. What is your marriage status? Widow(er): Yes (1) – No (0) | -0.26 | -0.05 |  | | - | |  | -0.831 | | | | 0.319 | | |  |

*p<.05; **p<.01; ^A^regression using enter method in a stepwise manner; ^B^regression using enter method; ^C^effect-size calculation using eta squared (F^2^); ^D^effect-size calculation using Hedge’s g; *+*small effect size F^2^>=0.02 (or in some cases of categorical dummy variable, using Cohen’s D/Hedges’g >= 0.2); **++**medium effect size F^2^>=0.15 (or in some cases of categorical dummy variable, using cohen’s D/Hedges’g >=0.5); ^1^compared to respondents who eat meat once a week; ^2^compared respondent who live in rural.

Table 5 Main variables and demographical and other determinants to AIS animal genetic change subscale (continued)

| **Model** | **AIS-Animal genetic change issue** | | | | | | | | | CI (95%) | | | | | |
| --- | --- | --- | --- | --- | --- | --- | --- | --- | --- | --- | --- | --- | --- | --- | --- |
|  | b | Std. b | | | Effect Size | | | | | Lower | | | Upper | | |
| **Model 2^B^ - R = 0.40, R^2^ = 0.16, df = 40, 408 (continued)** |  |  |  |  | |  | | |  | |  | | |  |  |
| 53. Do you have pet? Yes (1) – No (0) | -0.06 | -0.04 |  | | - | |  | -0.205 | | | | 0.081 | | |  |
| 43. What is your gross household income per month? Refuse to answer: Yes (1) – No (0) | -0.08 | -0.05 |  | | - | |  | -0.275 | | | | 0.112 | | |  |
| 1. What is your gender? Female: Yes (1) – No (0) | -0.05 | -0.04 |  | | - | |  | -0.189 | | | | 0.084 | | |  |
| 65. How often do you visit a zoo or aquarium? Once every two or more year: Yes (1) – No (0) | 0.07 | 0.05 |  | | - | |  | -0.115 | | | | 0.254 | | |  |
| 45. What is your gross household expenses per month? 10 to 15 million: Yes (1) – No (0) | -0.30 | -0.03 |  | | - | |  | -1.124 | | | | 0.526 | | |  |
| 19. Do you belong or donate to an organization or charity involved in or concerned with: Improving health or human rights: Yes (1) – No (0) | 0.08 | 0.04 |  | | - | |  | -0.176 | | | | 0.341 | | |  |
| 44. What is your gross household expenses per month? Five to 10 million: Yes (1) – No (0) | -0.07 | -0.03 |  | | - | |  | -0.303 | | | | 0.159 | | |  |
| 42. What is your gross household income per month? More than twice the average income in my country: Yes (1) – No (0) | -0.25 | -0.03 |  | | - | |  | -1.107 | | | | 0.605 | | |  |
| 48. What is your gross household expenses per month? Refuse to answer: Yes (1) – No (0) | 0.05 | 0.03 |  | | - | |  | -0.132 | | | | 0.230 | | |  |
| 17. Do you belong or donate to an organization or charity involved in or concerned with: Animal sector: Yes (1) – No (0) | 0.12 | 0.03 |  | | - | |  | -0.339 | | | | 0.585 | | |  |
| 26. In what sort of house do you live? Room rent: Yes (1) – No (0) | 0.06 | 0.03 |  | | - | |  | -0.183 | | | | 0.304 | | |  |
| 3. Do you have any affiliation to religious organization? Yes (1) – No (0) | -0.04 | -0.03 |  | | - | |  | -0.215 | | | | 0.132 | | |  |
| 15. What is the highest level of schooling you have completed? Bachelor: Yes (1) – No (0) | 0.04 | 0.03 |  | | - | |  | -0.159 | | | | 0.247 | | |  |
| 27. Do you have your own backyard? Yes (1) – No (0) | 0.03 | 0.02 |  | | - | |  | -0.113 | | | | 0.174 | | |  |
| 40. What is your gross household income per month? About the average income in my country: Yes (1) – No (0) | 0.04 | 0.02 |  | | - | |  | -0.164 | | | | 0.238 | | |  |
| 63. How often do you visit a zoo or aquarium? Once every six month: Yes (1) – No (0) | -0.05 | -0.02 |  | | - | |  | -0.361 | | | | 0.256 | | |  |
| 64. How often do you visit a zoo or aquarium? Once a year: Yes (1) – No (0) | -0.03 | -0.02 |  | | - | |  | -0.241 | | | | 0.171 | | |  |
| 39. What is your gross household income per month? About the minimum income in my country: Yes (1) – No (0) | -0.03 | -0.01 |  | | - | |  | -0.247 | | | | 0.191 | | |  |
| EPQ Relative | 0.00 | 0.01 |  | | - | |  | -0.046 | | | | 0.056 | | |  |

Table 5 Main variables and demographical and other determinants to AIS animal genetic change subscale (continued)

| **Model** | **AIS-Animal genetic change issue** | | | | | | | | | CI (95%) | | | | | |
| --- | --- | --- | --- | --- | --- | --- | --- | --- | --- | --- | --- | --- | --- | --- | --- |
|  | b | Std. b | | | Effect Size | | | | | Lower | | | Upper | | |
| **Model 2^B^ - R = 0.40, R^2^ = 0.16, df = 40, 408 (continued)** |  |  |  |  | |  | | |  | |  | | |  |  |
| 18. Do you belong or donate to an organization or charity involved in or concerned with: Conservation of the natural environment: Yes (1) – No (0) | -0.03 | -0.01 |  | | - | |  | -0.329 | | | | 0.278 | | |  |
| 41. What is your gross household income per month? About twice the average income in my country: Yes (1) – No (0) | 0.01 | 0.00 |  | | - | |  | -0.475 | | | | 0.503 | | |  |
| 13. What is the highest level of schooling you have completed? Senior high: Yes (1) – No (0) | 0.00 | 0.00 |  | | - | |  | -0.314 | | | | 0.318 | | |  |

Table 6 Main variables and demographical and other determinants to AIS harm animal for environmental issue subscale

| **Model** | **AIS-Harm animal for environmental issue** | | | | | | | | | CI (95%) | | | | | |
| --- | --- | --- | --- | --- | --- | --- | --- | --- | --- | --- | --- | --- | --- | --- | --- |
|  | b | Std. b | | | Effect Size | | | | | Lower | | | Upper | | |
| **Model 1 - R = 0.30, R^2^= 0.09, df = 4, 924** |  |  |  | |  | |  | | |  | | |  | | |
| (Constant) | 3.22 |  | ** | |  | |  | | | 2.739 | | | 3.691 | | |
| EPQ Ideal | -0.08 | -0.12 | ** | | 0.01^C^ | |  | | | -0.121 | | | -0.033 | | |
| EPQ Relative | 0.06 | 0.11 | ** | | 0.01^C^ | |  | | | 0.022 | | | 0.095 | | |
| ROS Personal | -0.25 | -0.17 | ** | | 0.03^C^ | | + | | | -0.345 | | | -0.157 | | |
| ROS Social | 0.14 | 0.18 | ** | | 0.03^C^ | | + | | | 0.093 | | | 0.193 | | |
|  |  |  |  | |  | |  | | |  | | |  | | |
| **Model 2^B^ - R = 0.37, R^2^ = 0.13, df = 40, 408** |  |  |  |  | |  | | |  | |  | | |  |  |
| (Constant) | 3.07 |  | ** |  | |  | | | 2.014 | | 4.129 | | |  |  |
| EPQ Ideal | -0.11 | -0.18 | ** | | 0.03^C^ | | + | -0.170 | | | | -0.047 | | |  |
| 52. How often do you consume meat in a week^1^? I don't consume meat: Yes (1) – No (0) | 0.40 | 0.13 | * | | 0.06^D^ | |  | -0.277 | | | | 0.184 | | |  |
| 1. What is your gender^2^? Female: Yes (1) – No (0) | -0.17 | -0.11 | * | | 0.15^D^ | |  | -0.219 | | | | -0.016 | | |  |
| ROS Personal | -0.14 | -0.09 |  | | - | |  | -0.289 | | | | 0.013 | | |  |
| ROS Social | 0.06 | 0.08 |  | | - | |  | -0.014 | | | | 0.130 | | |  |
| 14. What is the highest level of schooling you have completed? Diploma: Yes (1) – No (0) | 0.39 | 0.08 |  | | - | |  | -0.141 | | | | 0.924 | | |  |
| 2. What is your age? | 0.01 | 0.10 |  | | - | |  | -0.003 | | | | 0.016 | | |  |
| 25. In what sort of house do you live? Own house: Yes (1) – No (0) | 0.12 | 0.08 |  | | - | |  | -0.046 | | | | 0.287 | | |  |
| 42. What is your gross household income per month? More than twice the average income in my country: Yes (1) – No (0) | 0.61 | 0.07 |  | | - | |  | -0.265 | | | | 1.485 | | |  |
| 63. How often do you visit a zoo or aquarium? Once every six month: Yes (1) – No (0) | -0.22 | -0.08 |  | | - | |  | -0.533 | | | | 0.098 | | |  |
| EPQ Relative | 0.04 | 0.07 |  | | - | |  | -0.016 | | | | 0.088 | | |  |
| 24. In what sort of house do you live? Apartment: Yes (1) – No (0) | 0.46 | 0.07 |  | | - | |  | -0.216 | | | | 1.136 | | |  |
| 17. Do you belong or donate to an organization or charity involved in or concerned with: Animal sector: Yes (1) – No (0) | -0.31 | -0.07 |  | | - | |  | -0.782 | | | | 0.162 | | |  |
| 41. What is your gross household income per month? About twice the average income in my country: Yes (1) – No (0) | 0.31 | 0.06 |  | | - | |  | -0.192 | | | | 0.807 | | |  |
| 15. What is the highest level of schooling you have completed? Bachelor: Yes (1) – No (0) | 0.11 | 0.06 |  | | - | |  | -0.102 | | | | 0.312 | | |  |
| 27. Do you have your own backyard? Yes (1) – No (0) | -0.07 | -0.05 |  | | - | |  | -0.220 | | | | 0.073 | | |  |
| 49. How often do you consume meat in a week? Everyday: Yes (1) – No (0) | -0.10 | -0.05 |  | | - | |  | -0.320 | | | | 0.129 | | |  |

*p<.05; **p<.01; ^A^regression using enter method in a stepwise manner; ^B^regression using enter method; ^C^effect-size calculation using eta squared (F^2^); ^D^effect-size calculation using Hedge’s g; *+*small effect size F^2^>=0.02 (or in some cases of categorical dummy variable, using Cohen’s D/Hedges’g >= 0.2); **++**medium effect size F^2^>=0.15 (or in some cases of categorical dummy variable, using cohen’s D/Hedges’g >=0.5); ^1^compared to respondents who eat meat once a week; ^2^compared to male respondent.

Table 6 Main variables and demographical and other determinants to AIS harm animal for environmental issue subscale (continued)

| **Model** | **AIS-Harm animal for environmental issue** | | | | | | | | | CI (95%) | | | | | |
| --- | --- | --- | --- | --- | --- | --- | --- | --- | --- | --- | --- | --- | --- | --- | --- |
|  | b | Std. b | | | Effect Size | | | | | Lower | | | Upper | | |
| **Model 2^B^ - R = 0.37, R^2^ = 0.13, df = 40, 408 (continued)** |  |  |  |  | |  | | |  | |  | | |  |  |
| 23. Where is your current residence place? Urban area: Yes (1) – No (0) | -0.07 | -0.05 |  | | - | |  | -0.224 | | | | 0.091 | | |  |
| 50. How often do you consume meat in a week? Two to three days a week: Yes (1) – No (0) | -0.07 | -0.05 |  | | - | |  | -0.235 | | | | 0.098 | | |  |
| 18. Do you belong or donate to an organization or charity involved in or concerned with: Conservation of the natural environment: Yes (1) – No (0) | 0.13 | 0.05 |  | | - | |  | -0.183 | | | | 0.437 | | |  |
| 64. How often do you visit a zoo or aquarium? Once a year: Yes (1) – No (0) | -0.08 | -0.05 |  | | - | |  | -0.290 | | | | 0.131 | | |  |
| 47. What is your gross household expenses per month? Above 25 million: Yes (1) – No (0) | -0.39 | -0.04 |  | | - | |  | -1.412 | | | | 0.639 | | |  |
| 51. How often do you consume meat in a week? Four to six days a week: Yes (1) – No (0) | -0.09 | -0.04 |  | | - | |  | -0.318 | | | | 0.144 | | |  |
| 65. How often do you visit a zoo or aquarium? Once every two or more year: Yes (1) – No (0) | -0.07 | -0.05 |  | | - | |  | -0.256 | | | | 0.121 | | |  |
| 53. Do you have pet? Yes (1) – No (0) | -0.04 | -0.03 |  | | - | |  | -0.188 | | | | 0.104 | | |  |
| 26. In what sort of house do you live? Room rent: Yes (1) – No (0) | 0.07 | 0.03 |  | | - | |  | -0.182 | | | | 0.316 | | |  |
| 40. What is your gross household income per month? About the average income in my country: Yes (1) – No (0) | 0.05 | 0.03 |  | | - | |  | -0.152 | | | | 0.259 | | |  |
| 39. What is your gross household income per month? About the minimum income in my country: Yes (1) – No (0) | -0.06 | -0.03 |  | | - | |  | -0.282 | | | | 0.166 | | |  |
| 19. Do you belong or donate to an organization or charity involved in or concerned with: Improving health or human rights: Yes (1) – No (0) | 0.06 | 0.03 |  | | - | |  | -0.206 | | | | 0.323 | | |  |
| 3. Do you have any affiliation to religious organization? Yes (1) – No (0) | -0.03 | -0.02 |  | | - | |  | -0.212 | | | | 0.142 | | |  |
| 44. What is your gross household expenses per month? Five to 10 million: Yes (1) – No (0) | -0.04 | -0.02 |  | | - | |  | -0.279 | | | | 0.193 | | |  |
| 22. Do you have children? Yes (1) – No (0) | 0.04 | 0.03 |  | | - | |  | -0.208 | | | | 0.297 | | |  |
| 21. What is your marriage status? Widow(er): Yes (1) – No (0) | -0.10 | -0.02 |  | | - | |  | -0.690 | | | | 0.485 | | |  |
| 20. What is your marriage status? Married: Yes (1) – No (0) | 0.05 | 0.03 |  | | - | |  | -0.242 | | | | 0.335 | | |  |
| 45. What is your gross household expenses per month? 10 to 15 million: Yes (1) – No (0) | -0.09 | -0.01 |  | | - | |  | -0.934 | | | | 0.753 | | |  |
| 48. What is your gross household expenses per month? Refuse to answer: Yes (1) – No (0) | -0.01 | 0.00 |  | | - | |  | -0.192 | | | | 0.177 | | |  |

Table 6 Main variables and demographical and other determinants to AIS harm animal for environmental issue subscale (continued)

| **Model** | **AIS-Harm animal for environmental issue** | | | | | | | | | CI (95%) | | | | | |
| --- | --- | --- | --- | --- | --- | --- | --- | --- | --- | --- | --- | --- | --- | --- | --- |
|  | b | Std. b | | | Effect Size | | | | | Lower | | | Upper | | |
| **Model 2^B^ - R = 0.37, R^2^ = 0.13, df = 40, 408 (continued)** |  |  |  |  | |  | | |  | |  | | |  |  |
| 13. What is the highest level of schooling you have completed? Senior high: Yes (1) – No (0) | 0.01 | 0.00 |  | | - | |  | -0.315 | | | | 0.331 | | |  |
| 62. How often do you visit a zoo or aquarium? Once a month: Yes (1) – No (0) | 0.01 | 0.00 |  | | - | |  | -0.397 | | | | 0.414 | | |  |
| 38. Is religion important for you? Yes (1) – No (0) | -0.01 | 0.00 |  | | - | |  | -0.629 | | | | 0.607 | | |  |
| 43. What is your gross household income per month? Refuse to answer: Yes (1) – No (0) | 0.00 | 0.00 |  | | - | |  | -0.198 | | | | 0.197 | | |  |

Table 7 Main variables and demographical and other determinants to AIS harm animal for social issue subscale

| **Model** | **AIS-Harm animal for social issue** | | | | | | | | | CI (95%) | | | | | |
| --- | --- | --- | --- | --- | --- | --- | --- | --- | --- | --- | --- | --- | --- | --- | --- |
|  | b | Std. b | | | Effect Size | | | | | Lower | | | Upper | | |
| **Model 1 - R = 0.42, R^2^= 0.17, df = 4, 924** |  |  |  | |  | |  | | |  | | |  | | |
| (Constant) | 3.54 |  | ** | |  | |  | | | 3.099 | | | 3.972 | | |
| EPQ Ideal | -0.09 | -0.15 | ** | | 0.02^C^ | |  | | | -0.135 | | | -0.054 | | |
| EPQ Relative | 0.07 | 0.13 | ** | | 0.01^C^ | |  | | | 0.035 | | | 0.101 | | |
| ROS Personal | -0.39 | -0.27 | ** | | 0.08^C^ | | + | | | -0.479 | | | -0.306 | | |
| ROS Social | 0.17 | 0.23 | ** | | 0.05^C^ | | + | | | 0.127 | | | 0.219 | | |
|  |  |  |  | |  | |  | | |  | | |  | | |
| **Model 2^B^ - R = 0.44, R^2^ = 0.19, df = 40, 408** |  |  |  |  | |  | | |  | |  | | |  |  |
| (Constant) | 3.30 |  | ** |  | |  | | | 2.363 | | 4.245 | | |  |  |
| ROS Personal | -0.34 | -0.24 | ** | | 0.05^C^ | | + | -0.473 | | | | -0.204 | | |  |
| EPQ Ideal | -0.09 | -0.16 | ** | | 0.02^C^ | | + | -0.143 | | | | -0.034 | | |  |
| 1. What is your gender^1^? Female: Yes (1) – No (0) | -0.18 | -0.13 | ** | | 0.23^D^ | | + | -0.273 | | | | -0.078 | | |  |
| 24. In what sort of house do you live? Apartment: Yes (1) – No (0) | 0.59 | 0.09 |  | | - | |  | -0.009 | | | | 1.194 | | |  |
| EPQ Relative | 0.04 | 0.09 |  | | - | |  | -0.003 | | | | 0.089 | | |  |
| 48. What is your gross household expenses per month? Refuse to answer: Yes (1) – No (0) | -0.14 | -0.09 |  | | - | |  | -0.306 | | | | 0.023 | | |  |
| 52. How often do you consume meat in a week? I don't consume meat: Yes (1) – No (0) | 0.23 | 0.08 |  | | - | |  | -0.055 | | | | 0.514 | | |  |
| ROS Social | 0.05 | 0.07 |  | | - | |  | -0.014 | | | | 0.114 | | |  |
| 47. What is your gross household expenses per month? Above 25 million: Yes (1) – No (0) | -0.68 | -0.07 |  | | - | |  | -1.595 | | | | 0.230 | | |  |
| 38. Is religion important for you? Yes (1) – No (0) | 0.40 | 0.07 |  | | - | |  | -0.148 | | | | 0.952 | | |  |
| 25. In what sort of house do you live? Own house: Yes (1) – No (0) | 0.11 | 0.08 |  | | - | |  | -0.040 | | | | 0.257 | | |  |
| 41. What is your gross household income per month? About twice the average income in my country: Yes (1) – No (0) | 0.32 | 0.07 |  | | - | |  | -0.122 | | | | 0.767 | | |  |
| 14. What is the highest level of schooling you have completed? Diploma: Yes (1) – No (0) | 0.31 | 0.07 |  | | - | |  | -0.161 | | | | 0.787 | | |  |
| 40. What is your gross household income per month? About the average income in my country: Yes (1) – No (0) | -0.12 | -0.07 |  | | - | |  | -0.299 | | | | 0.066 | | |  |
| 63. How often do you visit a zoo or aquarium? Once every six month: Yes (1) – No (0) | -0.16 | -0.06 |  | | - | |  | -0.439 | | | | 0.122 | | |  |
| 53. Do you have pet? Yes (1) – No (0) | -0.06 | -0.04 |  | | - | |  | -0.189 | | | | 0.071 | | |  |
| 13. What is the highest level of schooling you have completed? Senior high: Yes (1) – No (0) | -0.12 | -0.05 |  | | - | |  | -0.412 | | | | 0.163 | | |  |
| 43. What is your gross household income per month? Refuse to answer: Yes (1) – No (0) | 0.07 | 0.05 |  | | - | |  | -0.102 | | | | 0.250 | | |  |

*p<.05; **p<.01; ^A^regression using enter method in a stepwise manner; ^B^regression using enter method; ^C^effect-size calculation using eta squared (F^2^); ^D^effect-size calculation using Hedge’s g; *+*small effect size F^2^>=0.02 (or in some cases of categorical dummy variable, using Cohen’s D/Hedges’g >= 0.2); **++**medium effect size F^2^>=0.15 (or in some cases of categorical dummy variable, using cohen’s D/Hedges’g >=0.5); ^1^compared to male respondents.

Table 7 Main variables and demographical and other determinants to AIS harm animal for social issue subscale (continued)

| **Model** | **AIS-Harm animal for social issue** | | | | | | | | | CI (95%) | | | | | |
| --- | --- | --- | --- | --- | --- | --- | --- | --- | --- | --- | --- | --- | --- | --- | --- |
|  | b | Std. b | | | Effect Size | | | | | Lower | | | Upper | | |
| **Model 2^B^ - R = 0.44, R^2^ = 0.19, df = 40, 408 (continued)** |  |  |  |  | |  | | |  | |  | | |  |  |
| 65. How often do you visit a zoo or aquarium? Once every two or more year: Yes (1) – No (0) | -0.07 | -0.05 |  | | - | |  | -0.234 | | | | 0.101 | | |  |
| 44. What is your gross household expenses per month? Five to 10 million: Yes (1) – No (0) | -0.08 | -0.04 |  | | - | |  | -0.285 | | | | 0.135 | | |  |
| 19. Do you belong or donate to an organization or charity involved in or concerned with: Improving health or human rights: Yes (1) – No (0) | 0.08 | 0.04 |  | | - | |  | -0.151 | | | | 0.319 | | |  |
| 42. What is your gross household income per month? More than twice the average income in my country: Yes (1) – No (0) | 0.27 | 0.03 |  | | - | |  | -0.507 | | | | 1.050 | | |  |
| 21. What is your marriage status? Widow(er): Yes (1) – No (0) | -0.17 | -0.04 |  | | - | |  | -0.696 | | | | 0.350 | | |  |
| 3. Do you have any affiliation to religious organization? Yes (1) – No (0) | -0.05 | -0.04 |  | | - | |  | -0.209 | | | | 0.106 | | |  |
| 15. What is the highest level of schooling you have completed? Bachelor: Yes (1) – No (0) | 0.05 | 0.03 |  | | - | |  | -0.132 | | | | 0.236 | | |  |
| 2. What is your age? | 0.00 | 0.03 |  | | - | |  | -0.006 | | | | 0.011 | | |  |
| 45. What is your gross household expenses per month? 10 to 15 million: Yes (1) – No (0) | -0.20 | -0.02 |  | | - | |  | -0.951 | | | | 0.550 | | |  |
| 18. Do you belong or donate to an organization or charity involved in or concerned with: Conservation of the natural environment: Yes (1) – No (0) | 0.07 | 0.03 |  | | - | |  | -0.203 | | | | 0.349 | | |  |
| 17. Do you belong or donate to an organization or charity involved in or concerned with: Animal sector: Yes (1) – No (0) | -0.11 | -0.03 |  | | - | |  | -0.528 | | | | 0.312 | | |  |
| 49. How often do you consume meat in a week? Everyday: Yes (1) – No (0) | -0.05 | -0.03 |  | | - | |  | -0.250 | | | | 0.149 | | |  |
| 64. How often do you visit a zoo or aquarium? Once a year: Yes (1) – No (0) | -0.05 | -0.03 |  | | - | |  | -0.234 | | | | 0.141 | | |  |
| 50. How often do you consume meat in a week? Two to three days a week: Yes (1) – No (0) | -0.03 | -0.02 |  | | - | |  | -0.182 | | | | 0.115 | | |  |
| 51. How often do you consume meat in a week? Four to six days a week: Yes (1) – No (0) | 0.05 | 0.02 |  | | - | |  | -0.160 | | | | 0.250 | | |  |
| 39. What is your gross household income per month? About the minimum income in my country: Yes (1) – No (0) | 0.04 | 0.02 |  | | - | |  | -0.159 | | | | 0.240 | | |  |
| 62. How often do you visit a zoo or aquarium? Once a month: Yes (1) – No (0) | 0.03 | 0.01 |  | | - | |  | -0.329 | | | | 0.392 | | |  |

Table 7 Main variables and demographical and other determinants to AIS harm animal for social issue subscale (continued)

| **Model** | **AIS-Harm animal for social issue** | | | | | | | | | CI (95%) | | | | | |
| --- | --- | --- | --- | --- | --- | --- | --- | --- | --- | --- | --- | --- | --- | --- | --- |
|  | b | Std. b | | | Effect Size | | | | | Lower | | | Upper | | |
| **Model 2^B^ - R = 0.44, R^2^ = 0.19, df = 40, 408 (continued)** |  |  |  |  | |  | | |  | |  | | |  |  |
| 23. Where is your current residence place? Urban area: Yes (1) – No (0) | -0.01 | -0.01 |  | | - | |  | -0.148 | | | | 0.133 | | |  |
| 22. Do you have children? Yes (1) – No (0) | 0.01 | 0.01 |  | | - | |  | -0.213 | | | | 0.236 | | |  |
| 27. Do you have your own backyard? Yes (1) – No (0) | 0.00 | 0.00 |  | | - | |  | -0.133 | | | | 0.128 | | |  |
| 20. What is your marriage status? Married: Yes (1) – No (0) | 0.00 | 0.00 |  | | - | |  | -0.254 | | | | 0.259 | | |  |
| 26. In what sort of house do you live? Room rent: Yes (1) – No (0) | 0.00 | 0.00 |  | | - | |  | -0.220 | | | | 0.223 | | |  |
