# Supplementary material for: Do religious beliefs influence concerns for animal welfare? the role of religious orientation and ethical ideologies in attitudes toward animal protection amongst Muslim teachers and school staff in East Java, Indonesia
Source: PLoS One. 2021 Jul 16;16(7):e0254880. doi: 10.1371/journal.pone.0254880 (PMC8284611; doi:10.1371/journal.pone.0254880)
Supplement: Attachment — Submitted filename: review_round2.pdf [file pone.0254880.s006.pdf]

Do religious beliefs influence concerns for animal welfare? The role of religious orientation and ethical ideologies in attitudes towards animal protection against Muslim teachers and school staff in East Java, Indonesia

Broadly speaking, I believe the authors have responded appropriately to most of my comments raised in the first round of review. I think the paper is much clearer (I'd like to hope the authors agree). I very much see the value in adding this data to the literature, and I am particularly appreciative of the insightful context added to the paper with regards to the sampling of educators specifically in Indonesia – this was very compelling. Moreover, I think the discussion is clear and useful. What follows are a list of remaining concerns/comments/suggestions:

1. Might the "influence" in the title be too causally strong given the cross-sectional correlational design of the study – perhaps "Are religious beliefs associated with…" is more appropriate (other options of course acceptable).
2. The one response that I disagree with is author response point 5 re: effect size estimates and confidence intervals. R-squared is *not* sufficient information in reporting a regression model. Future researchers could make good use of the estimated regression coefficients and their associated confidence intervals, in for example meta-analyses and/or sample size planning of future work. Generally, I am of the view that presenting an effect size estimate without a confidence interval is only doing half the work – the interval is much more interesting than the point estimate anyway. So, confidence intervals should be included on *all* effect sizes, including correlations, and regression coefficients.
3. In relation to #2, there is still not enough being said about the magnitude of the associations between variables – in particular with regards to the results of the regression in Table 7. I believe the regression coeffecients presented here for the focal variables are unstandardized, but presenting the standardized coeffecients would be helpful for communicating your results. At the very least, more needs to be said about what a regression coefficient of .05 and .12 (your two focal results) means in terms of your scales – what is the magnitude of the effect. In the discussion, an effect size of .09 is noted as being showing "little to no effect". But, there's no such discussion when the regression coefficients of focal predictors are of a relative size (That being said I know these aren't the standardized effects being presented in Table 7, so it's possible these effects are bigger than they seem – but that information and the interpretation there of must be presented to readers). If anything, the estimates of those two regression coeffecients – are generally the entire point of your paper – so more should be done to contextualize them, and demonstrate that they are meaningful (i.e., not just tiny effects in a large sample).
4. The introductory section is quite vague about the direction of reviewed effects – and given the mixed results generally reported, it's very difficult to track which associations being reviewed are positive or negative. Some clarification throughout will go a long way in helping readers follow the logic here and really specify the reviewed literature in a way that would benefit the paper (e.g., every instance of reporting an association from the literature, a simple "positive" or "negative" will help).
5. LINE 142 = The use of "proven" seems inappropriate

6. Methods/Materials section has some new text with the use of the first person, perhaps should edited to maintain consistency throughout the paper.
7. The formatting of all tables have been skewed (potentially just by the editorial system, but many of the tables are very hard to read as many things are mis-aligned). And there seems to be some residual columns/rows after Table 7, not clear if those were meant to be examined or not (probably just a formatting issue).
8. The AAS scale is mentioned several times in the results, but it was supposed to be removed from the analyses, so authors should double check whether they meant AIS instead or if these are lingering results from a previous version.
9. LINE 430 = seems to misreport the positive correlation between idealism and relativism as a negative relationship between extrinsic religiosity and “ethical ideologies”? Not sure what is meant (says to see section 3.6 but these details are not provided there)
10. The interpretation of the results presented in Table 7 do not align with the numbers in the table (unless relativism has been reverse coded, which is not clearly detailed prior to this point?). There is no relationship between idealism and AIS in this regression (b = -.02) so it doesn’t follow to say “While the result shows idealism behaves according to the hypothesized relation (higher the idealism leads to a lower overall acceptability for harming animals”; Lines 453). The reporting in section 3.5 seems accurate.
11. A correlation table of all focal variables would be helpful (ROS and subscales, AIS, and ethical ideologies).
12. There is mention that in Table 7, model 2, the effects of idealism/relativism were not significant, but these results have been omitted from the Table (“-“) – so impossible to verify. Generally speaking, “backwards selection” is not a good analytical tool, and generally should not be used (e.g., https://journalofbigdata.springeropen.com/articles/10.1186/s40537-018-0143-6). Better to leave the effects of non-significant variables in the regression model.
13. Line 475 seems to contradict itself, there’s no correlation between idealism and social religious orientation, but the authors accept the hypothesis that there is one?
14. Line 488 = mention of figure 2 but there is no figure 2.
