# Supplementary material for: Do religious beliefs influence concerns for animal welfare? the role of religious orientation and ethical ideologies in attitudes toward animal protection amongst Muslim teachers and school staff in East Java, Indonesia
Source: PLoS One. 2021 Jul 16;16(7):e0254880. doi: 10.1371/journal.pone.0254880 (PMC8284611; doi:10.1371/journal.pone.0254880)
Supplement: Attachment — Submitted filename: Response to Reviewers.docx [file pone.0254880.s005.docx]

**Response to Reviewers**

Dear Reviewers,

Thank you for your review and comments on our manuscript addressing critical points in order for us to make it better and more concise. We are very glad to have this review and honored that you provide your valuable time for reviewing this in such a swift manner.

In the tables below we indicated how we revised our manuscript in detail.

Thank you very much in advance for your consideration.

With kind regards,

Dexon Pasaribu, Pim Martens and Bagus Takwin

| 1 | I think this paper requires lots of additional work before it will be suitable for publication - and especially so (but not exclusively) with regards to providing more detail into how the data was handled. That being said, it’s important to reiterate that this is likely very valuable data - the authors will just have to do more to convince readers that they have properly tested their focal hypotheses. My primary concern is that given the manuscript in its current state, I would not be able to (1) replicate the study should I be interested in doing so as their methods are underspecified and (2) even if I had access to their already collected data, I’m not convinced that I would be able to reproduce the analyses from the details presented in this paper. |
| --- | --- |
|  | Response:  Dear Reviewer,  We sincerely grateful to your feedback and insight regarding data handling procedures in our articles. Indeed, after heeding your advice and coordinating with our university board of ethics in order to conform to EU regulation for submitting data, we can now share full access to both our raw and SPSS data.  Also, we added more information on procedures of handling our data in the Material and methods section, including supporting information which covers results of normal distribution check, missing case analysis and replacement, and also, factor Analysis results. |

| 2 | The authors never provide details as to which items were dropped - nor do they inform readers as to exactly how reliability was improved by dropping these items. Moreover, might it be the case that the full set of items are simply better reduced to multiple factors as opposed to one? The results of an EFA on all items (either in the main text or a supplemental materials section) would go a long way to demonstrating the decisions made in creating one of the focal outcome variables in this study. The same is true for the Ethical Position Questionnaire, where 5 items were dropped from the full scale. |
| --- | --- |
|  | Response:  Dear Reviewer,  We added more information for each of the questionnaire in the 3.1 instrument validity section. In that section, we explained more about the Principal Axis factor analysis results for each of the questionnaire, the reliability, and the decision to drop the items we found not suitable to be included in the analysis. We also add supporting documents S3 Missing case analysis, Factor Analysis and Reliability to explain the normality check, factor analysis, and reliability analysis which would lead to our decision to drop some items.  In relation to your other feedbacks of normal distribution, we decided to use Kim’s (2013) explanation which argues that the tendency of large samples producing inflated z in consideration to large samples will usually produce a very small standard error for both skewness and kurtosis. Therefore, using skewness and kurtosis reference values for N more than 300, the present study removed items with kurtosis value outside the range between -7 to 7, or skew value outside the range between -2 to 2 (Kim, 2013). After this analysis, we dropped four items in the ethical Idealism scale, while the remaining were still fit well within the normality boundary.  We also would like to mention specifically that after having above analysis, we removed AAS questionnaire in the revision. After principal axis factoring factor analysis, previously intended as cross-validation for the AIS, the 20-item Likert-like scale AAS failed to provide a stable unidimensional construct as it was in its original psychometric properties (see S3 Missing case analysis, Factor Analysis and Reliability; Table 35-49). Alpha’s reliability also showed small to moderate coefficients for each of the resulting factors. Thus, AAS was removed from the analysis. |

| 3 | In discussing their sample, the authors say that 78 respondents were dropped because of missing data; but that their data was replaced using “linear trends method”. Their final sample size is 929 (=1007 minus 78) and thus it doesn’t seem like any data was replaced? The authors should be more clear about the imputation process they used, if they used one. Minor comment, including an age range would be helpful. |
| --- | --- |
|  | Response:  Dear Reviewer,  Thank you for addressing this. We added more information in section 3.2 and supporting information (S3 Missing case analysis, Factor Analysis and Reliability) where we explained the the decision to remove 78 from total 1007 participants, and provided table of missing case analysis for the remaining 929 participants as to show in how many items we made imputation for the listwise missing response. |

| 4 | The authors state that some of their data was “translated into a normal distribution using either outlier removal, log-10 or square-root transformation” (line 239). But yet, they never tell readers which variables have been transformed. More detailed information about the spread of responses (histograms or density plots, for example) would go a long way in justifying these decisions to transform the data. But more importantly, not knowing which variables were transformed makes the focal tests of the hypotheses presented in the regression impossible to interpret - as readers have no insight into how the variables were entered into the model. |
| --- | --- |
|  | Response:  Dear Reviewer,  Thank you for addressing this issue. As we learn more on your other feedback to include effect-size analysis to avoid depending solely to statistical significance in a big data, in the revision, we decided to use Kim’s (2013) explanation which argues that the tendency of large samples producing inflated z in consideration to large samples will usually produce a very small standard error for both skewness and kurtosis. Therefore, using skewness and kurtosis reference values for N more than 300, the present study removed items with kurtosis value outside the range between -7 to 7, or skew value outside the range between -2 to 2 (Kim, 2013).  We also applied the same treatment to the total score of each of the questionnaires and we found all the distribution to be normal. Thus, using this reference, we leave each scale as it is considering they are still within the normal distribution boundary. |

| 5 | On that front, are the regression coefficients presented in Table 3 standardized? If not, were the variables centered (which would be necessary to make sense of any of the coeffecients given the interactions included in the models).  Moreover, the authors focus exclusively on statistical significance and don’t interpret their effect sizes - most of which seem really small (although again, impossible to interpret given the lack of information about how the variables were coded and entered into the model). I’d suggest that confidence intervals should be included around all effect size estimates in the manuscript (including correlation tables, regression tables, etc.) - and more should be done to interpret the magnitude of associations rather than just their statistical significance. In a large enough sample such of this one, most non-zero effects will be significant - but it is up to the authors to convince readers that these are in any way meaningful associations. |
| --- | --- |
|  | Response:  Dear Reviewer,  Thank you for your input in this specific issue. Yes, in analyzing interactions, we always use standard z of the Xs. Otherwise it is meaningless.  In compliance to your input, we added effect size analysis in all the demographic dummy variables. However, we did not put any effect size analysis to those variables which already in interval scale (namely personal and social religious orientation. EPQ idealism, relativism and age) because it is only one sample and R squared is already sufficient. |

| 6 | As the analysis plan was not pre-registered, I expect to see more than one regression model per outcome variable. I’d suggest one regression table per outcome measure - with several models included for each. For instance, an informative table might present the results as follows: Model 1 (main predictor variables), Model 2 (main predictors + demographic controls), Model 3 (main predictors + demographic controls + interaction variables). This way, any variation in effect size estimates related to inclusion/exclusion of other covariates can be made explicitly clear to readers; and provides a good test of how robust effects are to addition of controls. One way to limit the number of tables here would be to move sub-scale analyses on the Animal Issues Scale to a supplemental materials section. Given the high reliability of scores across the sub-scales (.93) - I’m not convinced that authors are gaining all that much from repeatedly testing their hypothesis at the sub-scale level. Moreover, this would really help streamline the results section which is, at the moment, quite dense.  In the regressions, the authors test for the 3-way interaction between forms of religiosity, and the interaction between idealism/relativism. However, neither of these interactions seem to follow directly from the predictions laid out in the introductory sections. Thus, I think it would be important to show models with and without these interactions included. Or, if there are no strong predictions to be made about these interactions - simply omit them for now (or move tests of them to a supplemental materials section). In following up on these interactions, the authors present an ANOVA assessing whether outcome measures differ based on level of idealism/relativism. However, the results of the regressions where this interaction was consistently not significant would indicate that there is no interaction between these variables across outcomes - and thus retesting this interaction in the ANOVA is, I think, unwarranted. I would advise against breaking up the data in this way. This is especially true given that the authors don’t provide details as to how their absolutist/situationist/exceptionist/subjectivist categories were created - and thus I think these additional analyses do little to elaborate on what is already being tested in the regression. Lastly, in plotting these interactions - the full scale of response options should be presented on the y-axis, as the zoomed in scales presented here make a very tiny effect seem bigger than it is. Moreover, it would be helpful if raw data points were also plotted and not just the lines. |
| --- | --- |
|  | Response:  Dear Reviewers,  Thank you for your input for the regression analysis and as to why this study benefit the most from it. We added more information in the Material and method section (specifically in the statistical analysis), where we registered and argued in favor of multiple regression method. Previous studies mostly used Anova considering that they coded the EPQ into four variables depending on the high and low of each of the idealism and relativism subscales (namely situationist, subjectivist, absolutist and exceptionists) (Figure 1).    Figure 1 Ethical positions according idealism and relativism  Contrary and supplementary for previous study (B Su & Martens, 2017; Bingtao Su & Martens, 2018) we decided to retain the interval properties from the total score of ethical idealism and relativism to provide richer and complete data without any reduction to categorical properties. Thus, putting another section reverting into categorical models and analyzed the data using ANOVA and also examining three-way interaction in the regression models are some attempts to refer to our previous study.  However, as you have argued, we realized that it only added some noises and blurred the focal point of this study. Therefore, in the revision, we removed the anova analysis. Also, after having your suggestion to put three models (main predictors, main predictors and demographical variables, and main predictors with demographical and interaction), we found that only the first two models provide fruitful results and thus we removed interaction analysis in our revision. |

| 7 | As mentioned before, the results section is already quite dense. One way I see to really alleviate some pressure on readers is to move all the writing about demographic covariates to a supplemental materials section. Although surely valuable, these are not the focal predictions of the paper and including them in the main results sections both detracts from the tests of the focal hypotheses about religion and makes the results section harder to digest. This would helpfully reduce the length of the discussion section as well. |
| --- | --- |
|  | Response:  Dear Reviewer,  Thank you for the valuable input. As suggested, we omitted those details which not specifically related to the focal point and put them as supporting material (in ‘S4 AIS subscales multiple regression results’). |

| 8 | I think the introduction would benefit greatly from some further specification of the reviewed literature. Indeed, the authors mention that much of the discrepancy or mixed results might result from different operationalizations of religion/religiosity in different samples. But yet, they do little to highlight differences in methodologies in reviewing the literature - and importantly, it is rarely the case that the “religion” being investigated in the literature is specifically mentioned - making it very hard to track what has already been done in this research area. It’s important to not generalize across religious traditions, and would be very helpful for readers if the authors were more specific in their review. I think the clarity in the section discussing ethical idealism v ethical relativism could serve as a good model for how the rest of the introduction’s review should be structured. Lastly, I would caution quite strongly against the use of “proven” in describing the results of any empirical research, and there are several instances when causal language is used to describe the results of correlational research. |
| --- | --- |
|  | Response:  Dear Reviewer,  Thank you for your inputs for this specific issue. In this study we are very much aware to avoid dwelling much into specifics of a particular religion or religious content and such because in our opinion, it may misplace the focus of the study more to religious or theological studies. However, we do realize that we need to start from a focal point and thus, we choose Allport’s religious orientation in examining the religion properties we’re intending to examine. As such, we started to focus on differing aspect of previous study and put emphasis to the differing operationalization of religion used in those studies. We argue that different operationalization of religion will generally bring different focus and different methodologies which in turn, will bring different (and often mixed) results. With this in mind, we carefully shifted and selected our focus to Allport’s religious orientation as the most suited general construct for our study at the time being.  One important concept we want to highlight is more to one’s own interpretation and internalization of their own religion in terms of personal and social motivation for practicing their religion. Interestingly enough, one interpretation that can be brought from our results is how close the extrinsic social religious orientation may resembles with social identity theory commonly used in explaining religious conflicts, which adds more complexity in indexing the vast and various method for religion and religiousness properties utilization. We are currently also thinking about how differing typical motivation will interacts with different religion scripture interpretation which is another potential topic to investigate. Thus, at the time being, we decide to put the introduction as it is in the revision, and view that it is most fruitful to discuss this in another paper. If you found this issue is not principally damaging our article, we hope that you can understand our decision not to change it. But we do think about this for our next article. Thank you. |

| 9 | All in all, behind this paper there is good and useful data and with some more work it will be a really nice paper. I was surprised that there was not more discussion of the fact that this data was from teachers in the discussion, and would be curious to see more reflection on that from the authors in a revision. |
| --- | --- |
|  | Response:  Dear Reviewer,  Thank you for addressing this issue. We added information of why we purposefully selected teachers and educators as our participants in the introduction. |

**References**

Kim, H.-Y. (2013). Statistical notes for clinical researchers: assessing normal distribution (2) using skewness and kurtosis. *Restorative Dentistry & Endodontics*, *38*(1), 52. https://doi.org/10.5395/rde.2013.38.1.52

Su, B, & Martens, P. (2017). Public attitudes toward animals and the influential factors in contemporary China. *Animal Welfare*, *26*(2), 239–247. https://doi.org/10.7120/09627286.26.2.239

Su, Bingtao, & Martens, P. (2018). How Ethical Ideologies Relate to Public Attitudes toward Animals: The Dutch Case. *Anthrozoos*, *31*(2), 179–194. https://doi.org/10.1007/s00210-018-1496-2
