# Supplementary material for: Do religious beliefs influence concerns for animal welfare? the role of religious orientation and ethical ideologies in attitudes toward animal protection amongst Muslim teachers and school staff in East Java, Indonesia
Source: PLoS One. 2021 Jul 16;16(7):e0254880. doi: 10.1371/journal.pone.0254880 (PMC8284611; doi:10.1371/journal.pone.0254880)
Supplement: Attachment — Submitted filename: 2ndroundreview_responsetoreviewer.docx [file pone.0254880.s007.docx]

**Response to Reviewers**

Dear Reviewers,

Thank you for your 2^nd^ round review and comments for our manuscript. We are very glad and thankful for having all of these inputs. Also, thank you for providing your valuable time to review our manuscript in a very swift manner, despite our current pandemic situation.

Below, we indicate how we revise our manuscript in detail. Thank you very much in advance for your kind consideration and attention.

With kind regards,

Dexon Pasaribu, Pim Martens and Bagus Takwin

| 1 | Might the “influence” in the title be too causally strong given the cross-sectional correlational design of the study – perhaps “Are religious beliefs associated with…” is more appropriate (other options of course acceptable). |
| --- | --- |
|  | Response:  Dear Reviewers,  Thank you for the correction. We strongly agree and revise the title considering that indeed, this study uses correlational design and therefore, no cause-effect relation can be concluded. |

| 2 | The one response that I disagree with is author response point 5 re: effect size estimates and confidence intervals. R-squared is not sufficient information in reporting a regression model. Future researchers could make good use of the estimated regression coefficients and their associated confidence intervals, in for example meta-analyses and/or sample size planning of future work. Generally, I am of the view that presenting an effect size estimate without a confidence interval is only doing half the work – the interval is much more interesting than the point estimate anyway. So, confidence intervals should be included on all effect sizes, including correlations, and regression coefficients. |
| --- | --- |
|  | Response:  Dear Reviewers,  After careful thought, we decide to follow your suggestion. We revise the way we present the result tables and put effect-size both in the Pearson’s correlation (table 6) and regression results (table 7). For the effect-size of each regression components, we use both eta-squared (F2) method (for interval variables) and Hedge’s g (for categorical dummy variable). Our reasoning for the latter is because instead of Cohen’s D, Hedge’s g considers any difference of sample size (N) and SD between the compared groups. As for the explanation of the results, we put more details by integrating the context of confidence interval and effect-size. |

| 3 | In relation to #2, there is still not enough being said about the magnitude of the associations between variables – in particular with regards to the results of the regression in Table 7. I believe the regression coeffecients presented here for the focal variables are unstandardized, but presenting the standardized coeffecients would be helpful for communicating your results. At the very least, more needs to be said about what a regression coefficient of .05 and .12 (your two focal results) means in terms of your scales – what is the magnitude of the effect. In the discussion, an effect size of .09 is noted as being showing “little to no effect”. But, there’s no such discussion when the regression coefficients of focal predictors are of a relative size (That being said I know these aren’t the standardized effects being presented in Table 7, so it’s possible these effects are bigger than they seem – but that information and the interpretation there of must be presented to readers).  If anything, the estimates of those two regression coeffecients – are generally the entire point of your paper – so more should be done to contextualize them, and demonstrate that they are meaningful (i.e., not just tiny effects in a large sample). |
| --- | --- |
|  | Response:  Dear Reviewers,  As requested, we put additional column for standardized beta into the table. As we mentioned in our answer for question #2, we also put more context (e.g. confidence intervals) when explaining the results, e.g. line 486 to 508:  *For the first model (Table 7), we find only partial evidence to support the hypothesis. The results show that only for ethical relativism we can accept the hypothesis, where we find higher relativism is more likely lead to a higher overall acceptability for harming animals (AIS) (b=0.05, p<0.01). This means that when holding all other variables constant, one point increase in relativism is likely to increase 0.05 point of AIS score. Moreover, it is important to mention that from the effect-size aspect, relativism has little to no effect toward AIS score (F2 <= 0.02). Through the confidence interval, if we were to retake the regression for total of 20 random trials, taking samples of the same size from the same population, we can be confident that for 19 out of total 20 trials (95% of the time), an increase of 1 unit of relativism will be more likely to increase AIS between 0.02 to 0.07 point. Thus despite accepting the hypothesis for every relation with relativism, this study advises to take caution to limit the interpretation because of the near non-existent effect-size. In short, in the first model, idealism has no relation to overall acceptability for harming animals (AIS), and relativism significantly relates to a higher AIS (b=0.05, p<0.01). However, the confidence interval and effect-size indicate a small to no effect, suggesting that relativism relation to AIS is not as strong as its relation with IP, ES, and some of demographical or other determinants.*  *An important addition from this study is when observing the second regression model, whereby all main variables along with demographics and other determinants are taken together as well as independently. From the second model, this study shows no significant relation between AIS with both relativism and idealism.*  *An important addition from this study is when observing the second regression model, whereby all main variables along with demographics and other determinants are taken together as well as independently. From the second model, this study shows no significant relation between AIS with both relativism and idealism.* |

| 4 | The introductory section is quite vague about the direction of reviewed effects – and given the mixed results generally reported, it’s very difficult to track which associations being reviewed are positive or negative. Some clarification throughout will go a long way in helping readers follow the logic here and really specify the reviewed literature in a way that would benefit the paper (e.g., every instance of reporting an association from the literature, a simple “positive” or “negative” will help). |
| --- | --- |
|  | Response:  Dear Reviewers,  Thank you for pointing this out. We put more explanation in the introduction. Hopefully that these additions will be sufficient (line 56 to 94):  *For the relation between ethical ideologies and public attitudes toward animals, previous studies showed mixed results. It was found that ethical idealism relates positively to a higher concern for animal use [16]. Through their research about the effectiveness of materials designed to sway public opinion about biomedical research using animals, Herzog & Nickell [17] would later add that compared to males and those low in ethical idealism, females and subjects high in moral idealism rate higher effectiveness to those research materials and advertising that reject animal use in biomedical research (anti-animal research materials) (p. 9). Also similar, Wuensch and Poteat [25] concluded that support for animal research associate negatively with idealism but positively with relativism. More recent studies by Su & Martens [18, 26] also confirmed these results, showing that higher idealism scorers are more likely to have a more positive attitude toward animals and a lower acceptability for harming animals. However Su & Martens [18, 26] slightly deviate from older studies [16, 17] whereby they find that high scorers of ethical relativism are more likely to have a more negative attitude toward animals only in China [18], but not in their Dutch sample [20]. Su & Martens argued that the differences between both samples might stem from the difference between being a developed and developing country, respectivily [18]. On the opposite spectrum, Wuensch, Jenkins, & Poteat [19] found that among nonidealists there is a significant positive relationship between misanthropy and support for animal rights, while among idealists the regression line is flat. They argued that misanthropic nonidealists discount the value of benefits to humankind (or may even consider them of negative value), and thus cannot justify animal use to benefit humankind [19].*  *For the relation between ethical ideologies and religion, previous studies’ results were much more consistent and straightforward. Cornwell et al. [23] found that religion has some effect on ethical positions. Austrian Christians are significantly less idealistic and relativistic than all other religions, even with other Christians from the United States and Britain [23]. They argued that there are some ethical convergence between religions [23]. In another study, Barnett, Bass, and Brown [21] concluded that religiosity correlates positively with a non-relativist ethical ideology. Closely similar with them, Watson, Morris, Hood, Milliron, & Stutz [24] argued that religious intrinsicness or religious intrinsic personal orientation is associated with the idealism and antirelativism of an absolutist ethical position. They argued that intrinsic commitments to religion may simply mean that certain beliefs are absolutely nonnegotiable [24] (p. 5). In Forsyth’s [27] terms, this absolutistic way of thinking type is the result when people strongly believe that moral decision should be guided by an universal governing principle (low relativism) rather than by personal or situational analysis (high relativism) while also convinced that ethical behavior will always lead to positive consequences. Thus, combining results from these studies, the present study aims to examine the relationship between ethical ideologies and religious orientation, and to explore how both religious orientation and ethical ideology interact and relate with participants’ acceptability towards harming animals.* |

| 5 | LINE 142 = The use of “proven” seems inappropriate |
| --- | --- |
|  | Response:  Dear Reviewer,  Thank your input. As such, we change the wording to a more accurate description depicting previous research results in the relevant field. |

| 6 | Methods/Materials section has some new text with the use of the first person, perhaps should edited to maintain consistency throughout the paper. |
| --- | --- |
|  | Response:  Dear Reviewers,  The first-person style in this paragraph was asked specifically by PLOS One in the early phase of submitting the article. At that time, we already wrote the ethic statement into PLOS One website submission form when submitting. However, PLOS One replied and asked us to also write these statements into the method section of the article.  We will revise this section as your suggestion, and will consult with PLOS One for their approval of the revision.   |

| 7 | The formatting of all tables have been skewed (potentially just by the editorial system, but many of the tables are very hard to read as many things are misaligned). And there seems to be some residual columns/rows after Table 7, not clear if those were meant to be examined or not (probably just a formatting issue). |
| --- | --- |
|  | Response:  Dear Reviewer,  We have consulted PLOS One about the irregularities of the previously submitted manuscript. In the output of the submitted article (in pdf) automatically generated by PLOS one website, we can clearly see that there are some disrupted characters out of place. This problem may indeed because of the formatting glitch resulted from the PLOS One website’s automatic pdf generation output.  Therefore, we will pay extra attention to this in submitting our 2^nd^ round revised manuscript. |

| 8 | The AAS scale is mentioned several times in the results, but it was supposed to be removed from the analyses, so authors should double check whether they meant AIS instead or if these are lingering results from a previous version. |
| --- | --- |
|  | Response:  Dear Reviewer,  Thank you for the correction. Indeed this was a mistake. After following the 1^st^ round reviewers’ suggestion of having factor analysis, we found AAS was not a reliable factor and therefore deleted it from analysis. However, there is one sentence we missed and will remove this from the sentence.  On the other hand, we also feel the need to mention that our original intent was to utilize AAS, and explain why we delete AAS from the study after observing the factor analysis result. Therefore, the AAS is still mentioned and explained only in the method section (only in line 313 to 320). |

| 9 | LINE 430 = seems to misreport the positive correlation between idealism and relativism as a negative relationship between extrinsic religiosity and “ethical ideologies”? Not sure what is meant (says to see section 3.6 but these details are not provided there) |
| --- | --- |
|  | Response:  Dear Reviewers,  We intend to shortly mention the correlation between extrinsic social religious correlation with both idealism and relativism (ethical ideologies) in this section. On the other hand, these relation also specifically inform the results of the working hypothesis that a higher extrinsic social religious orientation (ES) correlates to lower idealism (I), higher relativism (R), and a higher acceptability for harming animals (AIS). Therefore, in order to avoid unnecessary repetition and to represent the findings in an organized manner, we move the explanation to section 3.6, which specifically explaining this specific working hypothesis.  However we mistakenly put the wrong number of 0.35 (that supposedly the correlation between idealism and relativism) when it should be 0.15 (correlation coefficient between ES and relativism). Thus we revise this paragraph (line 460 to 468) to:  *The hypothesis presented in this section is that higher personal religious orientation relates to a higher idealism and a lower relativism. Table 6 provides the correlation matrix for the studied variables. We find positive relationship between idealism with personal religious orientation (IP) (r[927]=0.21, p<0.01). However, there is no significant relationship between relativism with IP (r[927]=0.000, p>0.05), and therefore, while the hypothesis is rejected by every relation with relativism, it is accepted in predicting the relationship between idealism with IP. Lastly, the correlation between extrinsic social religious orientation and idealism (r[927]=-0.02, p>0.05) and relativism (r[927]=0.15, p<0.01) is reported with a more detail in another section (see section 3.6).* |

| 10 | The interpretation of the results presented in Table 7 do not align with the numbers in the table (unless relativism has been reverse coded, which is not clearly detailed prior to this point?). There is no relationship between idealism and AIS in this regression (b = -.02) so it doesn’t follow to say “While the result shows idealism behaves according to the hypothesized relation (higher the idealism leads to a lower overall acceptability for harming animals”; Lines 453). The reporting in section 3.5 seems accurate. |
| --- | --- |
|  | Response:  Dear Reviewer,  Before answering, for clarity, we want to inform that Idealism, Relativism, IP, ES and AIS are not reverse coded. However, in our attempt to shorten the sentence and word count in the article, we introduced negative abbreviation for relativism (R) as anti-relativism (-R), as a way of saying low scores of relativism. For the relation between relativism (R) and acceptability for harming animals (AIS) in table 7, the correct interpretation is “the higher the relativism score, the more likely the higher the participant’s acceptability for harming animals (b=0.05, *p*<0.01)”. In the article we decide to interpret this in reverse as “anti-relativism (-R) relates to lower acceptability (b=0.05, *p*<0.01),” as an effort to lessen any difficulty in relating it to idealism and our working hypothesis for the three variables.  However, we now understand that it may only add more confusion to the reader. Therefore, we revise this to closely and consistently align with the result in table 7:  Line 472 to 478  *The hypothesis presented in this section is that higher acceptability for harming animals (AIS) relates to a lower idealism and a higher relativism. There are two models developed and analysed using the multiple regression method (Table 7). The first model analyses the four main variables relation to AIS, namely idealism, relativism, intrinsic personal and extrinsic social religious orientation. The second model investigates all four main variables by taking all potential demographic and other determinants into account with the equation.*  Line 486 to 508 (see response for review #3), and, in accordance to this we also made changes in the paragraph where we state our working hypothesis (line 175-179):  *As it was found in previous studies [21, 24], as the first working hypothesis, we predict that intrinsic personal religious orientations will have a positive correlation with ethical idealism and a negative correlation with relativism. Also taking the consistent results from various studies [16, 18, 20, 25], as our second working hypothesis we predict that higher acceptability for harming animals relates to a lower ethical idealism and a higher relativism.*  Secondly, we admit that the sentence below:  *“While the result shows idealism behaves according to the hypothesized relation (higher the idealism leads to a lower overall acceptability for harming animals”*  maybe confusing. At first we only want to describe that despite not significant, the consistent negative correlation between AIS and idealism that were found in various studies (Galvin & Herzog, 1992; B Su & Martens, 2017; Bingtao Su & Martens, 2018; Wuensch & Poteat, 1998) is also captured in our study. However, as it is not statistically significant, we refrain from reporting it and delete the sentence and also revise all other sentence related to this. |

| 11 | A correlation table of all focal variables would be helpful (ROS and subscales, AIS, and ethical ideologies). |
| --- | --- |
|  | Response:  Dear Reviewers,  As suggested, we revise the Pearson’s r results (Table 6) to include all the focal variable of this study. |

| 12 | There is mention that in Table 7, model 2, the effects of idealism/relativism were not significant, but these results have been omitted from the Table (“-“) – so impossible to verify. Generally speaking, “backwards selection” is not a good analytical tool, and generally should not be used (e.g., https://journalofbigdata.springeropen.com/articles/10.1186/s40537-018-0143-6). Better to leave the effects of non-significant variables in the regression model. |
| --- | --- |
|  | Response:  Dear Reviewers,  Thank you for your input and for the referred literature. After careful consideration, we decide to follow your suggestion and use enter method for the linear regression (Smith, 2018; Whittingham, Stephens, Bradbury, & Freckleton, 2006). In incorporating your suggestion, we made revisions in the statistical method (line 374 to 383):  *This study uses two models of multiple regression. The first model only investigates the main variables, while the second model takes all main variables with the demographic and other important determinants. For both of the regression models, this study avoids stepwise method in considering that stepwise estimates are not invariant to inconsequential linear transformation. [81]. Rather, we follow Whittingham, Stephens, Bradbury, and Freckleton’s [82] suggestion to use a full model including all of the effects (enter method) for the second regression model, where it takes all multiple variables (main variables, demographic and other determinants) which mainly consist of either interval or categorical properties. As a side note, this study converts all categorical variables into dummy variables, in which we expand each category as a new variables scored with either one or zero.*  Furthermore, after carefully considering all demographical and other determinants that we have asked in the survey, we decide to retain all except profession. Despite all the participants are either teachers or school staff, we were not anticipating that each of the participants may or may not have second profession. This is also confusing when we found out that between those respondents who work as teachers, there were those who work in private sector (private schools) and those who work as civic servant (Indonesia government formal schools). Despite they’re all teachers or school staffs, participants may fill their profession either work in private sectors, civic servants, or teachers. Therefore, we eliminate profession variable to avoid confusion.  In reporting the regression, we also follow your suggestion to refrain from stepwise, and to include all the results without anything omitted. We do this also for each AIS subscales (S4 AIS subscales multiple regression results; Table 1-7).  Below is the syntax we use to compute the main AIS regression:  REGRESSION  /MISSING LISTWISE  /STATISTICS COEFF OUTS CI(95) R ANOVA CHANGE ZPP  /CRITERIA=PIN(.05) POUT(.10)  /NOORIGIN  /DEPENDENT AIS_ALL_ORI  /METHOD=ENTER EPQ_Ideal_ORI EPQ_Relat_ORI ROS_PersIntExt_ORI ROS_Soc_ORI dmvar1female_sex age dmvar2yes_afilR_group  dmvar6a_no_ed dmvar6b_elementary_ed dmvar6c_junhigh_ed dmvar6d_senhigh_ed dmvar6e_diploma_ed dmvar6f_bachelor_ed  dmvar6h_others_ed dmvar7a_AnimSect dmvar7b_EnvPreserv dmvar7c_HealthRights_improve dmvar8a_married dmvar8a_widower dmvar8byes_wchildren  dmvar9urban_livurban dmvar10a_apartment dmvar10b_roomrent dmvar10c_ownhouse  dmvar11yes_haveyard dmvar13c_relimportance dmvar14ab_incexactmin  dmvar14ac_incaverage dmvar14ad_inct2xaverage dmvar14ae_incmore2xaverage dmvar14af_increfuseanswer  dmvar14bb_exp5to10k dmvar14bc_exp10to15k dmvar14be_expabove25k  dmvar14bf_exprefuseanswer dmvar15a_consnomeat dmvar15c_cons23aweek  dmvar15d_cons46aweek dmvar15e_conseachday dmvar16_havepet  dmvar17a_zoo1amonth dmvar17b_zoo1semester dmvar17c_zoo1ayear dmvar17d_zoo12moreyear. |

| 13 | Line 475 seems to contradict itself, there’s no correlation between idealism and social religious orientation, but the authors accept the hypothesis that there is one? |
| --- | --- |
|  | Response:  Dear Reviewers,  This question is answered partly in our response for review #10. At first we only want to describe that despite the non-significant, the consistent negative correlation between AIS and idealism that were found in various studies (Galvin & Herzog, 1992; B Su & Martens, 2017; Bingtao Su & Martens, 2018; Wuensch & Poteat, 1998) is also captured in this study. However, we will not report it because it is not statistically significant .  Thus, we revise this (line 526 to 544) to:  *The hypothesis presented in this section is that a higher extrinsic social religious orientation (ES) correlates to lower idealism (I), higher relativism (R), and a higher acceptability for harming animals (AIS). We find only partial support to the fourth hypothesis. Table 6 shows that higher extrinsic social religious orientation correlates to a higher relativism (r[927]=0.15, p<0.01), but not to a lower idealism (r[927]=-0.02, p>0.05). In Table 7, using multiple regression, we confirm that higher extrinsic social religious orientation relates to a higher overall acceptability for harming animals in both the first (b=0.12, p<0.01) and the second model (b=0.05, p<0.05). This means that when holding all other variables constant, one point increase in ES is more likely to increase 0.12 point of AIS score in the first, but only 0.05 point in the second model. However there is one difference between both models whereby the effect-size of ES shows small effect-size toward AIS score in the first model (0.02 <= F2 < 0.15), but rather small to no effect in the second model (F2 < 0.02). For the confidence interval, if we were to re-fit both models for total of 20 random trials, taking samples of the same size from the same population, we can be confident that for 19 out of total 20 trials (95% of the time), an increase of one unit of ES will be more likely to increase AIS between 0.08 to 0.15 point in the first model, while in the second model will be more likely to increase AIS between 0.007 to 0.095 point. Therefore, except for every relations with idealism, the present study accepts all the expected main variables’ relations in the hypothesis.* |

| 14 | Line 488 = mention of figure 2 but there is no figure 2. |
| --- | --- |
|  | Response:  Dear Reviewers,  This sentence was a remnant from previous 1^st^ round major review that we forget to delete. We delete it and double-check for similar occurrences. Thank you. |

**References**

Galvin, S. L., & Herzog, H. A. (1992). Ethical Ideology, Animal Rights Activism, and Attitudes Toward the Treatment of Animals. *Ethics & Behavior*, *2*(3), 141–149. doi: 10.1207/s15327019eb0203_1

Smith, G. (2018). Step away from stepwise. *Journal of Big Data*, *5*(1), 32. doi: 10.1186/s40537-018-0143-6

Su, B, & Martens, P. (2017). Public attitudes toward animals and the influential factors in contemporary China. *Animal Welfare*, *26*(2), 239–247. doi: 10.7120/09627286.26.2.239

Su, Bingtao, & Martens, P. (2018). How Ethical Ideologies Relate to Public Attitudes toward Animals: The Dutch Case. *Anthrozoos*, *31*(2), 179–194. doi: 10.1007/s00210-018-1496-2

Whittingham, M. J., Stephens, P. A., Bradbury, R. B., & Freckleton, R. P. (2006). Why do we still use stepwise modelling in ecology and behaviour? *Journal of Animal Ecology*, *75*(5), 1182–1189. doi: 10.1111/j.1365-2656.2006.01141.x

Wuensch, K. L., & Poteat, G. M. (1998). Evaluating the morality of animal research: Effects of ethical ideology, gender, and purpose. *Journal of Social Behaviour and Personality*, *13*(1), 139–150. Retrieved from https://repository.library.georgetown.edu/handle/10822/912204
